# Multisite Proton‐Coupled Electron Transfer Facilitates Oxidative Photocatalysis in a Molecular Zr‐Based Coordination Compound

**DOI:** 10.1002/anie.202510723

**Published:** 2025-07-23

**Authors:** Mercedes Moreno‐Albarracín, Alvaro M. Rodriguez‐Jimenez, Omar Nuñez, Pablo Garrido‐Barros

**Affiliations:** ^1^ Departamento de Química Inorgánica, Facultad de Ciencias Universidad de Granada and Unidad de Excelencia en Química (UEQ) Avda. Fuente Nueva s/n Granada 18071 Spain

**Keywords:** Coordination chemistry, Homogeneous catalysis, Photoredox catalysis, Proton‐coupled electron transfer, Reaction mechanisms

## Abstract

The development of mediators that harness visible light to drive proton‐coupled electron transfer (PCET) offers a promising pathway to achieving challenging redox transformations in a more sustainable manner and with enhanced thermochemical efficiency. However, designing photocatalytic systems based on earth‐abundant metals while gaining precise control over their excited‐state reactivity remains a significant challenge. Here, deprotonation of the hydroxy ligands in the Zr₃(O)(OH)₃ nodes of a photoactive coordination cage is shown to unlock the photocatalytic oxidation of strong O─H and C─H bonds (70–100 kcal mol⁻^1^). Mechanistic investigations reveal that this oxidative process proceeds via a multisite PCET pathway involving ground‐state, pre‐association followed by a static quenching mechanism. This contrasts with the dynamic quenching mechanism governing the reductive PCET previously reported for the same system. Collectively, these findings establish an unprecedented ambipolar PCET mechanism with a new class of photocatalytic mediators based on an earth abundant metal.

## Graphical abstract

Deprotonation of the hydroxy ligand in the Zr_3_(O)(OH)_3_ nodes of a molecular cage unlocks photo‐oxidative catalysis via multisite proton‐coupled electron transfer (PCET), complementing previous findings in the reductive counterpart. This platform offers valuable insights into the excited‐state reactivity of photoredox catalysts and stands as a promising tool with unprecedented ambipolar reactivity for solar‐to‐chemical conversion.

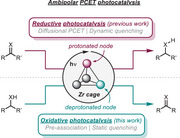

Photocatalytic proton‐coupled electron transfer (PCET) is a transformative approach to enable redox activation of inert chemical bonds, offering a promising route to renewable energy conversion.^[^ ^1^, ^2^, ^3^ ^]^ Despite its synthetic advantages, only a few molecular platforms serve as excited‐state H^+^/e^−^ donors or acceptors, highlighting the challenges in controlling excited‐state reactivity.^[^ ^1^ ^]^ This issue extends to the general field of photoredox catalysis and has motivated intensive work in this field.^[^ ^4^, ^5^, ^6^, ^7^, ^8^ ^]^ Yet, only a handful of molecular systems can efficiently catalyze both oxidative and reductive transformations,^[^ ^9^ ^]^ despite the high interest in ambipolar reactivity for chemical energy conversion.^[^ ^10^ ^]^ In addition, the presence of sacrificial reagents (e.g., exogenous acid or base) poses an additional selectivity hindrance by opening up undesired reactive pathways (Figure 1a).^[^ ^11^, ^12^, ^13^, ^14^ ^]^ While much of the foundational work in excited‐state PCET (ES‐PCET) has leveraged Ru, Re and Ir complexes for the stoichiometric oxidation of phenols as model substrates,^[^ ^15^, ^16^, ^17^, ^18^, ^19^, ^20^ ^]^ the development of molecular, photocatalytic platforms based on earth abundant metals for the oxidation of strong X–H bonds remains an exciting challenge.

**Figure 1:**
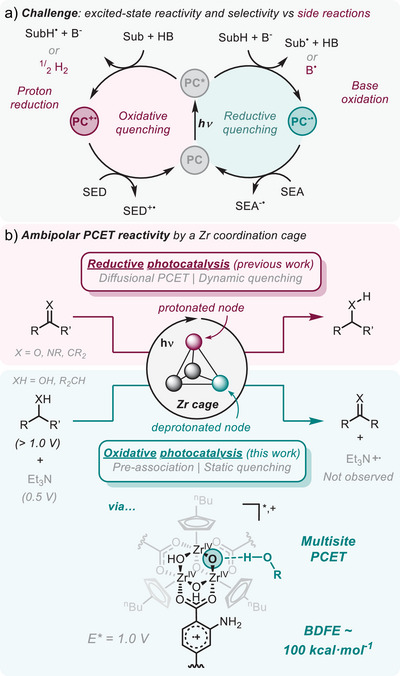
a) Reactivity pathways for the excited‐state of a photocatalyst (PC). b) PCET reactivity towards reductive (previously reported) and oxidative (this work) photocatalysis mediated by **1‐NH_2_^4+^**, featuring the mechanistic differences. Redox potentials for one electron oxidation processes are reported vs. Fc^+/0^.

We previously demonstrated that the Zr‐based coordination cage [((^nBu^CpZr)_3_(OH)_3_O)_4_(2‐aminoterephthalate)_6_]Cl_4_ (**1‐NH_2_^4+^**, ^nBu^Cp = n‐butylcyclopentadienyl) is a potent photocatalytic PCET mediator of reductive transformations (Figure 1b).^[^ ^21^ ^]^ Surprisingly, this study found no evidence for reductive quenching by sacrificial electron donors (e.g., NEt_3_ and iPrOH) despite the similarly oxidizing nature of its excited state. This observation aligns with the scarcity of reports on direct photocatalytic oxidations by Zr‐based metal‐organic compounds and with the growing development of alternative approaches based on the generation of oxidizing intermediates such as singlet O_2_ or superoxide radical species (O_2_^·−^).^[^ ^22^, ^23^, ^24^ ^]^ These collective observations, in addition to the promise of using an abundant metal such as Zr in photoredox catalysis,^[^ ^25^, ^26^, ^27^ ^]^ encouraged us to explore the factors governing the excited‐state reactivity of **1‐NH_2_^4+^**.

Our findings reveal that deprotonation of the Zr‐nodes in **1‐NH_2_^4+^** unlocks oxidation photocatalysis (Figure 1b). Notably, we found that this oxidative process relies on the pre‐association of the substrate followed by a multisite PCET mechanism, in sharp contrast to the diffusion‐controlled quenching during reductive PCET by the same platform. This reactivity establishes **1‐NH_2_^4+^** as a unique ambipolar PCET mediator for photoredox catalysis and showcases the different factors beyond the photophysical and photoredox properties that control excited‐state reactivity.

**1‐NH_2_^4+^** can absorb blue light (λ ≤ 460 nm, Figure 2) generating an excited state (1‐NH_2_^4+^)* with an associated 1‐NH_2_^4+,*^ ^/^ **^3+^** redox potential of ∼1 V vs. Fc^+/0^ (Fc is ferrocene; all potentials here are reported vs. Fc^+/0^). This value is calculated with Equation 1 using the energy gap between the zeroth vibrational levels of the ground and excited states (*E*_00_ = 2.8 eV) and the redox potential for the ground state **1‐NH_2_^4+/3+^** couple as previously reported.^[^ ^21^ ^]^ By comparison, oxidation of NEt_3_ only requires a potential of 0.5 V, providing a large driving force for the reductive quenching of (1‐NH_2_^4+^)* via electron transfer (ET, ΔG_ET_ = −0.5 eV). However, we observe a striking lack of reactivity as evidenced by both fluorescence quenching experiments,^[^ ^21^ ^]^ and ^1^H‐NMR analysis after irradiation in the presence of **1‐NH_2_^4+^** using a 440 nm LED lamp (Figure S23).

(1) $$E^{\circ }\left(1-N{H_{2}}^{4+,*/3+}\right)=E^{\circ }\left(1-N{H_{2}}^{4+/3+}\right)+E_{00}$$

**Figure 2:**
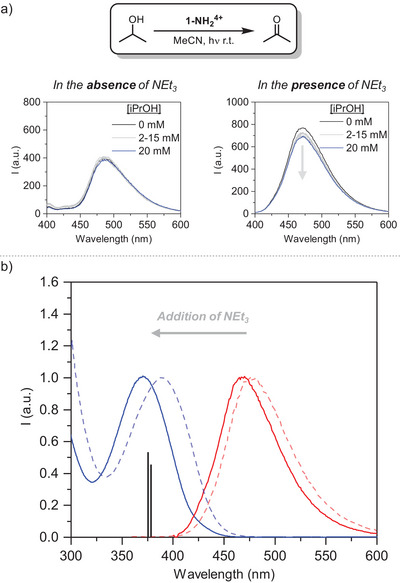
a) Reactivity and fluorescence quenching of **1‐NH_2_^4+^** in MeCN using iPrOH as the substrate with and without NEt_3_. b) Electronic absorption and emission spectra of **1‐NH_2_^4+^** in MeCN without (dashed lines) and with (solid lines) addition of NEt_3_ (100 mM), including the relevant TD‐DFT calculated electronic transitions (vertical black bars) under basic conditions.

NEt_3_ can partially deprotonate the Zr_3_(O)(OH)_3_ nodes to form Zr_3_(O)_2_(OH)_2_, causing a blueshift of the absorption and emission bands from 390 and 487 nm to 370 and 470 nm, respectively (Figure 2b). This observation is consistent with the contribution of a more energetic electron excitation from the 2‐aminoterephthalate ligand to a deprotonated Zr_3_(O)_2_(OH)_2_ node as also evidenced by time‐dependent density functional theory (TD‐DFT). Thus, the speciation exerted by the acid/base equilibrium promotes differentiation between emitting sites in their ground‐state. Using a stronger base such as triazabicyclodecene (TBD, *pK_a_* of 27 in MeCN)^[^ ^28^ ^]^ further shifts the emission due to a larger degree of cage deprotonation (Figure S9b). Despite these spectral differences, the excited state redox potential for the deprotonated cage remains similar (∼1 V) based on the *E*_00_ (2.9 eV) and **1‐NH_2_^4+/3+^** (−1.9 V) under basic conditions. In addition, time‐correlated single photon counting (TCSPC) measurements show a biexponential decay for the deprotonated cage with lifetimes of 2.9 and 11.9 ns (Figure S25), just slightly above those of the protonated homologue (1.5 and 8.4 ns).^[^ ^21^ ^]^ Biexponential fluorescence decays are typically attributed to either two distinct decay pathways or the presence of different conformers of a fluorophore. In the case of **1‐NH₂⁴⁺**, multiple isomers are present, as indicated by the broad NMR signals, which arise from the two possible orientations of the ─NH₂ group relative to the nodes (i.e., ortho and meta to each carboxylate group). These isomeric forms could result in distinct local environments, giving rise to two fluorescence lifetimes of similar magnitude.^[^ ^29^ ^]^ Overall, these favorable photochemical properties suggest the viability of reductive quenching regardless of the protonation state and thus contrast with the experimental observations.

Using iPrOH as the substrate (previously employed as an alternative SED) also failed to react with **1‐NH_2_^4+^** (Figure 2a), although this implies a thermodynamically uphill oxidation (ΔG_ET_ = 0.5 V). Strikingly, under basic conditions (excess NEt_3_), fluorescence quenching experiments revealed attenuation of the **1‐NH_2_^4+^** emission upon addition of iPrOH suggesting photochemical reactivity (Figure 2a). Using other alcohol substrates such as benzyl alcohol and 1‐phenylethanol paralleled previous observations despite the similarly uphill thermodynamics for their one‐electron oxidation (Section S9 in the SI). These results are remarkable considering that oxidation of the present NEt_3_ is highly exergonic instead.

Upon the addition of a sacrificial electron acceptor (SEA, 1000 equiv. Na_2_S_2_O_8_) and NEt_3_ as the base (1000 equiv.), irradiation in the presence of **1‐NH_2_^4+^** and either benzyl alcohol or 1‐phenylethanol substrates resulted in the catalytic formation of the corresponding benzaldehyde and acetophenone products with a turnover number (TON) between 17.5 and 35.5 (35%–71% yield; Table 1 and Figures S63–S80). The product formation was quantified by NMR and GC‐FID (Figures S64–S69). We use a conservative definition of the TON, considering that **1‐NH_2_^4+^** only mediates the initial oxidation step to generate a radical intermediate, thus the TON corresponds to the equivalents of product formed. The addition of water or oxygen to the reaction mixture resulted in lower yields (Table S1), likely due to water shifting the acid/base equilibrium toward the protonated form of **1‐NH₂⁴⁺**,^[^ ^21^ ^]^ and oxygen quenching its excited state. Increasing the substrate concentration up to 500 mM increases the TON up to 90 (Table S3). In addition, **1‐NH_2_^4+^** is also capable of promoting oxidative activation of allylic C─H bonds. Using 1,3‐cyclohexadiene and 9,10‐dihydroanthracene (DHA) under photocatalytic conditions yielded the corresponding aromatic products, benzene and anthracene with a TON of 4 and 10 (8% and 20% yield; Figures S71–S77). The reactivity of **1‐NH_2_^4+^** goes beyond 2 H^+^/2 e^−^ oxidation as exemplified using diphenylmethane and fluorene where we observed formation of the corresponding ketone products as identified by NMR and GC‐MS (5 and 8 equiv. respectively; Figures S88–S99). Once again, the addition of water or oxygen to the reaction mixture suppressed the formation of the oxidized products (Tables S4 and S5), thereby disfavoring these as adventitious sources of oxygen. Photochemical reactions conducted without Na_2_S_2_O_8_ as the SEA resulted in substoichiometric formation of the same ketone products (Figures S109–S116), suggesting that the hydroxyl groups in the Zr‐cage may act as the source of oxygen. The latter is consistent with the well‐stablished radical rebound reactivity in transition metal‐oxo catalysts.^[^ ^30^, ^31^ ^]^ However, we cannot rule out the role of Na_2_S_2_O_8_ as a source of oxygen under photocatalytic conditions. Hydroxyl transfer from **1‐NH_2_^4+^** will consequently contribute to partial cage degradation, thereby limiting the overall yield. Interestingly, the addition of 10 equiv. NaOH(aq) doubled product formation, likely due to the role of exogenous OH^−^ in regenerating the Zr‐nodes (Figures S95 and S99). Control reactions in the absence of **1‐NH_2_^4+^** failed to generate significant amounts of the corresponding products (Figures S117–S126).

**Table 1:** Photocatalytic oxidation of different organic substrates using **1‐NH_2_^4+^** under blue light irradiation.

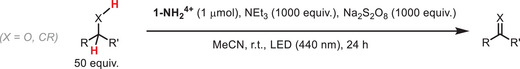							
Substrate		E° (V vs. Fc^+/0^)	p*K*_a_ (in MeCN)	BDFE_C─H_ (kcal mol^−1^)	K_SV_ (M^−1^)	Product	Equiv.^a)^, ^b)^
**1**	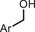					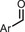	
(**a**, Ar = Ph)		1.38	47.3 (O─H)	81.7	148.3		21.0 (**a**, Ar = Ph)
(**b**, Ar = ^4‐OMe^Ph)		0.75	50.1 (O─H)	78.7	129.6		17.5 (**b**, Ar = ^4‐OMe^Ph)
**2**	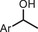					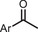	
(**a**, Ar = Ph)		1.44	48.0 (O─H)	75.7	164.0		21.0 (**a**, Ar = Ph)
(**b**, Ar = ^4‐OMe^Ph)		0.78	48.9 (O─H)	76.8	154.3		35.5 (**b**, Ar = ^4‐OMe^Ph)
(**c**, Ar = ^4‐CF3^Ph)		1.69	46.8 (O─H)	75.6	200.2		22.5 (**c**, Ar = ^4‐CF3^Ph)
**3**	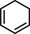	0.56	47.7 (C─H)	69.9	93.4	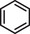	3.5
**4**	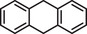	1.10	43.1 (C─H)	74.8	370.3	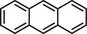	10
**5**	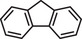	1.31	44.1 (C─H)	80.4	146.0	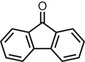	5
**6**	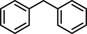	0.96	35.9 (C─H)	79.1	217.1	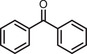	8

^a)^ Substrate concentration was 50 mM (50 equiv. relative to **1‐NH_2_^4+^**) for the photocatalytic experiments.

^b)^ TON is defined considering that **1‐NH_2_^4+^** only mediates the initial oxidation step to generate a radical intermediate, and thus, the product equiv. is equal to the TON and can be calculated as mol of product/mol of **1‐NH_2_^4+^**.

The emergence of oxidative reactivity under basic conditions suggests a PCET mechanism that circumvents an uphill initial electron transfer. One possible pathway involves a multicomponent PCET, with NEt_3_ serving as the base. However, using BnOH as the reference substrate, we found a zeroth order reaction in NEt_3_ (Figure S33) and a similar fluorescence quenching upon addition of substoichiometric amounts of NaOH (2 equiv.) to deprotonate **1‐NH_2_^4+^** avoiding excess base available (Figure S35). These experiments disfavor the involvement of NEt_3_ as an exogenous base in the PCET oxidation step. Furthermore, the lack of fluorescence quenching upon addition of Na₂S₂O₈ (Figure S61), along with substoichiometric product formation in its absence (Figures S100–S116), argues against its role in promoting the initial oxidative activation of the substrates.

Previous results point instead to a bimolecular PCET between the substrate and the excited state of the deprotonated **1‐NH_2_^4+^**, where the Zr─O─Zr bridge and the oxidized aminoterephthalate ligand act as the H^+^ and e^−^ acceptors respectively in a formal multisite transfer. Based on the *pK_a_* of **1‐NH_2_^4+^** in MeCN (∼19)^[^ ^21^, ^28^ ^]^ and the 1‐NH_2_^4+,*^ ^/^ **^3+^** redox potential in basic media (1 V), we calculate an effective bond dissociation free energy (BDFE) of 100 kcal mol^−1^ using the Bordwell equation (Equation 2), where C_G_ is the solvent dependent constant (52.6 kcal mol^−1^ in MeCN).^[^ ^32^ ^]^ This value is higher than the BDFE_X─H_ (X = O, C) of the target substrates (70–99 kcal mol^−1^), demonstrating the thermodynamic feasibility of an oxidative PCET step. To contextualize, well‐studied M^n^ = O complexes employed in ground state C─H and O─H activation feature BDFE_O─H_ for the corresponding M^n+1^─OH species between 80 and 90 kcal mol^−1^.^[^ ^33^ ^]^ In addition, combinations of Ir‐ and Ru‐based photosensitizers with phosphonate bases provide effective BDFEs ranging 92–109 kcal mol^−1^.^[^ ^34^ ^]^ This comparative analysis highlights the potential of **1‐NH_2_^4+^** for photocatalytic PCET activation of strong X─H bonds.

(2) $$BDFE=1.37\cdot pK_{a}+23.06\cdot E^{0}+C_{G}$$

(3) $$\frac{I_{0}}{\left(I_{0}-I\right)}=\frac{1}{f}+\frac{1}{fK_{SV}\left[Q\right]}$$

We turned to fluorescence quenching experiments to extract mechanistic insights into the proposed PCET step (Figure 3a and Section S9 in the SI). Stern‐Volmer analyses show a characteristic downward curvature suggesting two distinct emitting sites, only one of which is accessible to the quencher.^[^ ^35^, ^36^ ^]^ We propose that only electronic transitions near a deprotonated node enable effective quenching via oxidative PCET. In these cases, the fluorescence quenching data follows the Lehrer equation (Equation 3) where I and I_0_ represent luminescence intensity with and without the quencher, respectively, [Q] is the quencher concentration, K_SV_ is the Stern‐Volmer constant, and f denotes the fraction of accessible fluorophores.^[^ ^37^ ^]^ Plotting I_0_/(I_0_ − I) against 1/[Q] yielded an average f value of ∼0.1, supporting that only a fraction of charge transfer states facilitate oxidative PCET (Figure 3b). Notably, using TBD as a stronger base to enhance deprotonation tripled f to 0.3 (Figures S36 and S37) while a weaker base unable to deprotonate the node such as trimethylpyridine failed to promote quenching (Figure S38). In addition, using a one‐electron donor such as Ferrocene, with a stable oxidized form (i.e., Fc^+^) shows a linear Stern‐Volmer plot as expected for an ET reaction that does not depend on deprotonated nodes (Figure S62).

**Figure 3:**
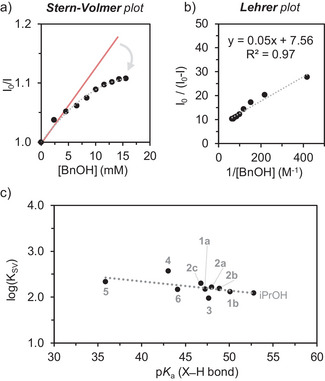
a) Stern‐Volmer and b) Lehrer plots for the fluorescence quenching upon addition of BnOH to a MeCN solution of **1‐NH_2_^4+^** with excess NEt_3_. c) Rate‐driving force relationship between the K_SV_ and the p*K*_a_ of the substrates.

The calculated K_SV_ values further revealed a noticeable p*K*_a_‐driven reactivity trend (Figures 3c and S127), contrasting with the BDFE‐driven behavior of typical thermal and photochemical PCET reactions.^[^ ^38^ ^]^ This trend could suggest stepwise H^+^/e^−^ transfers, but thermodynamic analysis disfavors this mechanism as an initial PT step is highly endothermic (∆G_PT_ > 22 kcal mol^−1^). A p*K*_a_‐driven PCET may alternatively arise from a ground‐state, pre‐association involving a non‐covalent X─H···O interaction between the Zr─O─Zr fragment and the substrate X─H bond (Figure 4a). A lower ∆p*K*_a_ between the donor and acceptor generally enhances the covalent character and strength of the interaction,^[^ ^39^ ^]^ enhancing its associated equilibrium constant (K_A_) and, in turn, the quenching rate (K_SV_). Infrared (IR) spectroscopy evidences this interaction by showing appreciable changes in the bands associated to the Zr─O─Zr vibrations upon addition of BnOH and DHA as model substrates (Figure 4c)^[^ ^40^ ^]^; the bands associated with the C─OH and sp^3^ C─H vibrations in these substrates respectively also undergo a noticeable shift (Figures S20 and S21). Consistent with these observations, cyclic voltammetry (CV) of **1‐NH_2_^4+^** in basic medium shows an anodic shift in the reduction peak upon addition of these substrates, which we attribute to stabilization of the reduced nodes by pre‐association (Figures S10–S16). In fact, we obtain a similar correlation between the p*K*_a_ and the equilibrium constant of the pre‐association process estimated from the anodic shift in the CV (Figure S17). Similarly, ^1^H‐NMR analysis of **1‐NH_2_^4+^** in d_3_‐MeCN with excess NEt_3_ reveals a slight downfield shift of the Cp signals with increasing substrate concentrations, supporting adduct formation (Figures S18 and S19). This pre‐association equilibrium, which is modeled via DFT calculations (Figure S129), influences the observed quenching as only a fraction of the deprotonated nodes is forming the reactive adduct. It also explains the remarkable lack of quenching and dramatically decreased reactivity in methanol due to competitive solvent binding (Figures S59, S60 and Table S2).

**Figure 4:**
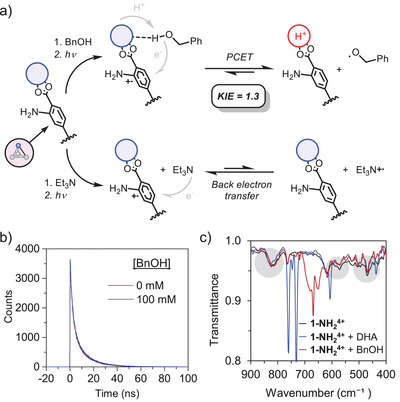
a) Mechanistic insights into the photochemical reactivity of **1‐NH_2_^4+^** with BnOH and NEt_3_. b) Lifetime measurements of **1‐NH_2_^4+^** in MeCN with excess NEt_3_ and with/without BnOH. c) ATR‐FTIR spectra of deprotonated **1‐NH_2_^4+^** in the presence of NEt_3_ and with/without BnOH and DHA substrates highlighting the regions associated to Zr─OH/O bond vibrations.

The requirement for pre‐association is typical of multisite PCET reactivity and has several implications. First, it favors static quenching as shown by the unchanged excited‐state lifetime upon addition of substrates (Figures 4b and S26). In contrast, previous examples of photochemical, oxidative PCET exhibit dynamic quenching despite the presence of pre‐association through H‐bonding.^[^ ^19^, ^41^, ^42^, ^43^ ^]^ Given the static nature of the quenching, the K_SV_ from the Lehrer equation provides an experimental measure of the pre‐association equilibrium constant, akin to the analysis by Meyer and coworkers.^[^ ^19^ ^]^ A second implication concerns the chemo‐selectivity in the case of the alcohol substrates. While the C─H bonds have lower calculated BDFEs, the O─H groups are more acidic due to their higher bond polarization, favoring the O─H···O interaction that places the O─H bond along the PCET reaction coordinate. Stern‐Volmer analysis of the deuteroxo BnOD reveals a kinetic isotope effect (KIE) ^1H^ *k*_PCET_/^2H^ *k*_PCET_ = 1.3, supporting the involvement of the O─H bond in the photochemical process (Figure S30).

The demonstrated oxidative PCET catalysis, combined with previous findings on the reductive counterpart,^[^ ^21^ ^]^ provides a comprehensive understanding of the excited‐state reactivity of **1‐NH_2_^4^⁺,** revealing its unprecedented ambipolar role in the redox activation of strong bonds (Figure 5). In its original state, **1‐NH_2_^4+^** harnesses light to selectively promote excited‐state reductive PCET, preventing H_2_ formation. However, it is incapable of undergoing effective reductive quenching for excited‐state oxidative transformations. Deprotonation of the Zr‐nodes turned off the reductive reactivity and enables oxidative PCET photocatalysis. The latter is selective over oxidation of the base employed (NEt_3_) and proceeds via static quenching, requiring the ground‐state, pre‐association with the substrate.

**Figure 5:**
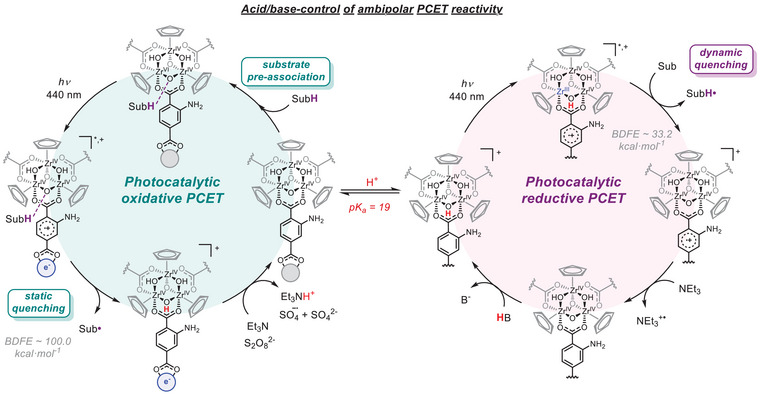
Schematic representation of the ambipolar reactivity demonstrated for the photocatalytic mediator **1‐NH_2_^4+^** towards both reductive and oxidative PCET.

Our results suggest that both C─H and O─H oxidations follow a similar mechanism, as indicated by the K_SV_–p*K*_a_ correlation and the spectroscopic and electrochemical evidence, involving noncovalent C─H···O bonding in substrates 3–6. Such interactions are less common due to the lower polarity of C─H bonds, leading to fewer examples of multisite PCET activation that rely instead on either covalent substrate–base bonding or non‐covalent pre‐association between the photo‐oxidant and a base.^[^ ^34^, ^44^ ^]^ Nonetheless C─H···O interactions have been reported and play a key role in biological systems.^[^ ^45^ ^]^ The colocalization of three oxygen atoms within the Zr_3_(O)_2_(OH)_2_ node might be a key contributing factor in the pre‐association equilibrium.

This mechanistic model also explains the lack of reactivity with NEt_3_. The aliphatic C─H bond has a much higher calculated p*K*_a_ (73.5) than previous substrates, disfavoring the formation of the reactive adduct. While direct 1e^−^ oxidation of NEt_3_ is thermodynamically feasible, the instability and oxidizing character of the resulting radical likely promotes back electron transfer (BET).^[^ ^4^, ^46^, ^47^, ^48^ ^]^ Thus, despite the high oxidizing power of **1‐NH_2_^4+^**, its net selectivity for PCET enables NEt_3_ to serve as a simple, convenient base, unlike in the case of some conventional photocatalysts.^[^ ^4^, ^11^, ^49^, ^50^, ^51^ ^]^

To conclude, we introduce a new class of excited‐state H⁺/e⁻ acceptor based on a Zr coordination cage, **1‐NH₂⁴⁺**. This cage leverages an earth‐abundant metal to achieve unparalleled photocatalytic PCET for the oxidation of strong X─H bonds (BDFE ∼ 70–100 kcalmol⁻^1^). We demonstrate that this reactivity is governed by the deprotonation of the Zr₃(O)(OH)₃ nodes and is selective against the thermodynamically more favorable oxidation of NEt₃, allowing the latter to serve as a base. Our mechanistic investigations reveal a key pre‐association equilibrium that facilitates multisite PCET through static quenching, in contrast to the dynamic quenching mechanism operative during reductive PCET in the same system. The demonstrated oxidative PCET expands upon previously established reductive reactivity, positioning **1‐NH₂⁴⁺** as an unprecedented ambipolar PCET mediator for the redox activation of strong bonds. More broadly, our findings elucidate the different pathways involved in the excited‐state reactivity, highlighting the mechanistic factors that extend beyond photophysical and photoredox properties.

## Supporting Information

The authors have cited additional references within the Supporting Information.^[^ ^52^, ^53^, ^54^, ^55^, ^56^, ^57^, ^58^, ^59^, ^60^, ^61^, ^62^, ^63^, ^64^, ^65^, ^66^, ^67^, ^68^, ^69^, ^70^, ^71^, ^72^ ^]^

## Conflict of Interests

The authors declare no conflict of interest.

## Supporting information



Supporting Information

## Data Availability

The data that support the findings of this study are available in the Supporting Information of this article.
