# Supplementary material for: Multisite Proton‐Coupled Electron Transfer Facilitates Oxidative Photocatalysis in a Molecular Zr‐Based Coordination Compound
Source: Angew Chem Int Ed Engl. 2025 Jul 23;64(38):e202510723. doi: 10.1002/anie.202510723 (PMC12435420; doi:10.1002/anie.202510723)
Supplement: Supplementary file 1 — Supporting Information [file ANIE-64-e202510723-s001.pdf]

Supporting Information for

Multisite Proton-Coupled Electron Transfer Facilitates Oxidative Photocatalysis in a Molecular Zr-Based Coordination Compound

Mercedes Moreno-Albarracín,[a] Alvaro M. Rodriguez-Jimenez,[a] Omar Nuñez[a], and Pablo Garrido-Barros*[a]

Departamento de Química Inorgánica, Facultad de Ciencias, Universidad de Granada and Unidad de Excelencia en Química (UEQ), Avda. Fuente Nueva s/n, 18071, Granada, Spain. E-mail: pgarridobarros@ugr.es

Table of contents

S.1. General considerations. 3

S.2. Synthesis of materials. 5

S.3. UV-Vis spectroscopy. 9

S.4. Luminescence.. 11

S.5. Electrochemistry. 12

S.6. Spectroscopic evidence for substrate pre-association. 17

S.7. Stoichiometric photochemical reactivity with NEt_3. 21

S.8. Lifetime measurements. 22

S.9. Stern-Volmer. 24

S.10. Photocatalytic reaction. 45

S11. Correlation Rate-Driving Force. 78

S.12. DFT calculations. 80

S13. References.. 87

S.1. General considerations.

S.1.1. Materials and reagents.

Chemical reagents and solvents were purchased at commercial sources and used without additional purification. Dried solvents were purchased from Sigma Aldrich and sparged with N_2 before their use. CD_3CN and $CDCl_3$ solvents (D, 99.9% with a purity of 99.5%) were purchased from Cambridge Isotope Laboratories, Inc., and used as received. Teflon-coated magnetic stir bars were soaked in concentrated nitric acid for at least 1 h, washed repeatedly with deionized water then acetone and dried in an oven prior to use.

S.1.2. Physical and chemical methods of characterization.

^{1}H Nuclear Magnetic Resonance Spectroscopy (NMR) data were recorded on a 400 MHz BRUKER Nanobay Avance III HD High-Definition spectrometer and the spectra were internally referenced to solvent signals

Gas Chromatography – Mass Spectrometry (GC-MS) chromatogram and spectra were collected on an Agilent 8890 GC System with an Agilent 5977C GC/MSD mass detector.

UV-Vis spectroscopy was performed in a SHUMADZU UV-1800 UV/VIS Scanning Spectrophotometer.

Electrochemistry was performed using a Biologic VSP Potentiostat Galvanostat, a one-compartment three-electrode cell, a glassy carbon (GC) disk as the working electrode, a Pt disk as the counter electrode, and an Ag/AgOTf (5 mM) reference electrode. Details for the CVs are noted as they appear. $E_{1/2}$ values for the reversible waves were obtained from the half potential between the oxidative and reductive peaks. All the reported potentials are referenced to the ferrocenium/ferrocene couple ($Fc^{+/0}$), which has been used as an internal standard. The GC disk electrode for cyclic voltammetry was polished using 1, 0.3 and 0.05 μm alumina powder.

Steady-state luminescence was recorded on a Cary Eclipse Fluorescence Spectrometer. Excitation was provided by a 450 W Xe arc lamp, wavelength-selected with a 0.25 m monochromator. Luminescence was collected at 90° with reflective optics. All spectra were corrected for instrument response.

Fourier Transform Infrared Spectroscopy (FTIR) measurements were obtained with a Bruker spectrophotometer with an ATR module.

Photoluminescence lifetime was performed in a UV-VIS-PTI QuantaMaster™ 8000 spectrofluorometer (time-correlated single photon counting (TCSPC) technique) equipped with a Picosecond Photon Detector (230-850nm, PPD-850, HORIBA Scientific) and a nanoled excitation source (375nm, 700 picoseconds pulse, USHIO).

S.1.3. Computational details.

All DFT calculations were performed in the Gaussian 09,[1] using the TPSS (meta-GGA)[2] functional with def2-TZVP[3,4] on all atoms and SMD[5] implicit solvation modelling methanol for thermochemical parameters (for direct comparison with experimental available data). Geometry optimizations were computed in solution without symmetry restrictions. All calculated structures were stationary points as confirmed by single-point vibrational frequency calculations. Free energy corrections were calculated at 298.15 K and 105 Pa pressure, including zero-point energy corrections (ZPE). Unless otherwise mentioned, all reported energy values are free energies in solution under standard state conditions. The DFT calculations with the optimized xyz coordinates are available in the section S12 of this SI and in the ioChem – BD database at the DOI: http://dx.doi.org/10.19061/iochem-bd-6-517.[6]

S.2. Synthesis of materials.

The synthesis of [((^{nBu}CpZr)$_3$(OH)$_3$O)$_4$(2-aminoterephtalate)$_6$]Cl$_4$ **(1-NH$_2$$^{4+}$)**,[7] 1-Phenylethanol,[8] 1-[4-(Trifluoromethyl)phenyl]ethanol,[8] 1-(4-Methoxyphenyl)ethanol,[8] deuterated Benzyl alcohol,[9] were carried out following the established procedures previously described in the literature. Characterization of the resulting materials was done based on typical spectroscopical analysis and comparison with the reported data.

S.2.1. ^{1}H-NMR Synthesis 1-Phenylethanol.

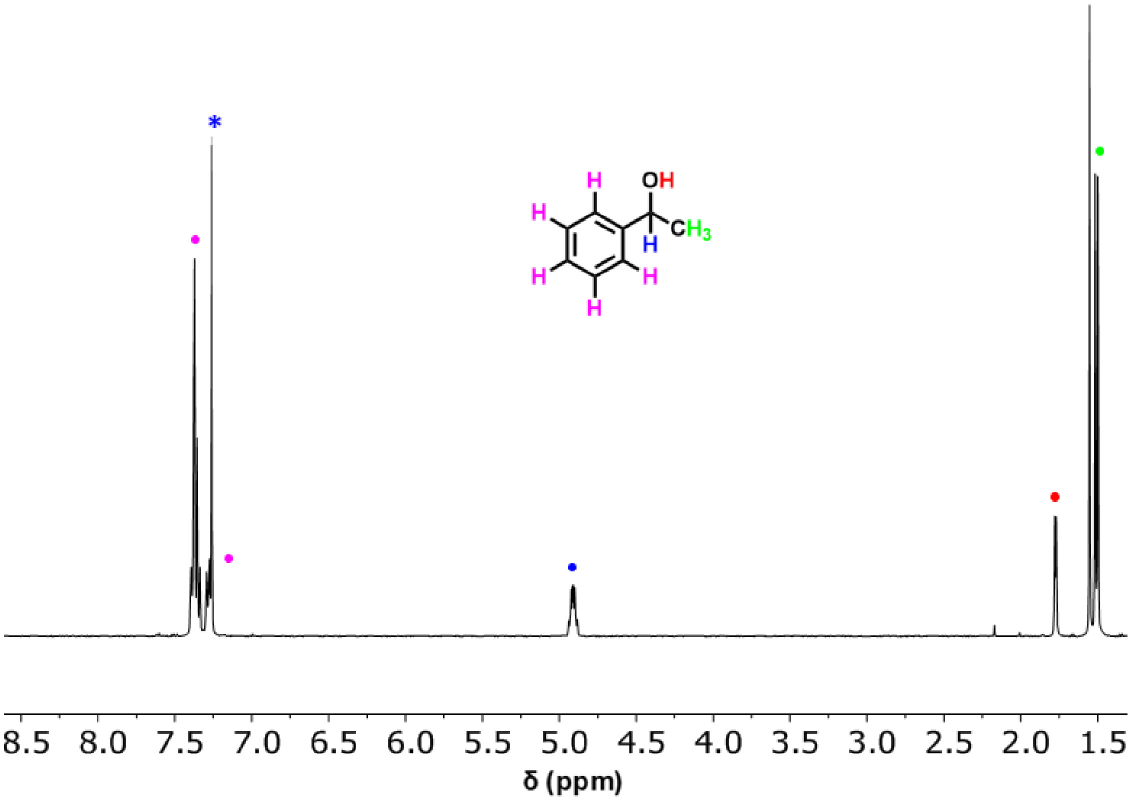

Figure S1. ^{1}H-NMR spectra (400 MHz) for the synthesis of 1-Phenylethanol. The signal marked with blue asterisk correspond to the solvent chloroform-d. Coincident with reported spectrum.

S.2.2. ^{1}H-NMR for the synthesis of the deuterated Benzyl alcohol.

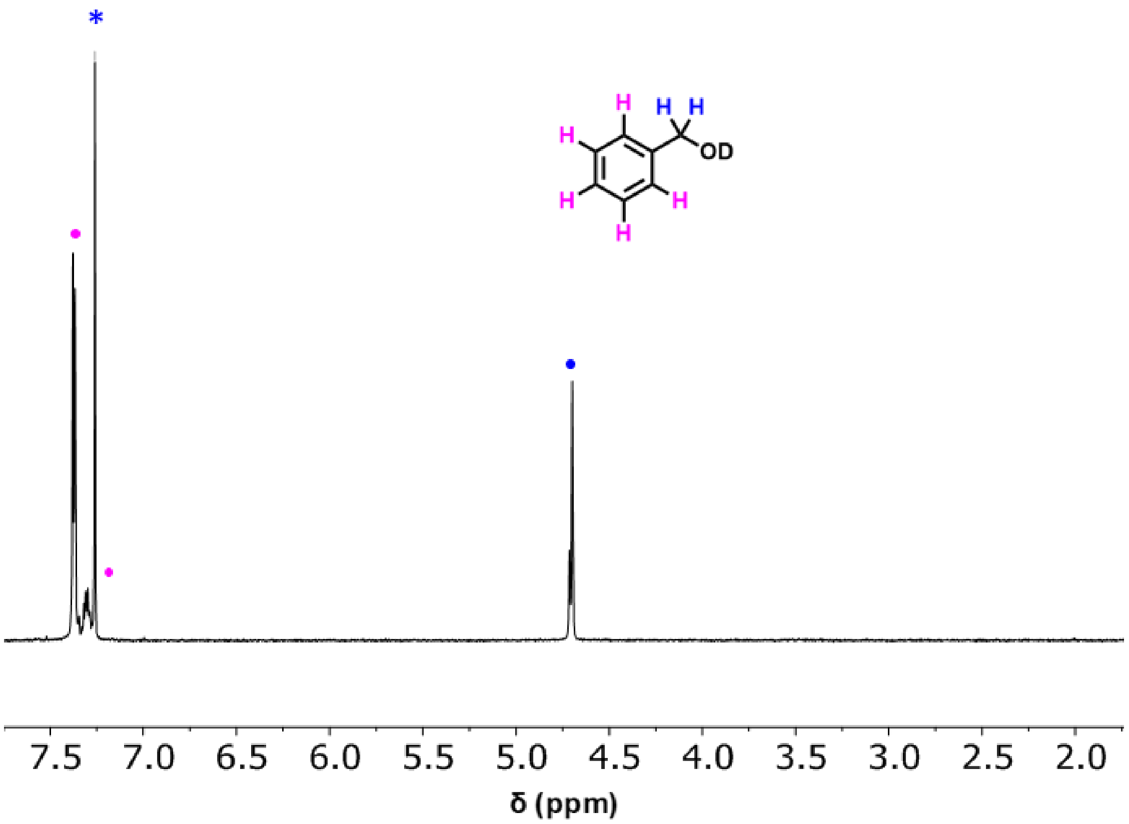

Figure S2. ^{1}H-NMR spectra (400 MHz) for the synthesis of deuterated Benzyl alcohol. The signal marked with blue asterisk correspond to the solvent chloroform-d. Coincident with reported spectrum, demonstrating the absence of the signal at 2.3 ppm reported for the OH proton.

S.2.3. Procedure for the synthesis of 1-(4-Methoxyphenyl)ethanol and ^{1}H-NRM.

Sodium borohydride (0.0420 g, 1.10 mmol) was added to a stirred solution of 4-Methoxyacetophenone (0.150 g, 1.00 mmol) in absolute EtOH (10 ml). After stirring for 5 hrs the reaction mixture was concentrated in vacuo and the residue dissolved in DCM (50 ml). The organic layer was subsequently washed with distilled water (3 x 50 ml), the organic extract dried over $MgSO_4$ and concentrated in vacuo to afford the respective alcohol.

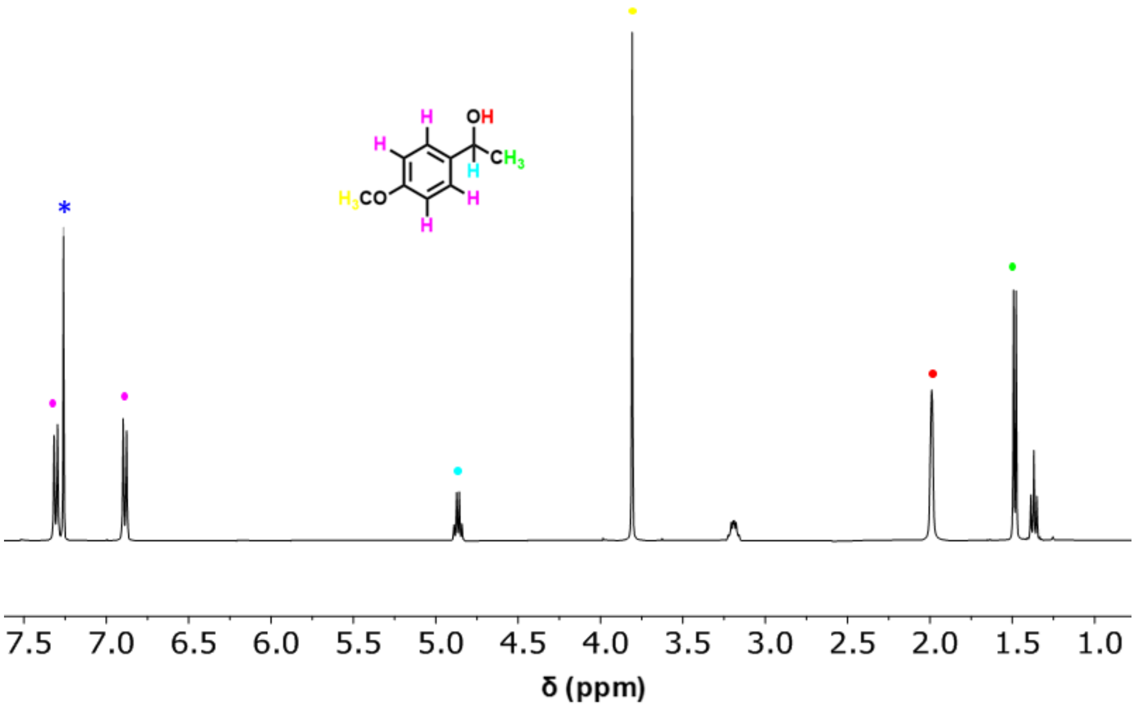

Figure S3. ^{1}H-NMR spectra (400 MHz) for the synthesis of 1-(4-Methoxyphenyl)ethanol. The signal marked with blue asterisk correspond to the solvent chloroform-d. Coincident with reported spectrum.

S.2.4. ¹H-NMR of the synthesis of 1-[4-(Trifluoromethyl)phenyl]ethanol.

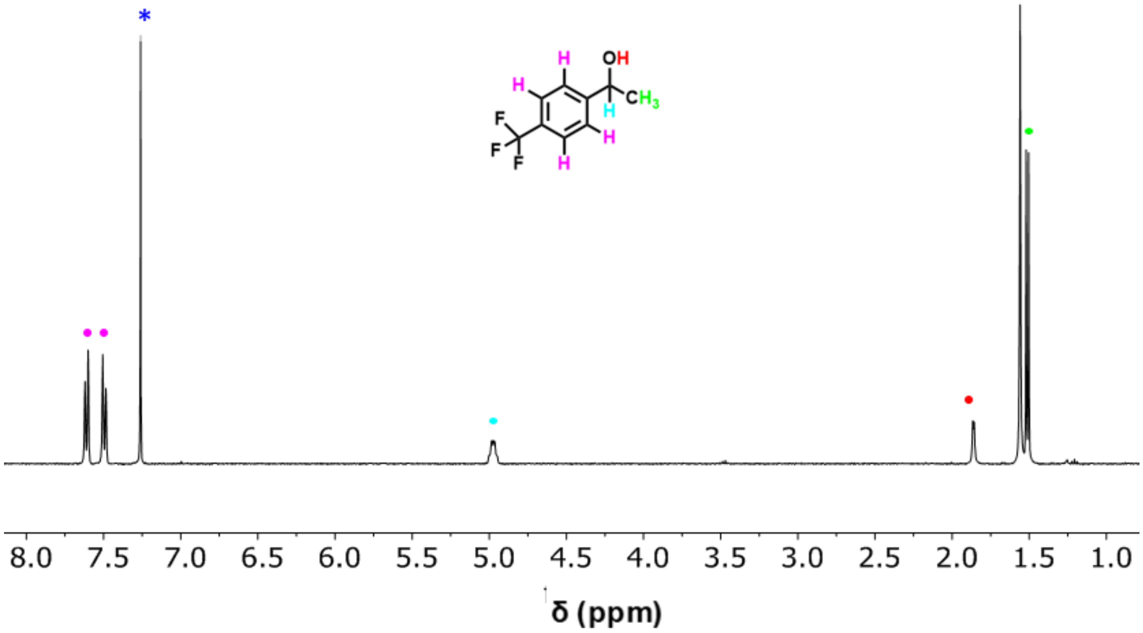

Figure S4. ¹H-NMR spectra (400 MHz) for the synthesis of 1-[4-(Trifluoromethyl)phenyl]ethanol. The signal marked with blue asterisk correspond to the solvent chloroform-d. Coincident with reported spectrum.

S.3. UV-Vis spectroscopy.

S.3.1. UV-vis 1-NH_2^{4+} with and without NEt_3 in methanol.

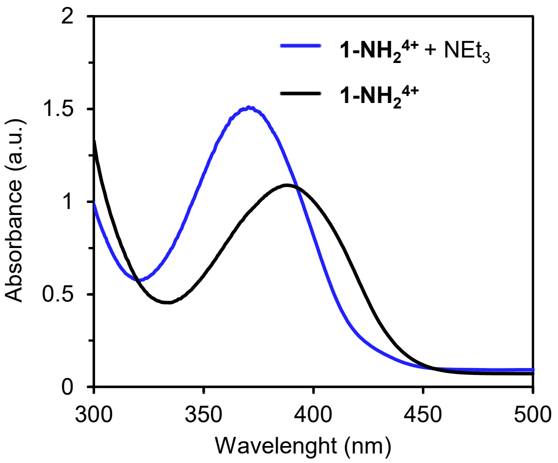

Figure S5. UV-vis absorbance of **1-NH_2^{4+}** (0.01 mM) in methanol with subsequent addition of 100 equiv. of base NEt_3 (Blue) and **1-NH_2^{4+}** (0.01 mM) in methanol without base NEt_3 (Black).

S.3.2. UV-vis 1-NH_2^{4+} with NEt_3 in acetonitrile.

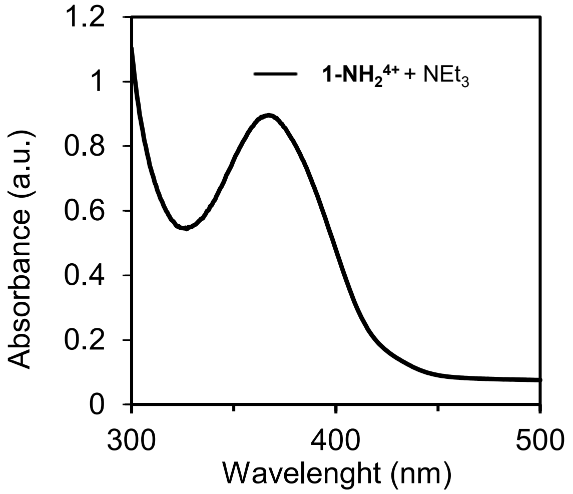

Figure S6. UV-vis absorbance of **1-NH_2^{4+}** (0.01 mM) in acetonitrile with subsequent addition of a 100 equiv. base NEt_3.

S.3.3. UV-vis addition Benzyl alcohol.

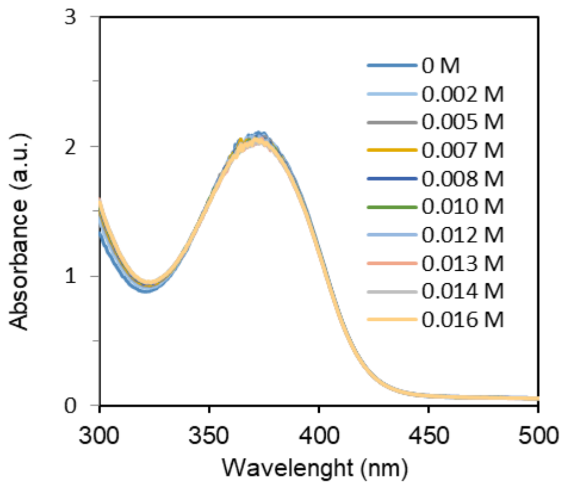

Figure S7. UV-vis absorbance of **1-NH$_2$$^{4+}$** (0.01 mM) with 100 equiv. base NEt_3 in methanol with subsequent addition of Benzyl alcohol.

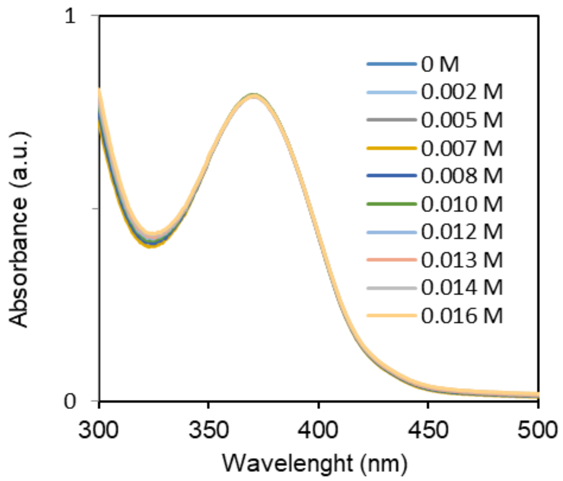

Figure S8. UV-vis absorbance of **1-NH$_2$$^{4+}$** (0.01 mM) with 100 equiv. base NEt_3 in acetonitrile with subsequent addition of Benzyl alcohol.

S.4. Luminescence.

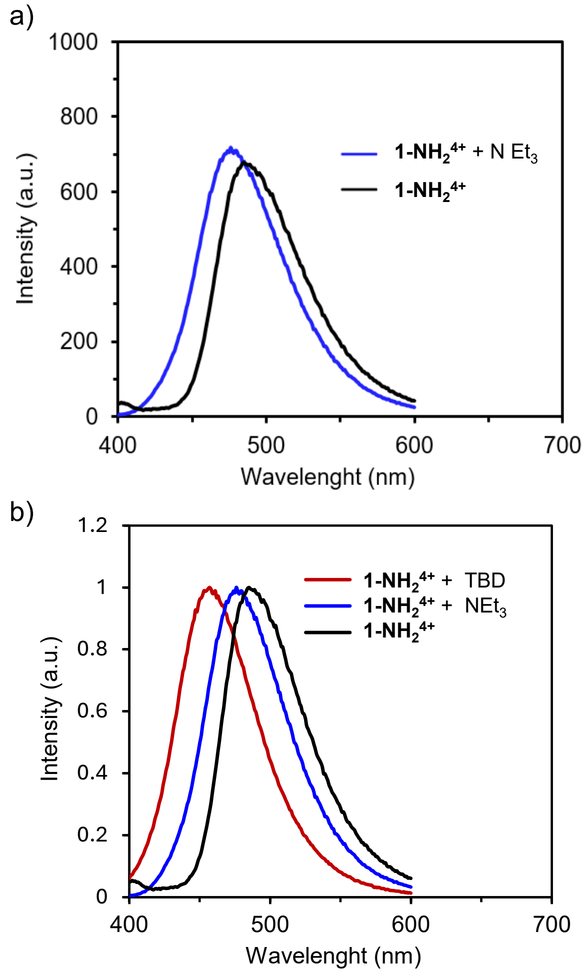

Figure S9. (a) Fluorescence emission spectra of the **1-NH$_2^{4+}$** with and without 100 equiv. base NEt_3 in acetonitrile. (b) Normalized fluorescence emission spectra of the **1-NH$_2^{4+}$** with and without 100 equiv. base NEt_3 and Triazabicyclodecene (TBD) in acetonitrile. The excitation wavelength was 350 nm.

S.5. Electrochemistry.

In a 26 mL glass vial, 12 mg of **1-NH$_2$$^{4+}$** (0.1M) and 194 mg of Tetrabutylammonium hexafluorophosphate (0.1 M) were dissolved in 5 mL of anhydrous acetonitrile. The solution was then put under a constant flow of N_2 during 15 min before measurement to eliminate the presence of O_2.

For the estimation of the K_A of the pre-association process, we have used the shift in the redox potential observed upon addition of substrate to the solution of deprotonated **1-NH$_2$$^{4+}$**. The pre-association step followed by reduction of the Zr-cage can be modelled as a CE mechanism where C is the chemical step involving pre-association and the E is electron transfer step. In these cases, for a reversible and quick chemical process such as the pre-association step, the redox potential of the observed wave follows the equation Eq. 1, where E^0_{adduct} is the potential of **1-NH$_2$$^{4+}$** in the presence of substrate, $E^0_{Zr\text{-}cage}$ is the potential of **1-NH$_2$$^{4+}$** in the absence of substrate, R is the ideal gas constant, T is the temperature, F is the Faraday constant and K is the equilibrium constant.[10] Thus, by extraction of ΔE ($E^0_{adduct} - E^0_{Zr\text{-}cage}$) upon addition of a fixed amount of each substrate (in this case 5 equiv.), we calculated the equilibrium constant K and used it for correlation with the pK_a. Note that the values of K extracted from cyclic voltammetry might differ from those obtained from Stern Volmer quenching (K_{SV}) due to the different conditions (e.g., presence of electrolyte) and nature of the experiment (in this case the equilibrium is affected by potential follow-up reactions upon electrochemical reduction).

$$E^0_{adduct} = E^0_{Zr-cage} + \frac{RT}{F} K, Eq. 1$$

S.5.1. Addition of Benzyl Alcohol.

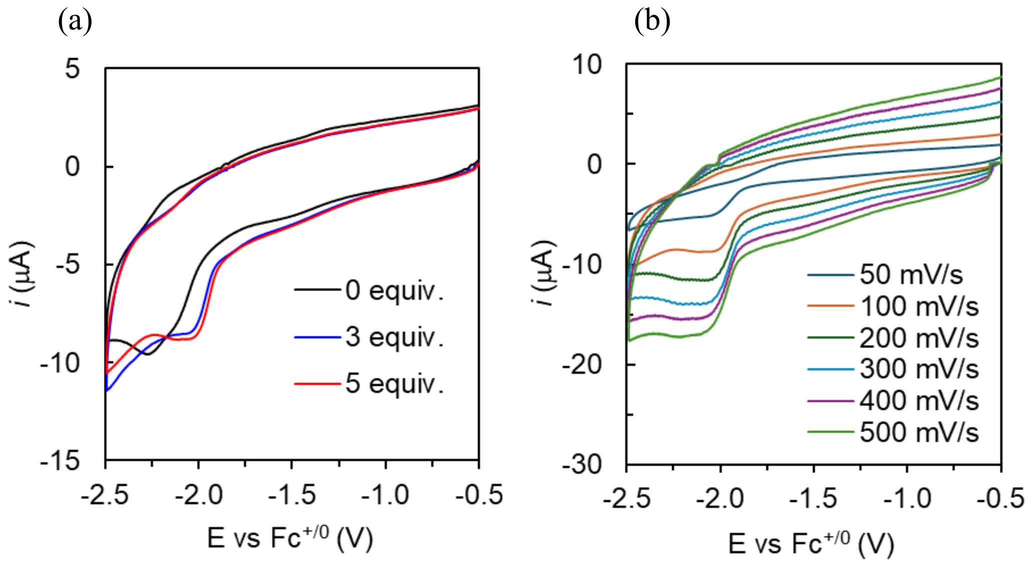

Figure S10. (a) Cyclic voltammetry of **1-NH$_2$$^{4+}$** at different equivalents of Benzyl alcohol in acetonitrile with NEt_3, using 0.1 M $TBAPF_6$ as electrolyte, a glassy carbon working electrode, an Ag/AgCl reference electrode, and a Pt counter electrode. (b) Cyclic voltammetry of **1-NH$_2$$^{4+}$** at different scan rates of five equiv. Benzyl alcohol in the same conditions.

S.5.2. Addition of different substrates.

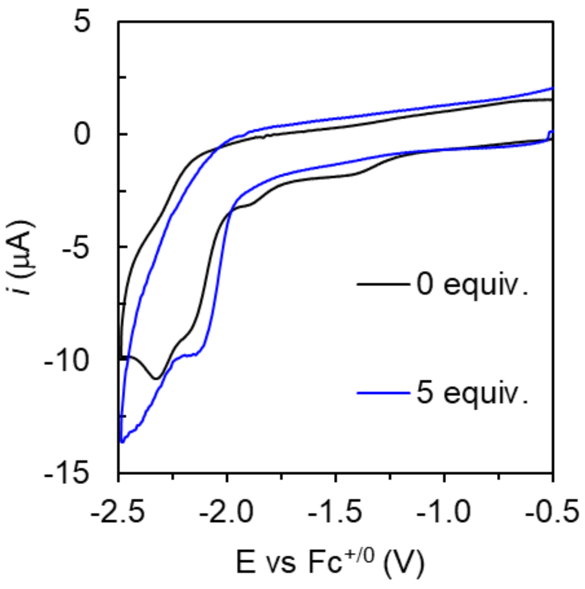

Figure S11. Cyclic voltammetry of **1-NH$_2$$^{4+}$** at different equivalents of Isopropanol in acetonitrile with NEt_3, using 0.1 M $TBAPF_6$ as electrolyte, a glassy carbon working electrode, an Ag/AgCl reference electrode, and a Pt counter electrode.

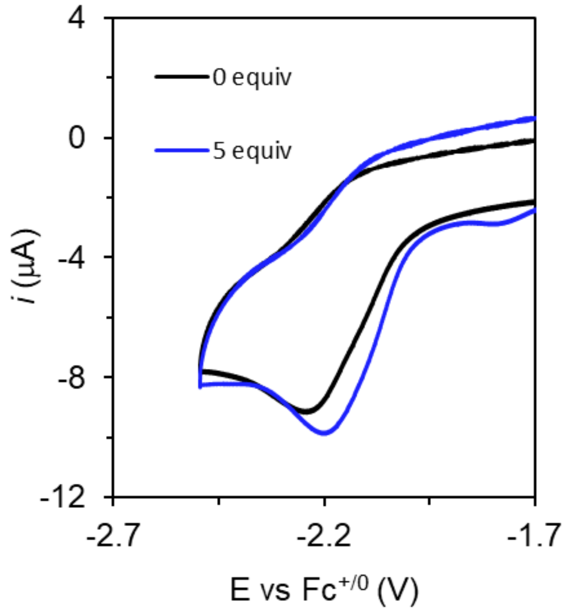

Figure S12. Cyclic voltammetry of **1-NH$_2$$^{4+}$** at different equivalents of 1-Phenylethanol in acetonitrile with NEt_3, using 0.1 M $TBAPF_6$ as electrolyte, a glassy carbon working electrode, an Ag/AgCl reference electrode, and a Pt counter electrode.

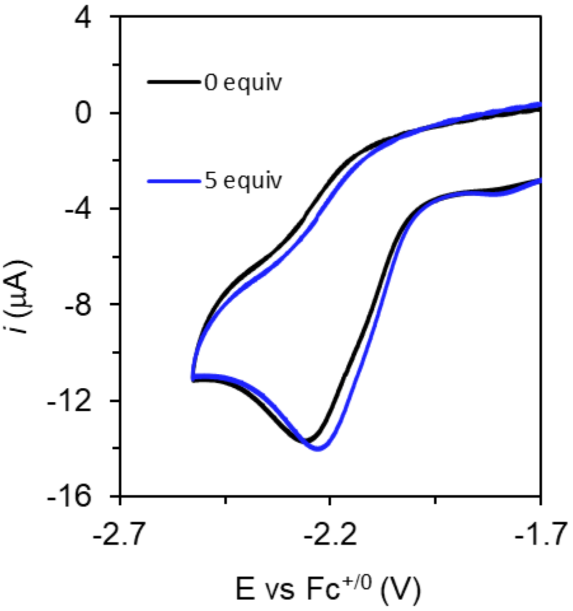

Figure S13. Cyclic voltammetry of **1-NH$_2$$^{4+}$** at different equivalents of 1,3-cyclohexadiene in acetonitrile with NEt_3, using 0.1 M $TBAPF_6$ as electrolyte, a glassy carbon working electrode, an Ag/AgCl reference electrode, and a Pt counter electrode.

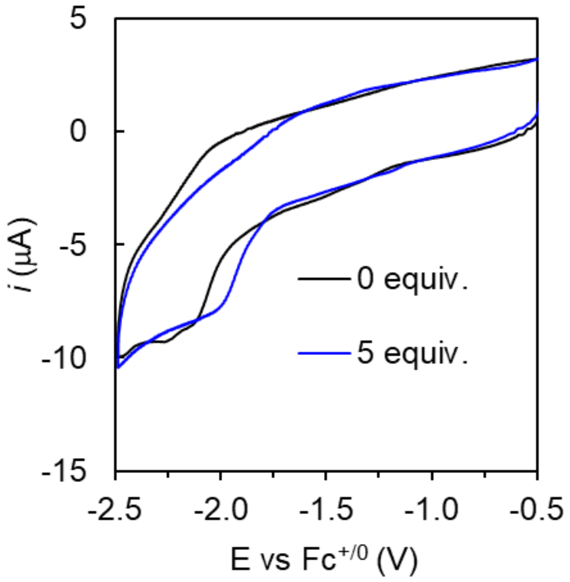

Figure S14. Cyclic voltammetry of **1-NH$_2$$^{4+}$** at different equivalents of 9,10-Dihydroanthracene in acetonitrile with NEt_3, using 0.1 M $TBAPF_6$ as electrolyte, a glassy carbon working electrode, an Ag/AgCl reference electrode, and a Pt counter electrode.

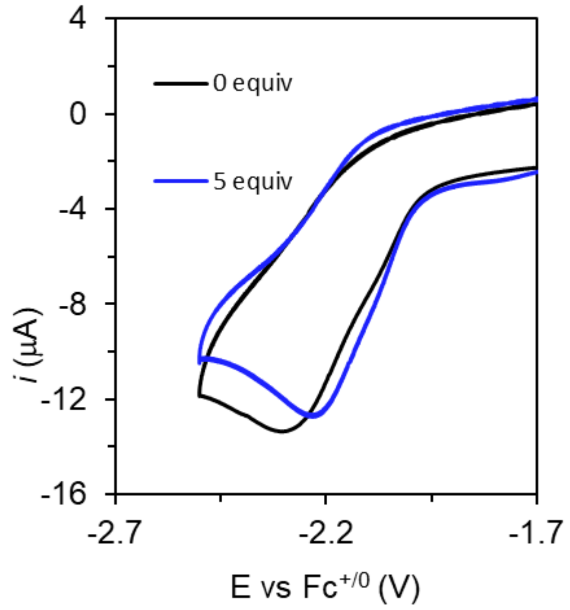

Figure S15. Cyclic voltammetry of **1-NH$_2$$^{4+}$** at different equivalents of Diphenylmethane in acetonitrile with NEt_3, using 0.1 M $TBAPF_6$ as electrolyte, a glassy carbon working electrode, an Ag/AgCl reference electrode, and a Pt counter electrode.

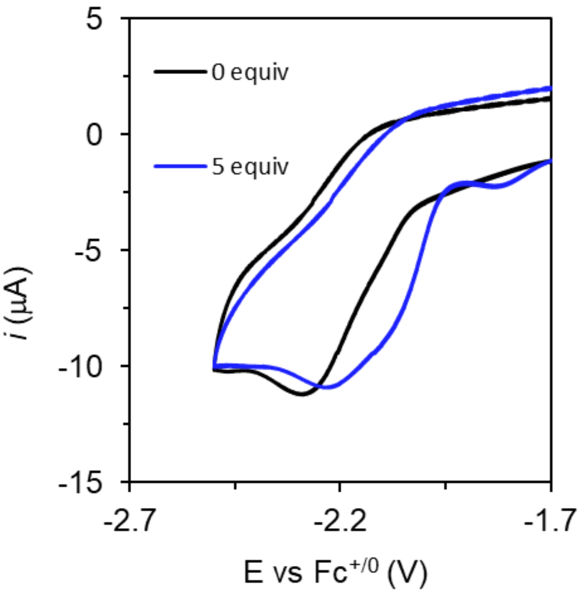

Figure S16. Cyclic voltammetry of **1-NH$_2$$^{4+}$** at different equivalents of Fluorene in acetonitrile with NEt_3, using 0.1 M $TBAPF_6$ as electrolyte, a glassy carbon working electrode, an Ag/AgCl reference electrode, and a Pt counter electrode.

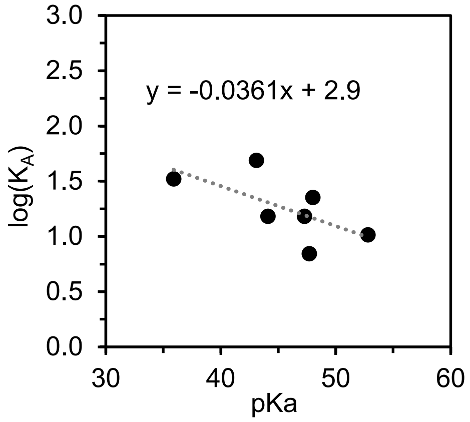

Figure S17. Correlation between the K_A for the pre-association process calculated from cyclic voltammetry and the pK_a of the substrate from DFT.

S.6. Spectroscopic evidence for substrate pre-association.

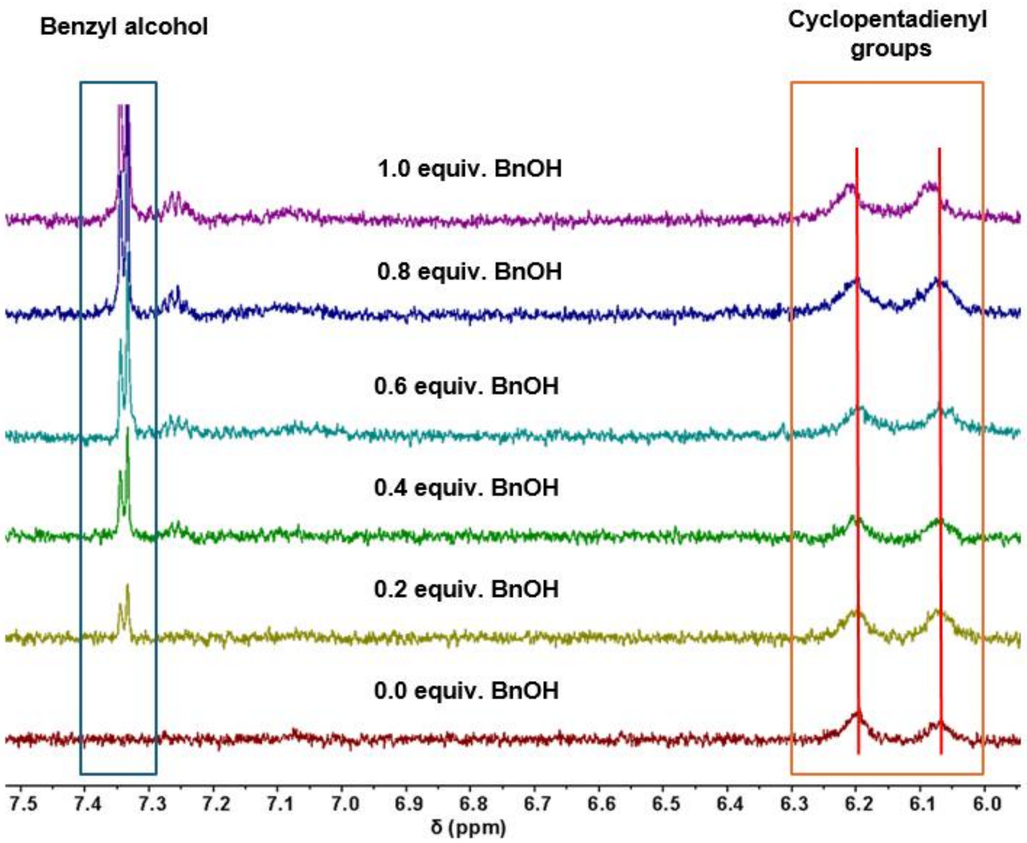

Figure S18. ^{1}H-NMR spectra (400 MHz) in acetonitrile-d3 for the characterization of the **1-NH$_2$$^{4+}$** pre-association process by addition of increasing concentrations of Benzyl alcohol in the presence of NEt_3.

Based on these data, we have estimated the association constant based on the following procedure. The total concentration of BnOH complexes was determined for each addition by integration of the aryl resonance relative to the Cp protons of the **1-NH$_2$$^{4+}$**. The relative concentrations of the associated and non-associated species were determined from the observed chemical shifts of the Cp protons relative to the shifts of pure **1-NH$_2$$^{4+}$** and **1-NH$_2$$^{4+}$** using a high excess of BnOH where no appreciable shift was further obtained (no peak shifts were observed with >10 equiv. BnOH). Then we calculated the fraction of each species and the equilibrium constant according to the following equations:

$$f(\{1-NH_2\}^{4+}) = \frac{\delta - \delta_{\{1-NH_2\}^{4+}-BnOH}}{\delta_{\{1-NH_2\}^{4+}} - \delta_{\{1-NH_2\}^{4+}-BnOH}}$$

$$[\{1-NH_2\}^{4+}] = f(\{1-NH_2\}^{4+})\cdot[\{1-NH_2\}^{4+} + \{1-NH_2\}^{4+} - BnOH]$$
$$[\{1-NH_2\}^{4+} - BnOH] = [\{1-NH_2\}^{4+} + \{1-NH_2\}^{4+} - BnOH] - [\{1-NH_2\}^{4+}]$$
$$[BnOH] = [BnOH]total - [\{1-NH_2\}^{4+} - BnOH]$$

$$K_{eq} = \frac{[\{1-NH_2\}^{4+} - BnOH]}{[\{1-NH_2\}^{4+}][BnOH]}$$

We calculated a K_{eq} of 118 ± 24 M^{-1}, in the same order of magnitude as the K_{SV} obtained from the Stern Volmer analysis with the difference between them potentially associated to inaccuracies arising from the small chemical shift changes upon pre-association.

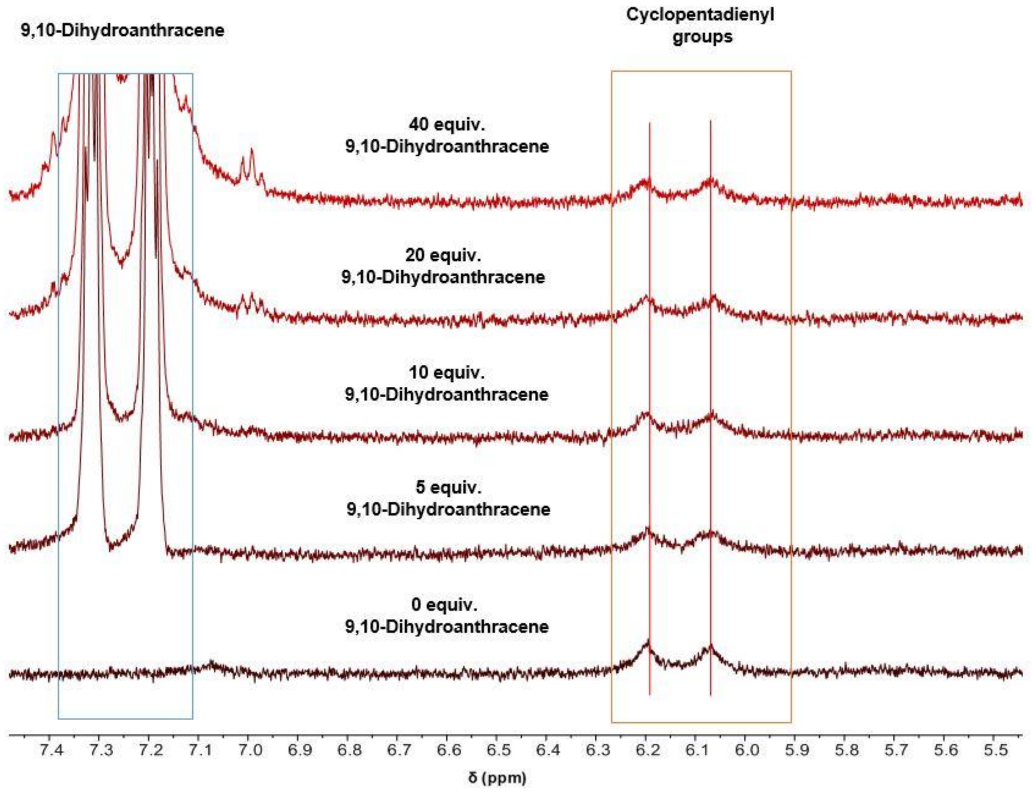

Figure S19. ^{1}H-NMR spectra (400 MHz) in acetonitrile-d3 for the characterization of the **1-NH$_2^{4+}$** pre-association process by addition of increasing concentrations of 9,10-Dihydroanthracene in the presence of NEt_3.

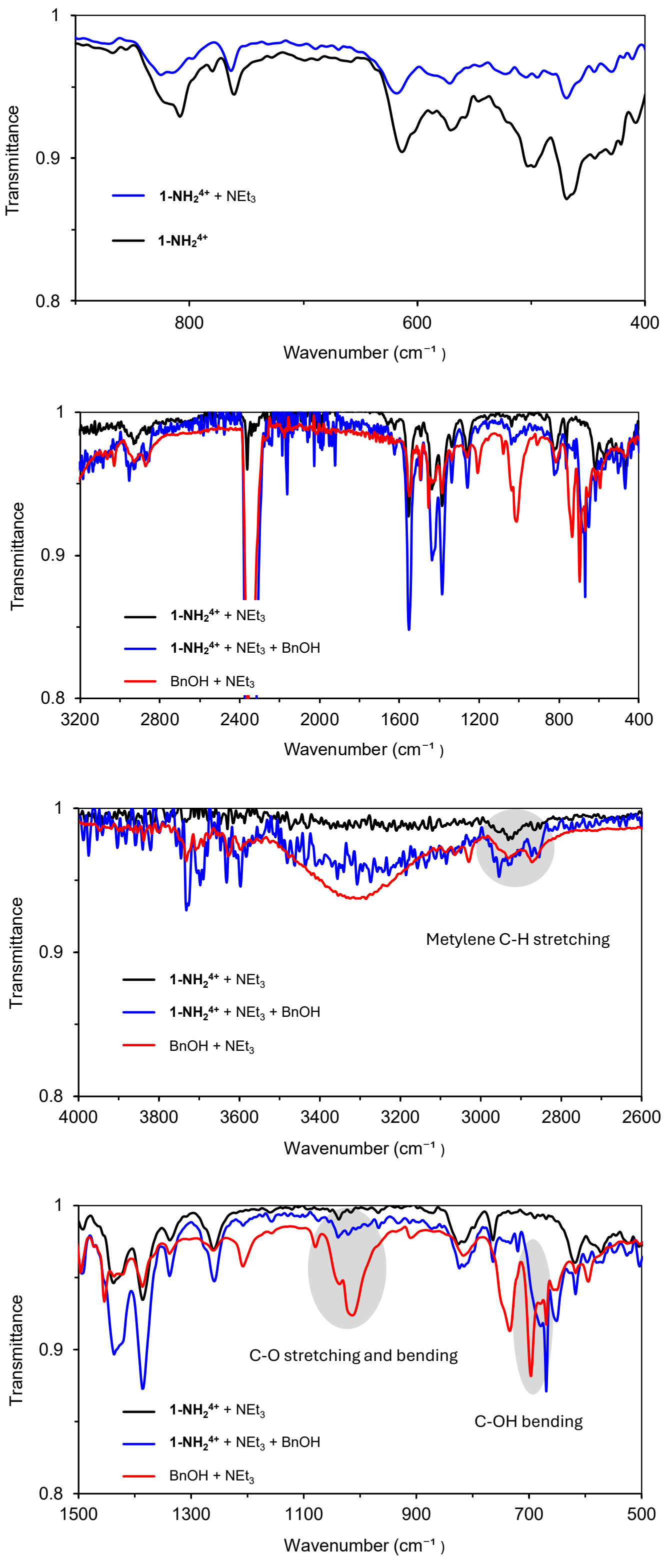

Figure S20. ATR-FTIR spectra in thin film of the deprotonated **1-NH$_2^{4+}$** in the presence of NEt$_3$ and with added Benzyl alcohol in acetonitrile. The identified regions correspond to typical vibrations of the Benzyl alcohol substrate that undergo significant changes upon interaction with **1-NH$_2^{4+}$** supporting the pre-association mechanism.[11]

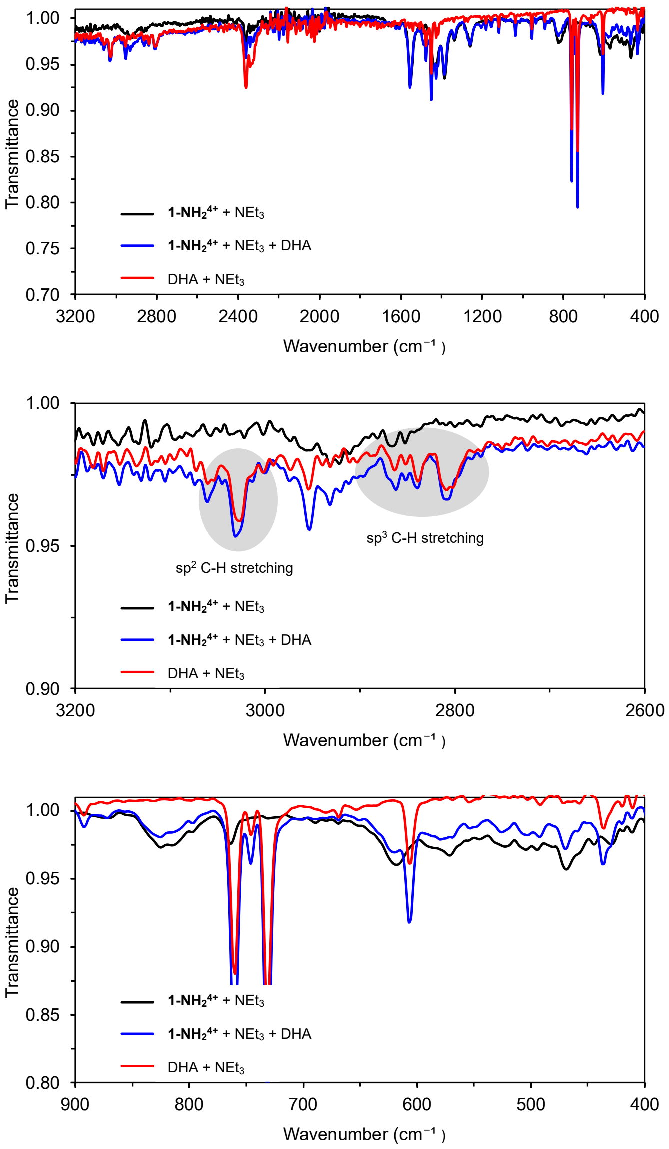

Figure S21. ATR-FTIR spectra in thin film of the deprotonated **1-NH$_2$$^{4+}$** in the presence of NEt$_3$ and with added 9,10-Dihydroanthracene in acetonitrile. The identified regions correspond to typical vibrations of the 9,10-Dihydroanthracene substrate that undergo significant changes upon interaction with **1-NH$_2$$^{4+}$** supporting the pre-association mechanism.[12]

S.7. Stoichiometric photochemical reactivity with NEt3.

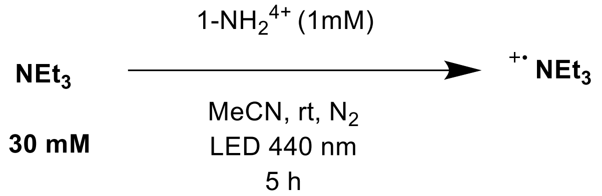

Figure S22. Scheme of photocatalytic reaction with NEt3. Addition of Maleic acid as internal standard after photoreaction.

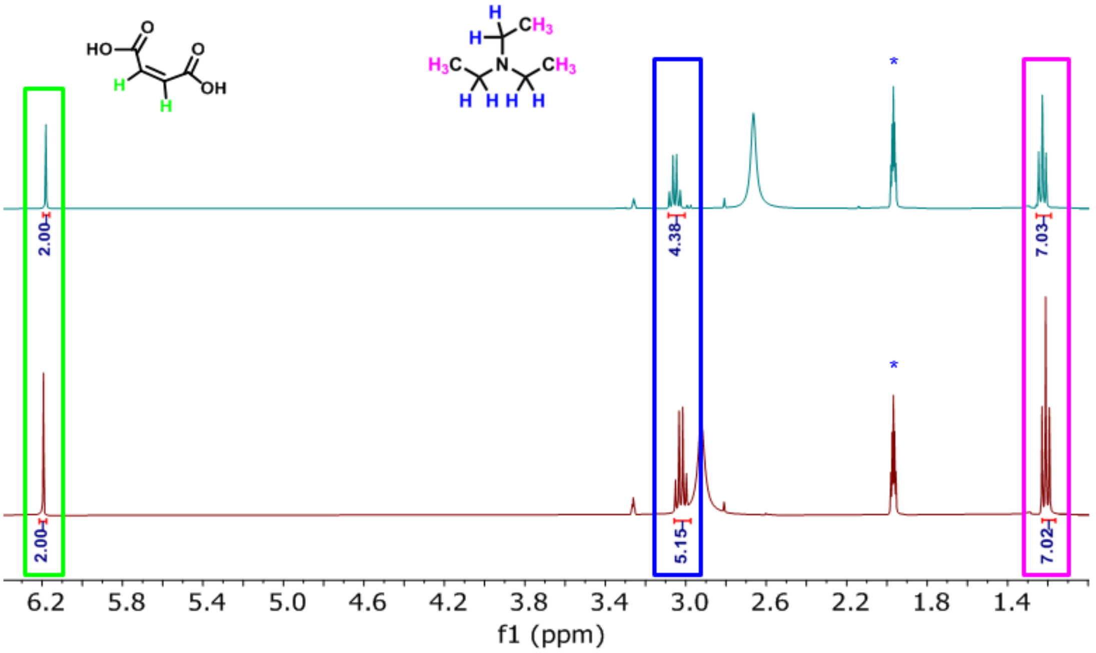

Figure S23. ^{1}H-NMR spectra (400 MHz) before (green trace) and after (red trace) photochemical reaction demonstrating that the initial amount of NEt3 remains unchanged. The signal marked with blue asterisk correspond to the solvent acetonitrile-d3.

S.8. Lifetime measurements.

S.8.1. Lifetime 1-NH_2^{4+} different equiv. of NEt_3.

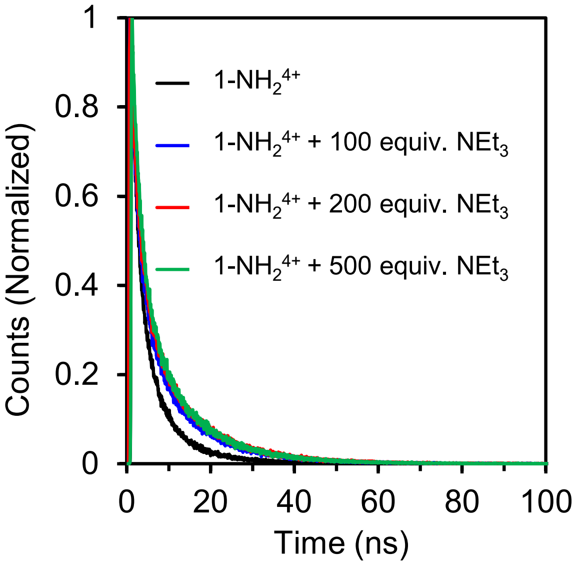

Figure S24. Decay of **1-NH_2^{4+}** emission at 480 nm after 375 nm excitation of a 20 µM solution in acetonitrile (Black) in the presence of 100 equiv. (Blue) 200 equiv. (Red) 500 equiv. (Green) NEt_3 at 20 ºC obtained with time correlated single photon counting (TCSPC) technique.

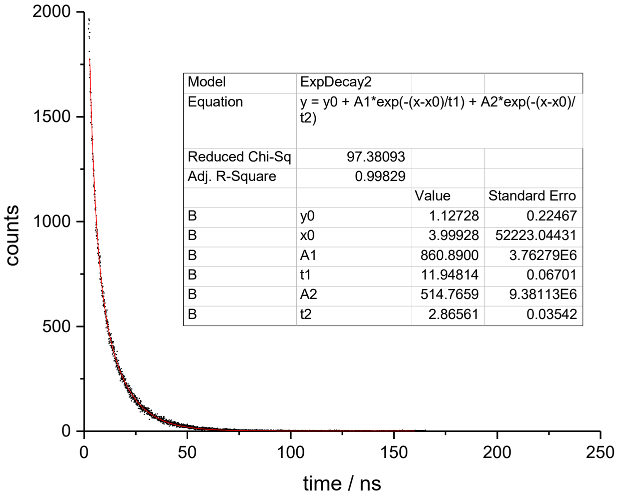

Figure S25. Decay of **1-NH_2^{4+}** emission at 480 nm after 375 nm excitation of a 20 µM solution in acetonitrile in the presence of 100 equiv. (Black). NEt_3 at 20 ºC obtained with time correlated single photon counting (TCSPC) technique. Red trace shows the fitting to a biexponential decay.

S.8.2. Lifetime 1-NH_2^{4+} and Benzyl alcohol with NEt_3.

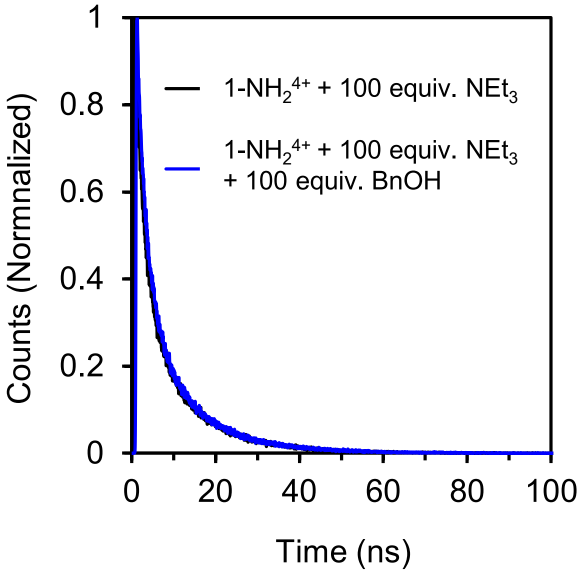

Figure S26. Decay of **1-NH_2^{4+}** emission at 480 nm after 375 nm excitation of a 20 μM solution in acetonitrile (Black) in the presence of 100 equiv. Benzyl alcohol and 100 equiv. NEt_3 (Blue) at 20 ºC obtained with time correlated single photon counting (TCSPC) technique.

S.8.3. Lifetime 1-NH_2^{4+} and 9,10-Dihydroanthracene with NEt_3.

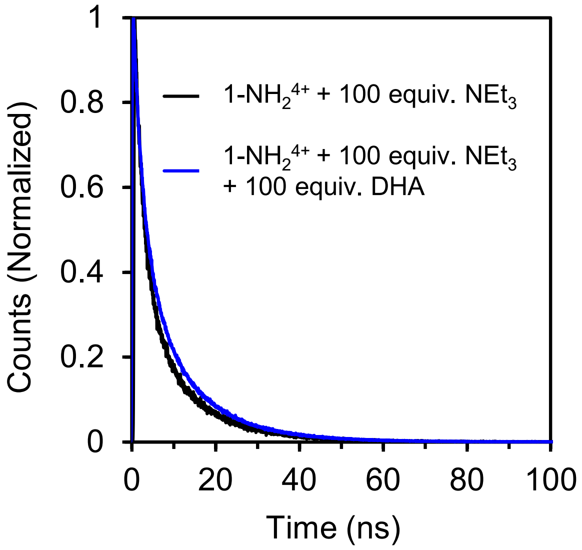

Figure S27. Decay of **1-NH_2^{4+}** emission at 480 nm after 375 nm excitation of a 20 μM solution in acetonitrile in the presence of 100 equiv. 9,10-Dihydroanthracene and 100 equiv. NEt_3 at 20 ºC obtained with time correlated single photon counting (TCSPC) technique.

S.9. Stern-Volmer.

In an Argon-filled glove box, 2 mg of **1-NH_2^{4+}** (0.5 μmol) were dissolved in 4 mL of dry acetonitrile. 2 mL were put in a fluorescence cuvette and the other 2 mL were added 100 equivalents/excess of either Benzyl alcohol with NEt_3. The cuvette and the solution were covered by a septum and taken out from the glove box in order to measure the fluorescence emission.

Stern-Volmer plots were initially made by monitoring the relationships between the initial emission intensity without quencher (I_0) and the emission intensity (I) versus the quencher concentration (Q). K_{SV} is the slope of the plot corresponding to the product between the kinetic rate of the quenching process (PCET in our case, k_{PCET}) and the lifetime of the excited state (τ_0). The resulting plot was fitted to a linear regression, from which K_{SV} can be extracted.

$$\frac{\mathrm{I_0}}{\mathrm{I}} = \mathrm{K_{SV}} \cdot [\mathrm{Q}] = \mathrm{k_{PCET}} \cdot \tau_0 \cdot [\mathrm{Q}], Eq. 2$$

Lehrer plots were employed due to the observed downward curvature in the Stern Volmer plots. We have identified the same behaviour using substrates featuring either O–H or C–H bonds. This phenomenon is typically attributed to the presence of two distinct fluorescent sites with only one of them accessible by the quencher. In these cases, the Stern-Volmer plot follows the Lehrer equation (Eq. 3) where I and I_0 is the luminescence intensity in the presence and absence of quencher respectively, [Q] is the concentration of the quencher, K_{SV} is the Stern-Volmer constant, and f is the fraction of accessible fluorophores. Plotting I_0 / (I_0 – I) versus 1 / [Q], we can extract both f and K_{SV} from the linear regression.

$$\frac{\mathrm{I_0}}{(\mathrm{I_0} - \mathrm{I})} = \frac{1}{f} + \frac{1}{f\mathrm{K_{SV}}[\mathrm{Q}]}, Eq. 3$$

S.9.1 Benzyl alcohol addition.

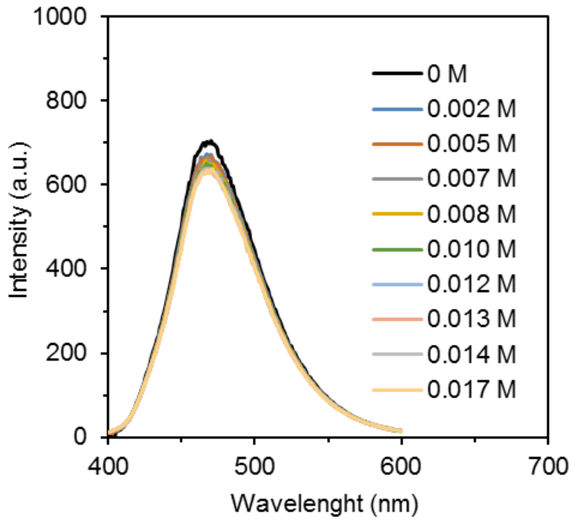

Figure S28. Fluorescence emission spectra of **1-NH$_2^{4+}$** (0.2 mM) in acetonitrile and in the presence of increasing [Benzyl alcohol]. The excitation wavelength was 350 nm.

(a) (b)

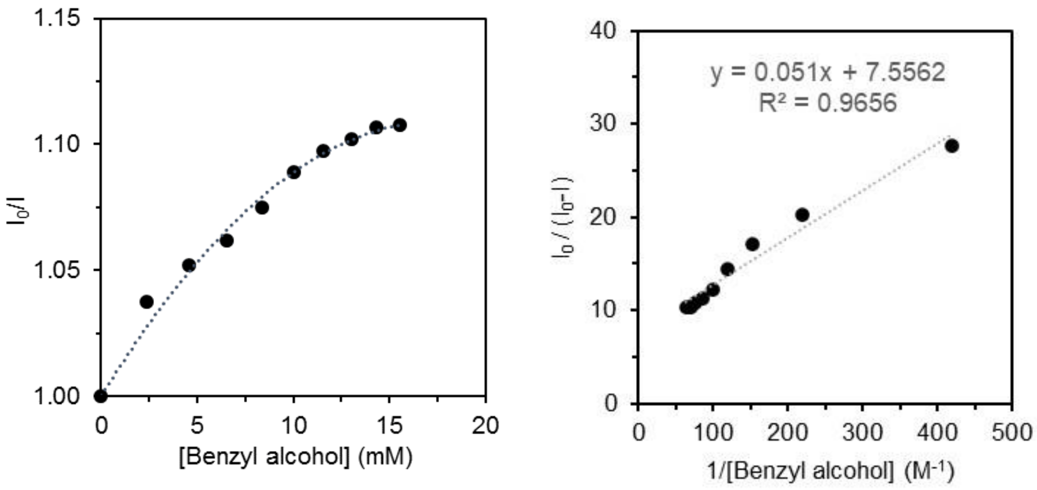

Figure S29. a) Stern-Volmer plot of I_0/I versus quencher concentration (Benzyl alcohol) under previous conditions. b) Lehrer plot of $I_0/(I_0\text{-}I)$ versus quencher concentration (Benzyl alcohol) under previous conditions.

(a) (b)

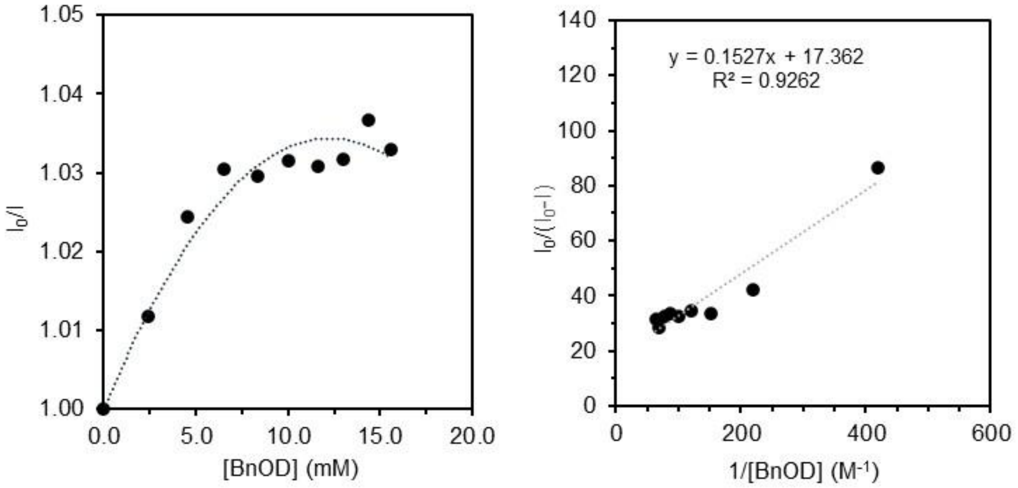

Figure S30. a) Stern-Volmer plot of I_0/I versus quencher concentration deutered Benzyl alcohol (BnOD) under previous conditions. b) Lehrer plot of $I_0/(I_0\text{-}I)$ versus quencher concentration (BnOD) under previous conditions.

S.9.2. Benzyl alcohol in presence H_2O.

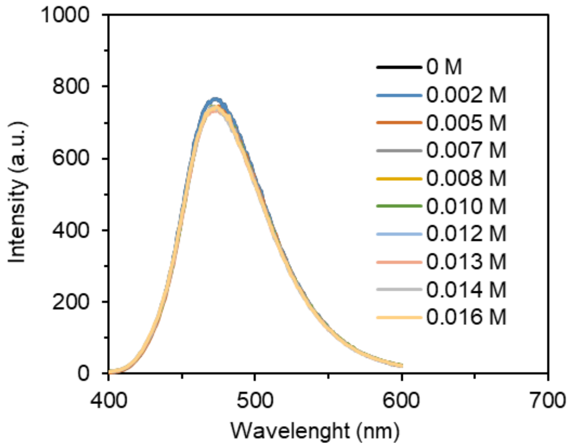

Figure S31. Fluorescence emission spectra of **1-NH$_2^{4+}$** (0.2 mM) in acetonitrile and H_2O in the presence of increasing [Benzyl alcohol]. The excitation wavelength was 350 nm.

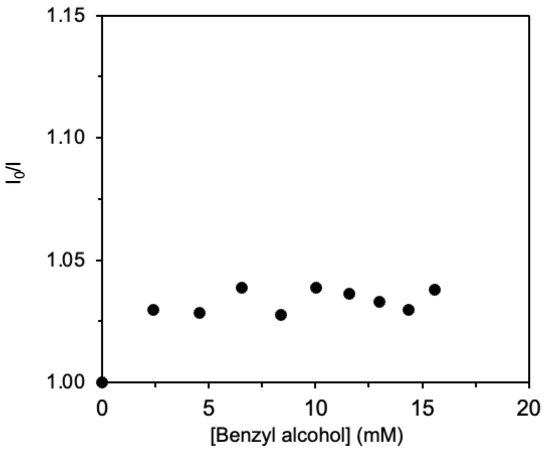

Figure S32. Stern-Volmer plot of I_0/I versus quencher concentration (Benzyl alcohol) under previous conditions.

S.9.3. Base effect.

S.9.3.1. Dependence on [NEt3].

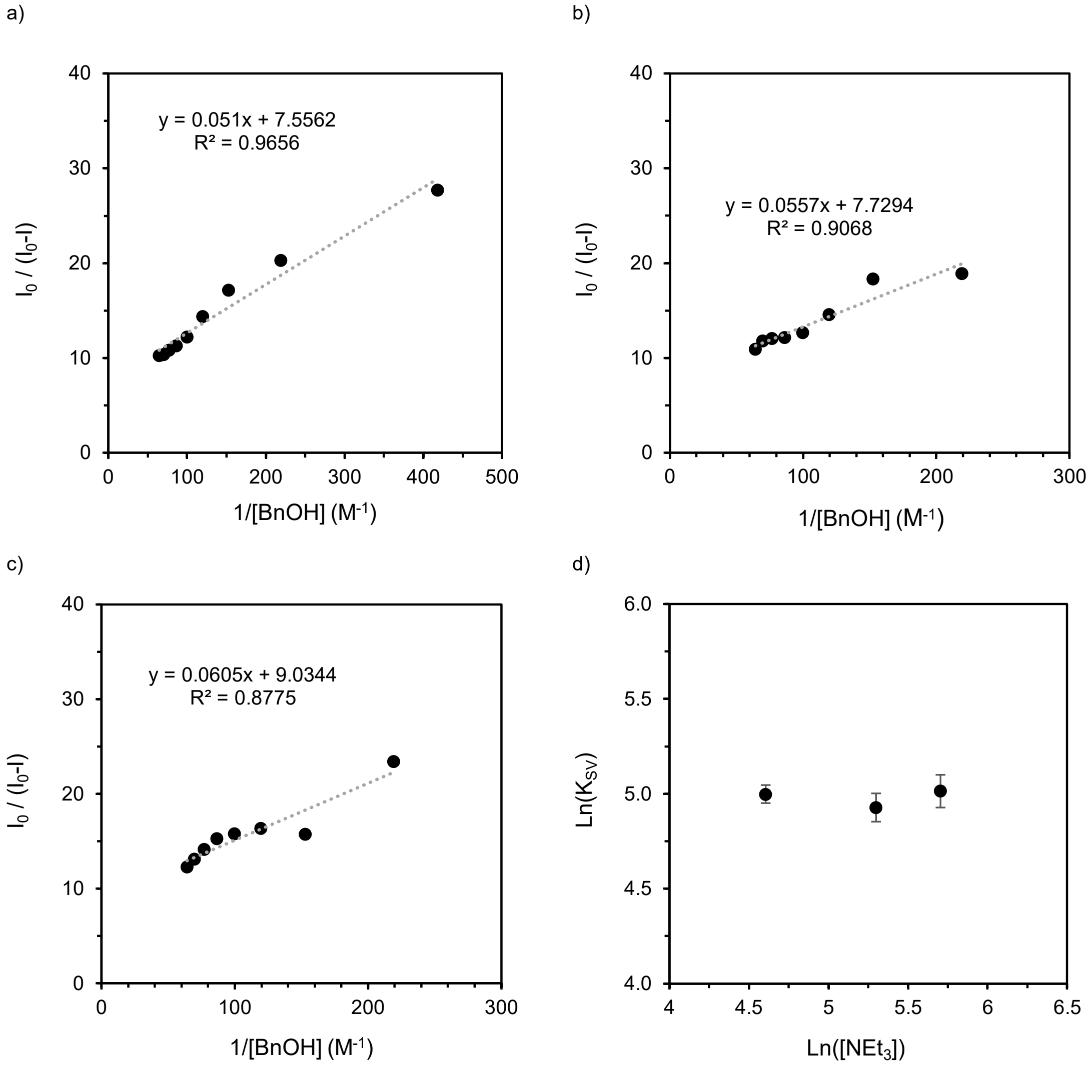

Figure S33. Lehrer plot of $I_0/(I_0\text{-}I)$ versus quencher concentration (Benzyl alcohol) in a acetonitrile solution containing **1-NH$_2^{4+}$** (0.2 mM) and in the presence of different concentrations of NEt_3: (a) 100 mM, (b) 200 mM, (c) 300 mM. The excitation wavelength was 350 nm. (d) Analysis of the K_{SV} dependence on the concentration of NEt_3.

S.9.3.2. Benzyl alcohol in presence 2 equiv. NaOH.

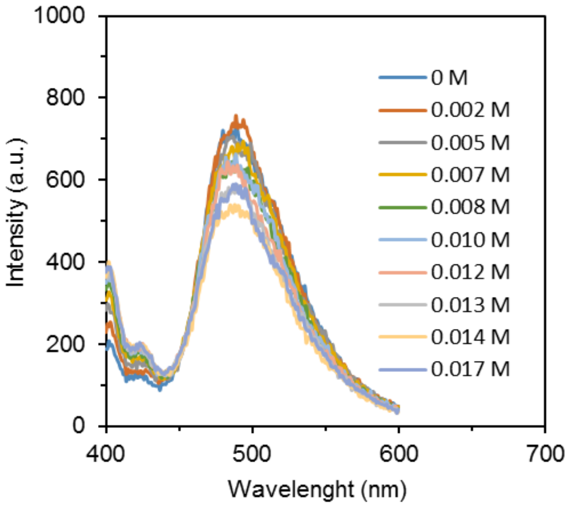

Figure S34. Fluorescence emission spectra of **1-NH$_2$$^{4+}$** (0.2 mM) in acetonitrile and in the presence of increasing [Benzyl alcohol]. The excitation wavelength was 350 nm.

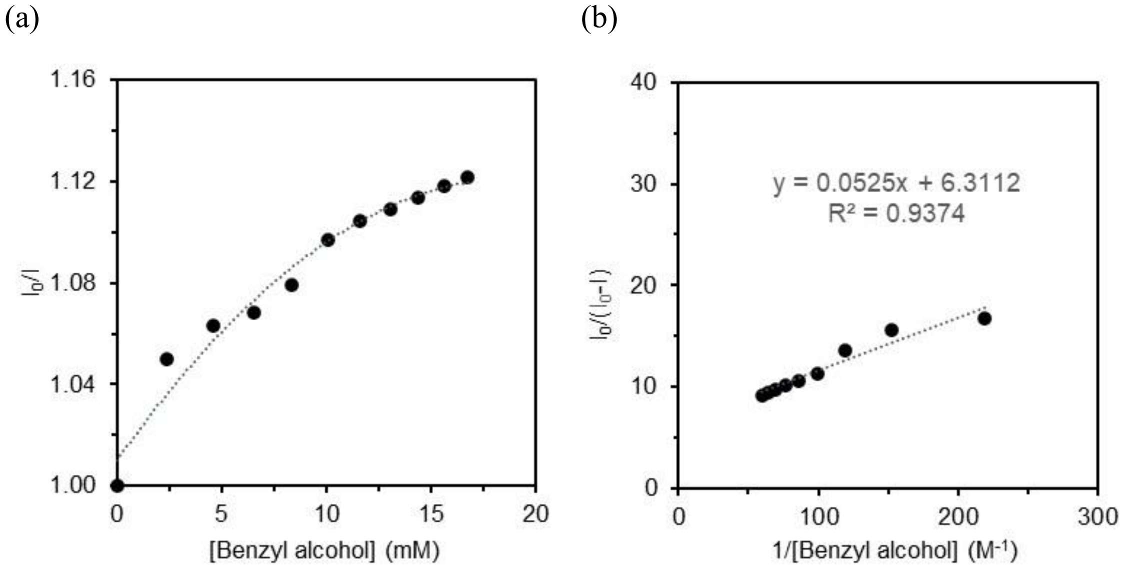

Figure S35. a) Stern-Volmer plot of I_0/I versus quencher concentration (Benzyl alcohol) under previous conditions. b) Lehrer plot of $I_0/(I_0\text{-}I)$ versus quencher concentration (Benzyl alcohol) under previous conditions.

S.9.3.3. Benzyl alcohol in presence 10 equiv. TBD.

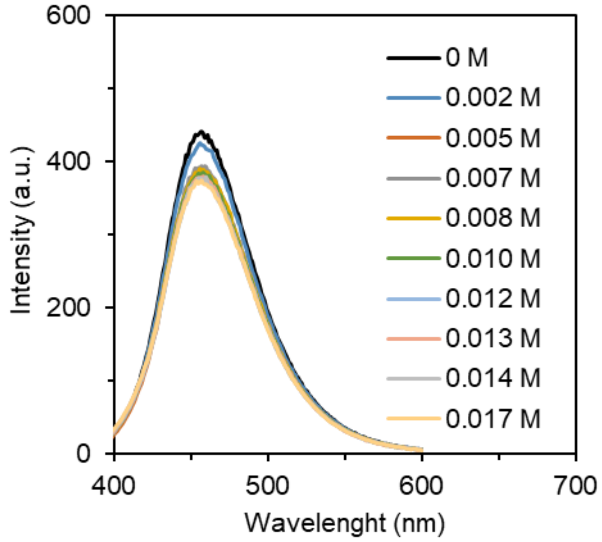

Figure S36. Fluorescence emission spectra of **1-NH$_2$$^{4+}$** (0.2 mM) in acetonitrile and in the presence of increasing [Benzyl alcohol]. The excitation wavelength was 350 nm.

(a) (b)

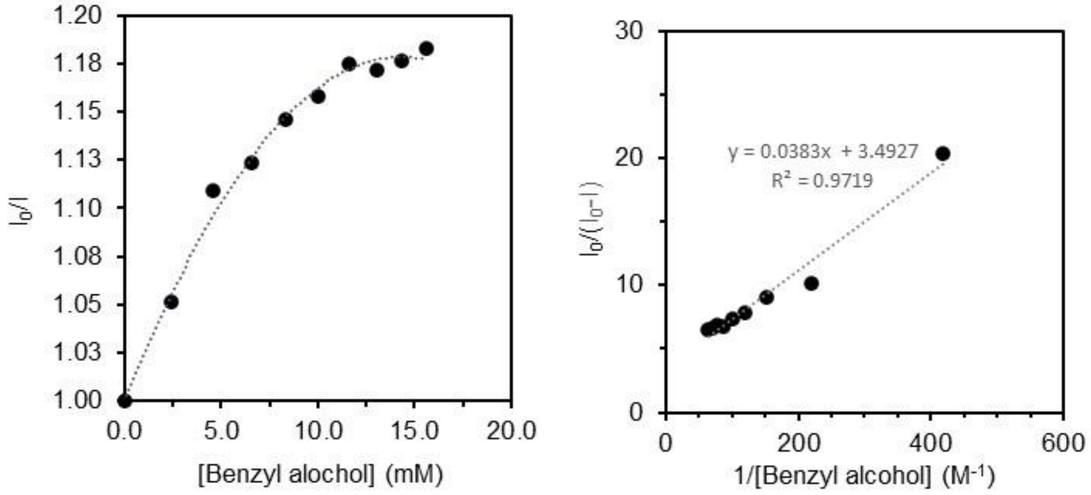

Figure S37. a) Stern-Volmer plot of I_0/I versus quencher concentration (Benzyl alcohol) under previous conditions. b) Lehrer plot of $I_0/(I_0\text{-}I)$ versus quencher concentration (Benzyl alcohol) under previous conditions.

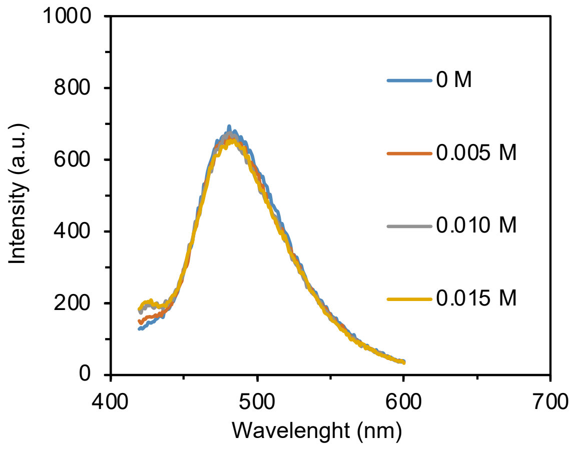

Figure S38. Fluorescence emission spectra of **1-NH$_2^{4+}$** (0.2 mM) in acetonitrile with Trimethylpyridine as the base, and in the presence of increasing [Benzyl alcohol]. The excitation wavelength was 350 nm. Due to the low pK_a of the Trimethylpyridine, uncapable to deprotonate **1-NH$_2^{4+}$**, no quenching was observed.

S.9.4. Other substrates.

S.9.4.1. O-H Bond.

S.9.4.1.1. Isopropanol with NEt_3.

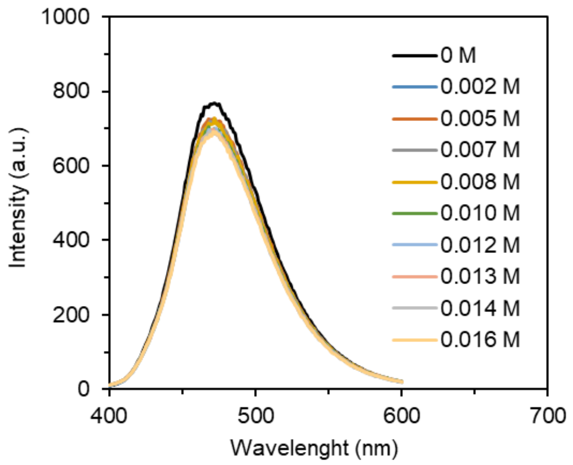

Figure S39. Fluorescence emission spectra of **1-NH$_2$$^{4+}$** (0.2 mM) in acetonitrile and in the presence of increasing [Isopropanol]. The excitation wavelength was 350 nm.

(a) (b)

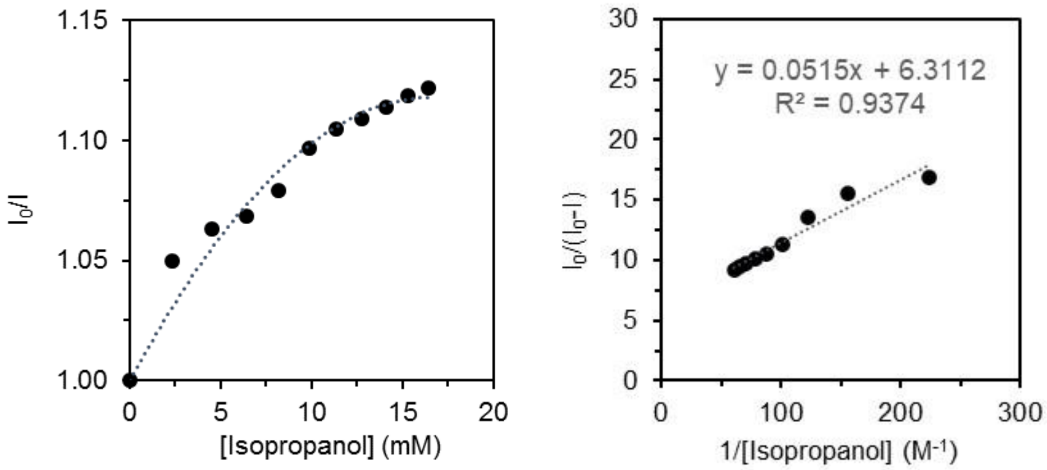

Figure S40. a) Stern-Volmer plot of I_0/I versus quencher concentration (Isopropanol) under previous conditions. b) Lehrer plot of $I_0/(I_0\text{-}I)$ versus quencher concentration (Isopropanol) under previous conditions.

S.9.4.1.2. Isopropanol without NEt_3.

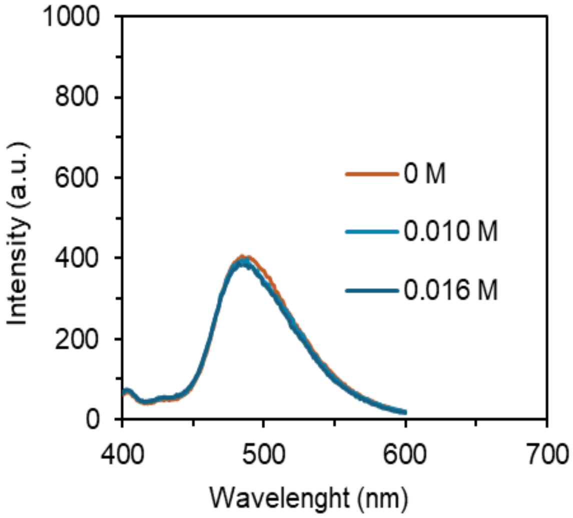

Figure S41. Fluorescence emission spectra of **1-NH$_2$$^{4+}$** (0.2 mM) in acetonitrile and in the presence of increasing [Isopropanol]. The excitation wavelength was 350 nm.

S.9.4.1.3. 4-Methoxybenzyl alcohol with NEt_3.

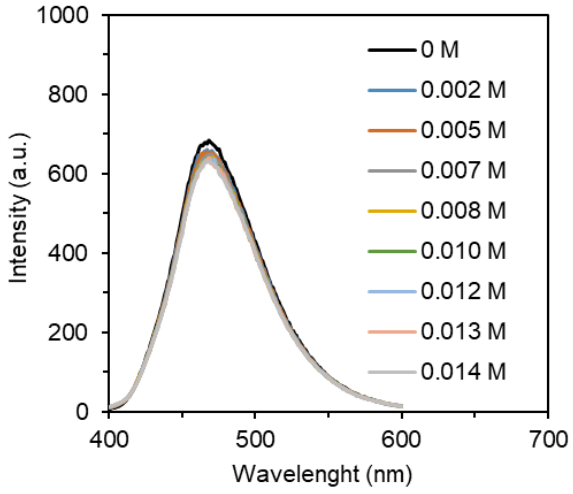

Figure S42. Fluorescence emission spectra of **1-NH$_2$$^{4+}$** (0.2 mM) in acetonitrile and in the presence of increasing [4-Methoxybenzyl alcohol]. The excitation wavelength was 350 nm.

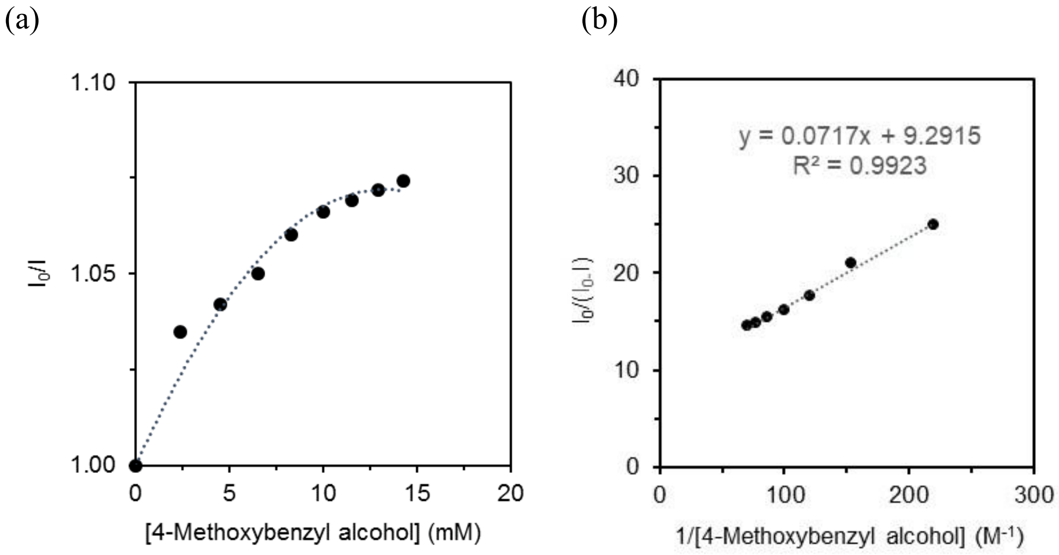

Figure S43. a) Stern-Volmer plot of I_0/I versus quencher concentration (4-Methoxybenzyl alcohol) under previous conditions. b) Lehrer plot of $I_0/(I_0-I)$ versus quencher concentration (4-Methoxybenzyl alcohol) under previous conditions.

S.9.4.1.4. 1-Phenylethanol and substituted analogues with NEt_3.

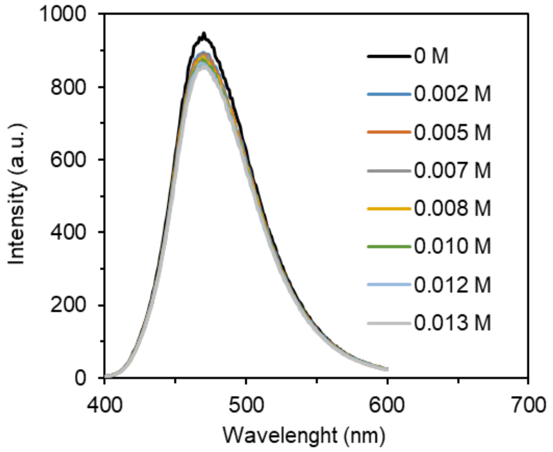

Figure S44. Fluorescence emission spectra of **1-NH$_2$$^{4+}$** (0.2 mM) in acetonitrile and in the presence of increasing [1-Phenylethanol]. The excitation wavelength was 350 nm.

(a) (b)

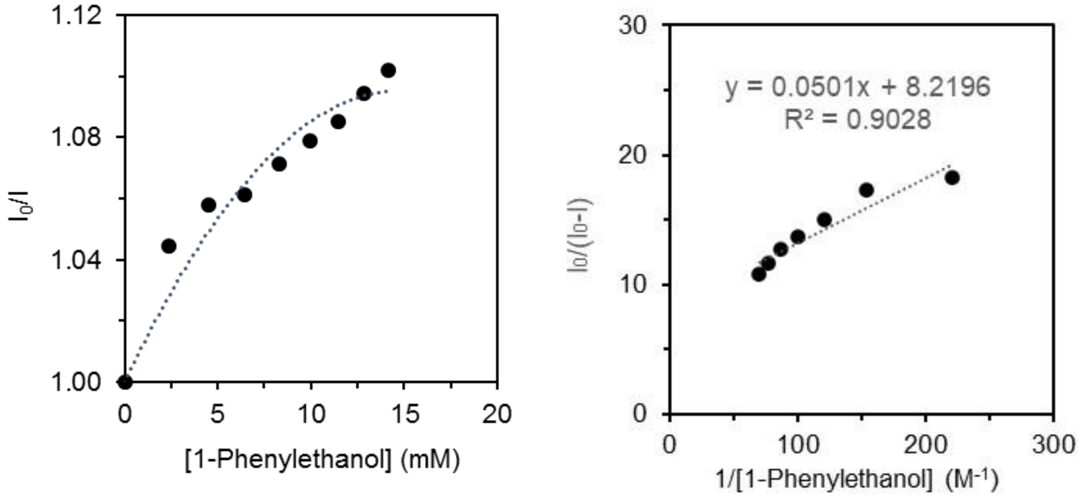

Figure S45. a) Stern-Volmer plot of I_0/I versus quencher concentration (1-Phenylethanol) under previous conditions. b) Lehrer plot of $I_0/(I_0-I)$ versus quencher concentration (1-Phenylethanol) under previous conditions.

(a) (b)

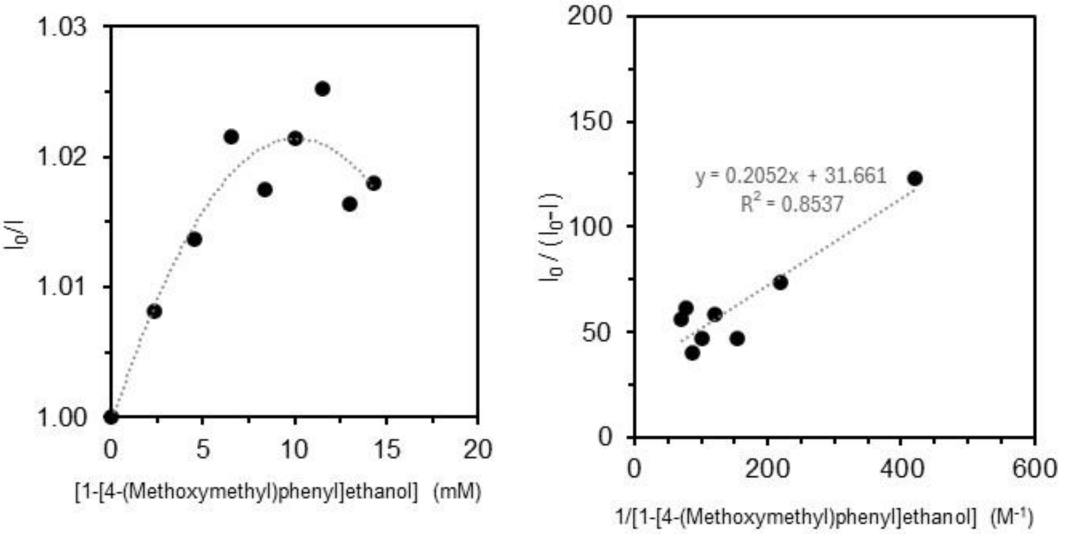

Figure S46. a) Stern-Volmer plot of I_0/I versus quencher concentration (1-(4-Methoxymethylphenyl)ethanol) under previous conditions. b) Lehrer plot of $I_0/(I_0\text{-}I)$ versus quencher concentration (1-(4-Methoxymethylphenyl)ethanol) under previous conditions.

(a) (b)

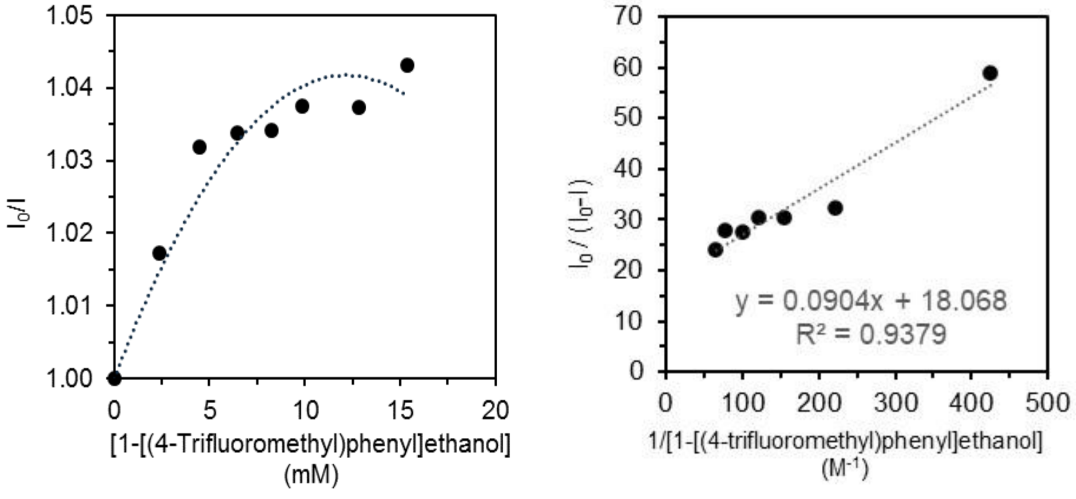

Figure S47. a) Stern-Volmer plot of I_0/I versus quencher concentration (1-[4-(Trifluoromethyl)phenyl]ethanol) under previous conditions. b) Lehrer plot of $I_0/(I_0\text{-}I)$ versus quencher concentration (1-[4-(Trifluoromethyl)phenyl]ethanol) under previous conditions.

S.9.4.2. C-H Bond.

S.9.4.2.1. 1,3-cyclohexadiene with NEt_3.

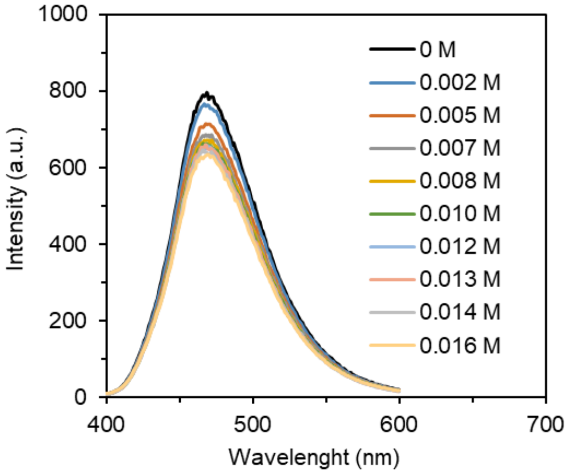

Figure S48. Fluorescence emission spectra of **1-NH$_2^{4+}$** (0.2 mM) in acetonitrile and in the presence of increasing [1,3-cyclohexadiene]. The excitation wavelength was 350 nm.

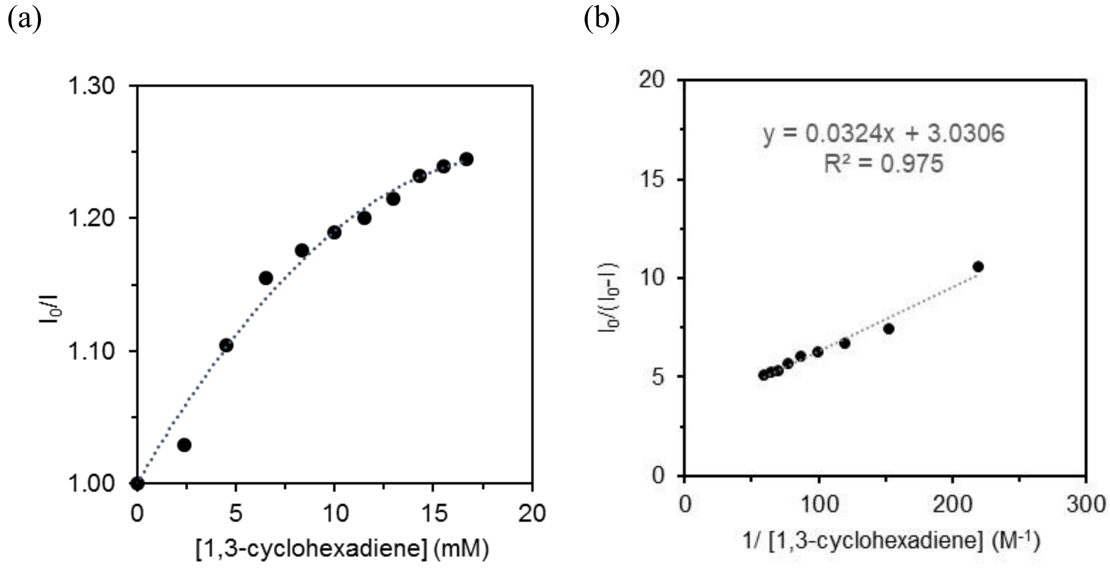

Figure S49. a) Stern-Volmer plot of I_0/I versus quencher concentration (1,3-cyclohexadiene) under previous conditions. b) Lehrer plot of $I_0/(I_0\text{-}I)$ versus quencher concentration (1,3-cyclohexadiene) under previous conditions.

S.9.4.2.2. 9,10-Dihydroanthracene with NEt_3.

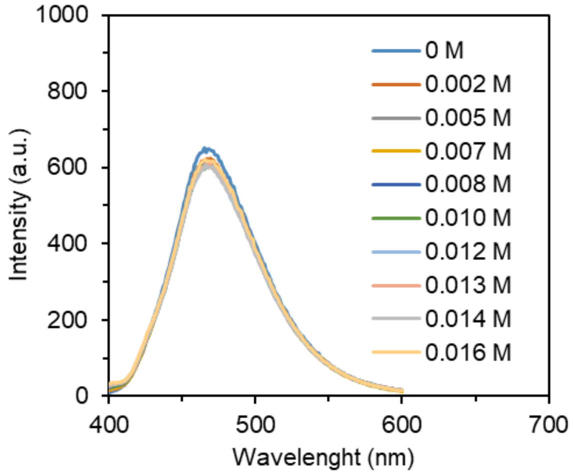

Figure S50. Fluorescence emission spectra of **1-NH_2^{4+}** (0.2 mM) in acetonitrile and in the presence of increasing [9,10-Dihydroanthracene]. The excitation wavelength was 350 nm.

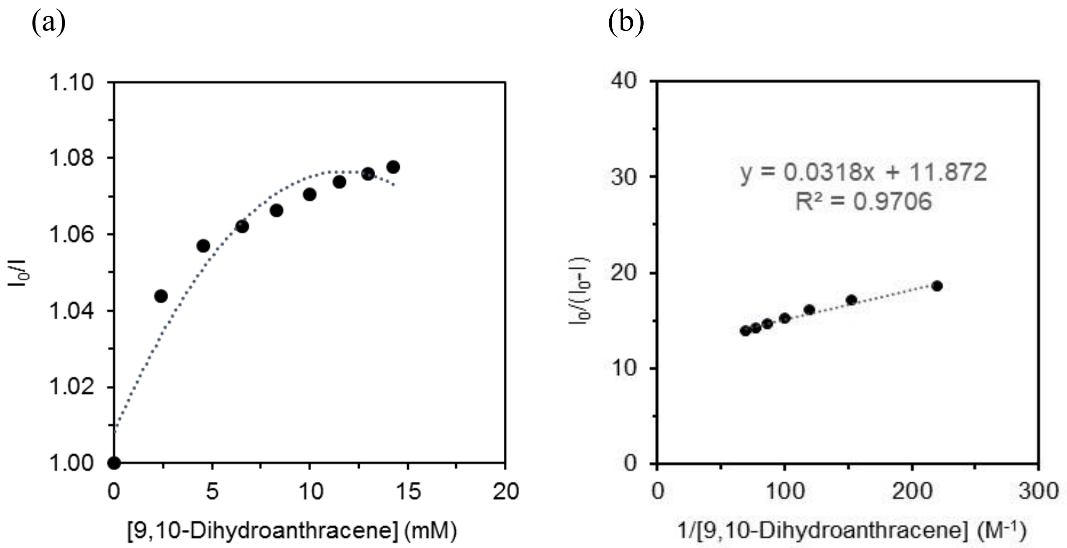

Figure S51. a) Stern-Volmer plot of I_0/I versus quencher concentration (9,10-Dihydroanthracene) under previous conditions. b) Lehrer plot of $I_0/(I_0-I)$ versus quencher concentration (9,10-Dihydroanthracene) under previous conditions.

S.9.4.2.3. 9,10-Dihydroanthracene without NEt_3.

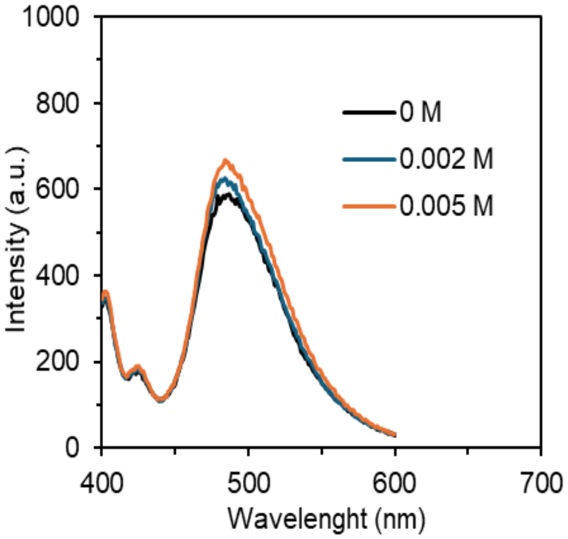

Figure S52. Fluorescence emission spectra of **1-NH_2^{4+}** (0.2 mM) in acetonitrile and in the presence of increasing [9,10-Dihydroanthracene]. The excitation wavelength was 350 nm.

S.9.4.2.4. Diphenylmethane with NEt_3.

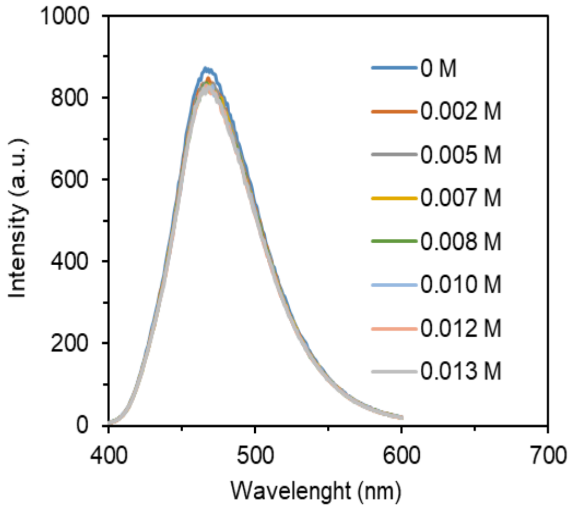

Figure S53. Fluorescence emission spectra of **1-NH_2^{4+}** (0.2 mM) in acetonitrile and in the presence of increasing [Diphenylmethane]. The excitation wavelength was 350 nm.

(a) (b)

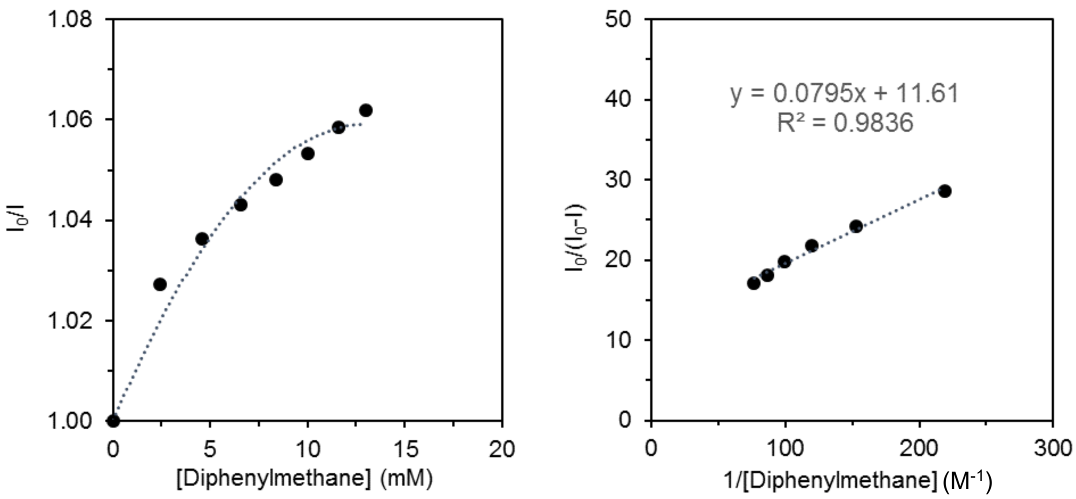

Figure S54. a) Stern-Volmer plot of I_0/I versus quencher concentration (Diphenylmethane) under previous conditions. b) Lehrer plot of $I_0/(I_0\text{-}I)$ versus quencher concentration (Diphenylmethane) under previous conditions.

S.9.4.2.5. Fluorene with NEt_3.

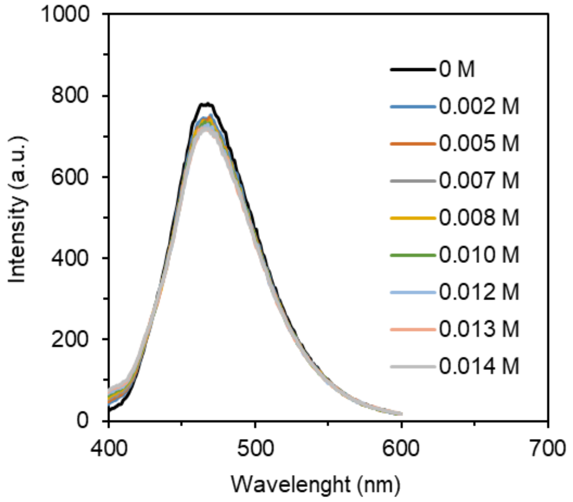

Figure S55. Fluorescence emission spectra of **1-NH_2^{4+}** (0.2 mM) in acetonitrile and in the presence of increasing [Fluorene]. The excitation wavelength was 350 nm.

(a) (b)

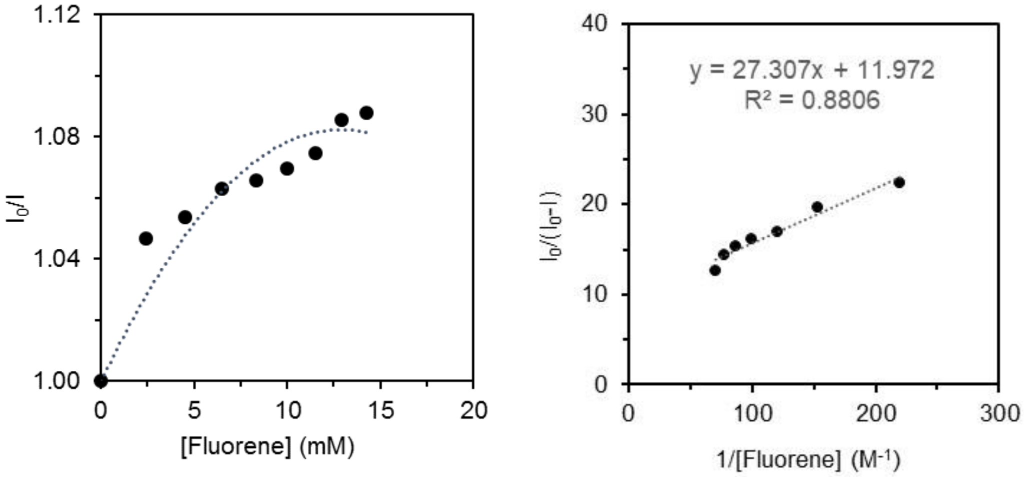

Figure S56. a) Stern-Volmer plot of I_0/I versus quencher concentration (Fluorene) under previous conditions. b) Lehrer plot of $I_0/(I_0\text{-}I)$ versus quencher concentration (Fluorene) under previous conditions.

S.9.5. MeOH in acetonitrile.

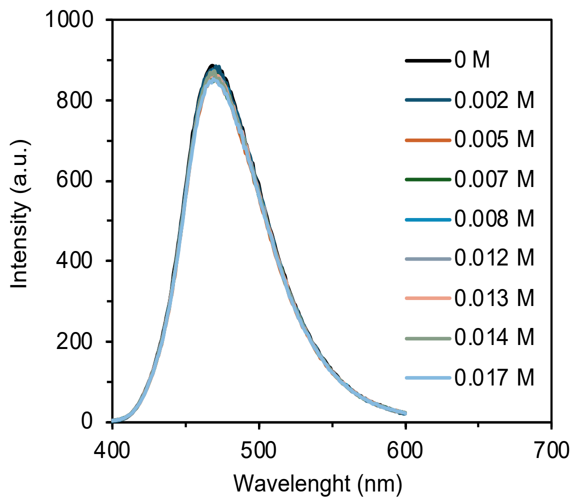

Figure S57. Fluorescence emission spectra of **1-NH$_2$$^{4+}$** (0.2 mM) in acetonitrile and in the presence of increasing [MeOH]. The excitation wavelength was 350 nm.

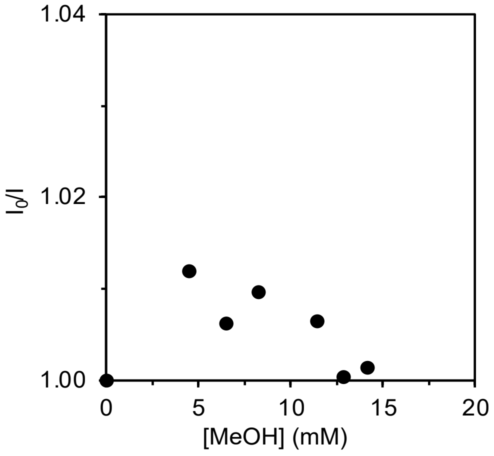

Figure S58. Stern-Volmer plot of I_0/I versus quencher concentration (MeOH) under previous conditions, showing the lack of effective quenching.

S.9.6. 9,10-Dihydroanthracene in MeOH.

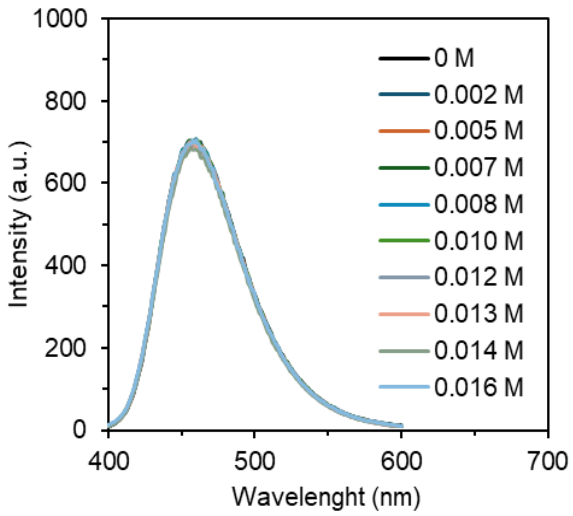

Figure S59. Fluorescence emission spectra of **1-NH$_2^{4+}$** (0.2 mM) in MeOH and in the presence of increasing [9,10-Dihydroanthracene]. The excitation wavelength was 350 nm. This experiment demonstrates the lack of quenching with 9,10-Dihydroanthracene in methanol.

S.9.7. Benzyl alcohol in MeOH.

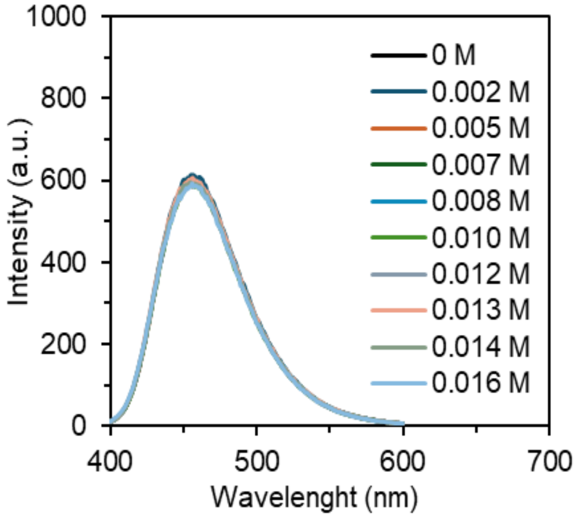

Figure S60. Fluorescence emission spectra of **1-NH$_2^{4+}$** (0.2 mM) in MeOH and in the presence of increasing [Benzyl alcohol]. The excitation wavelength was 350 nm. This experiment demonstrates the lack of quenching with Benzyl alcohol in methanol.

S.9.8. $Na_2S_2O_8$.

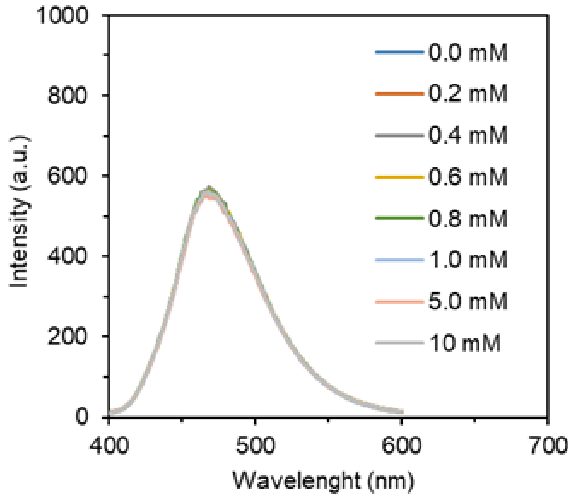

Figure S61. Fluorescence emission spectra of **1-NH$_2^{4+}$** (0.2 mM) in acetonitrile with added NEt_3 and in the presence of increasing [$Na_2S_2O_8$]. The excitation wavelength was 350 nm. This experiment demonstrates the lack of quenching with $Na_2S_2O_8$ in MeCN. Note: $Na_2S_2O_8$ might not be fully soluble at concentrations above 1 mM.

(a) (b)

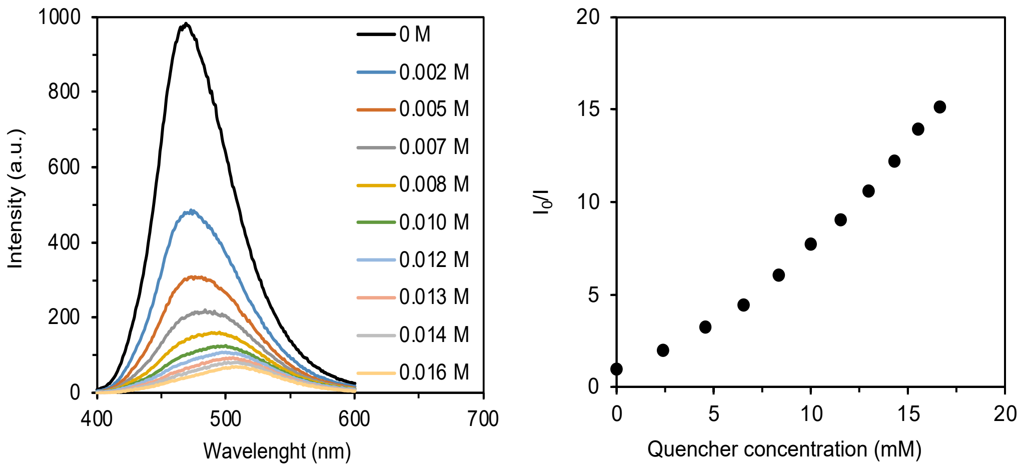

Figure S62. (a) Fluorescence emission spectra of **1-NH$_2^{4+}$** (0.2 mM) in acetonitrile and in the presence of increasing [Ferrocene]. The excitation wavelength was 350 nm. This experiment demonstrates the lack of quenching with $Na_2S_2O_8$ in acetonitrile. (b) Stern-Volmer analysis of the quenching data.

S.10. Photocatalytic reaction.

General protocol:

The photocatalytic reactions were carried out under nitrogen atmosphere. In a 5 mL glass vial **1-NH$_2^{4+}$** (1 µmol), substrate (50 µmol), sacrificial electron acceptor (1000 µmol) and solvent (2 mL) were added depending on the specific conditions. The mixture was degassed during 15 mins and irradiated with blue light (440 nm wavelength, 100 W KESSIL LIGHT LEDs) under stirring during 24h. At the end of the reaction, the solvent was evaporated, and the solid powder was dissolved in 600 µL of chloroform-d with an internal standard, dried over sodium sulphate and analysed by ^{1}H-NMR.

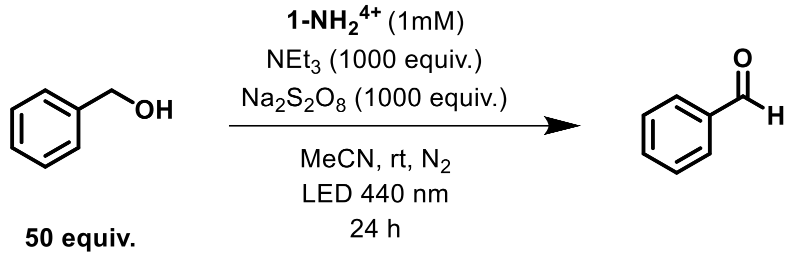

Figure S63. Scheme of photocatalytic reaction with Benzyl alcohol.

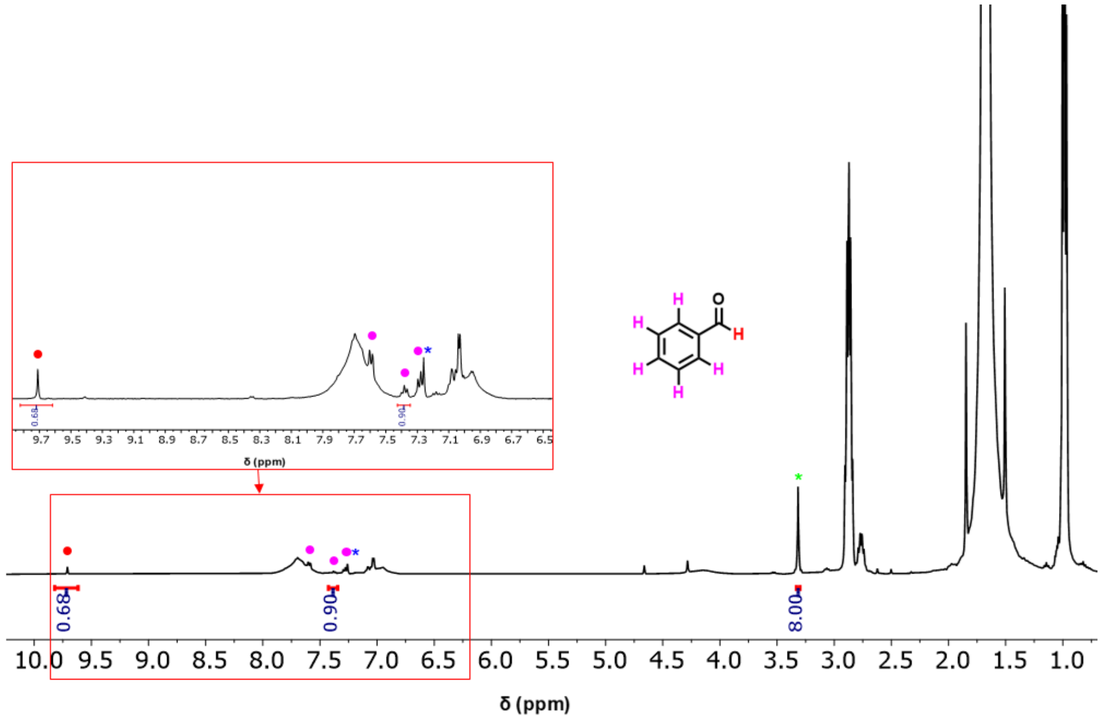

Figure S64. ^{1}H-NMR spectra of the products from the photochemical oxidation of Benzyl alcohol in acetonitrile, showing the Benzaldehyde signals (red and pink). Solvent signals are marked with blue asterisk (chloroform-d) and internal standard as green asterisk (1,4-Dioxane) added after photoreaction. Coincident with reported spectrum.[13,14]

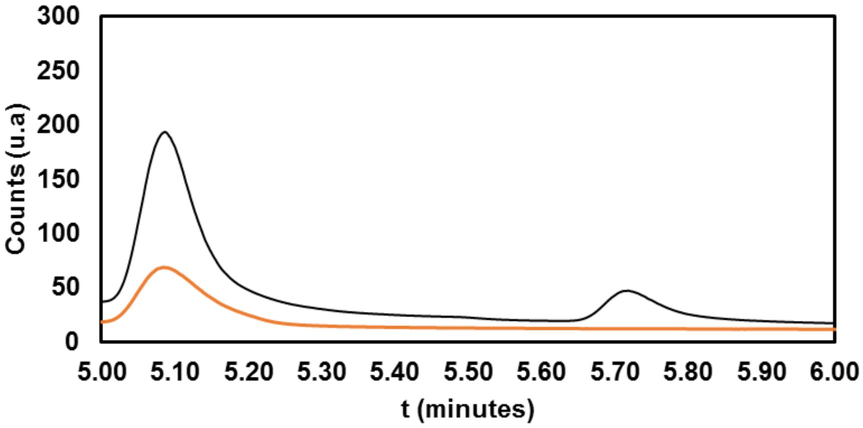

Figure S65. GC spectra of the products from the photochemical oxidation of Benzyl alcohol in acetonitrile (black trace), showing the Benzaldehyde signals as compared to a standard sample (orange trace) at 5.08 minutes and Benzyl alcohol at 5.78 minutes.

S.10.1. Calibration Curve.

S.10.1.1. Calibration Curve in methanol.

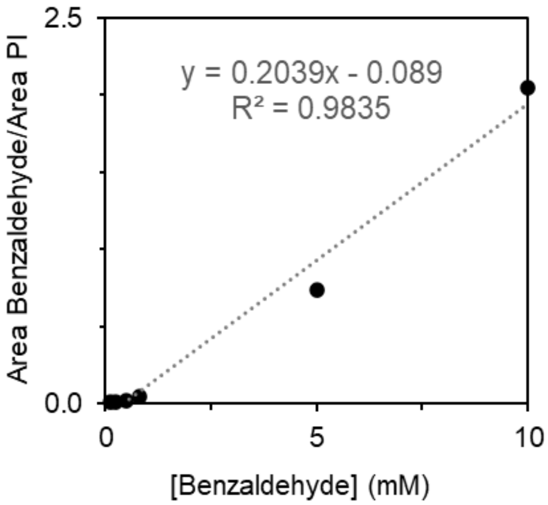

Figure S66. Calibration curve of Benzaldehyde on the GC-FID using Toluene as internal standard in methanol.

S.10.1.2. Calibration Curve in acetonitrile.

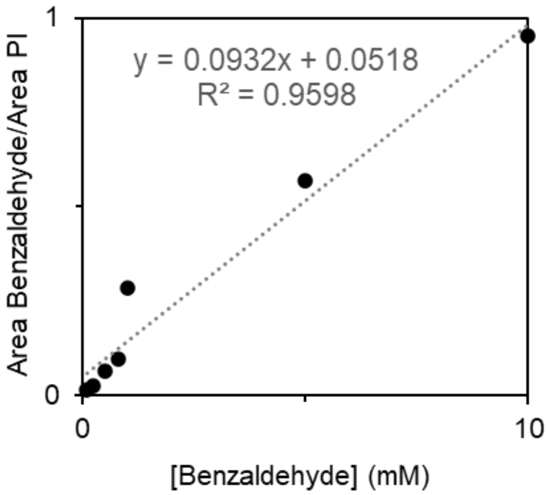

Figure S67. Calibration curve of Benzaldehyde on the GC-FID using Toluene as internal standard in acetonitrile.

S.10.1.3. Calibration Curve in isopropanol.

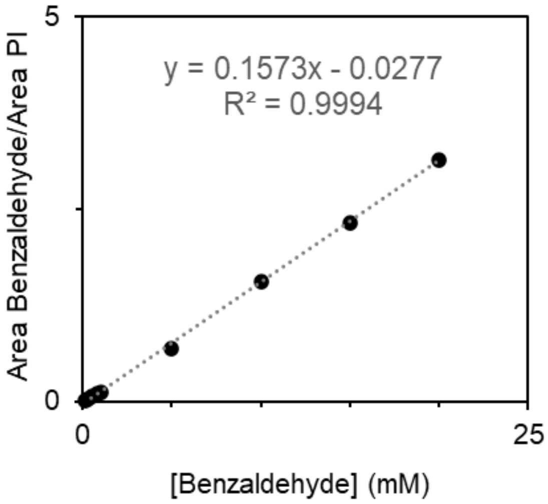

Figure S68. Calibration curve of Benzaldehyde on the GC-FID using Toluene as internal standard in isopropanol.

S.10.1.4. Calibration Curve in chloroform.

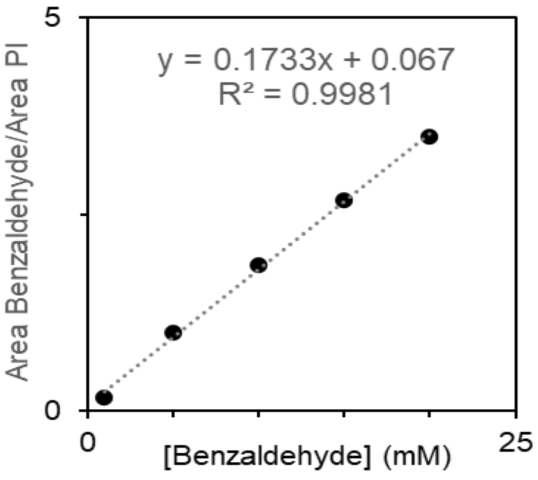

Figure S69. Calibration curve of Benzaldehyde on the GC-FID using Toluene as internal standard in chloroform.

S.10.2. Study conditions.

S.10.2.1. Effect presence of H_2O and O_2 with $Na_2S_2O_8$ as SEA.

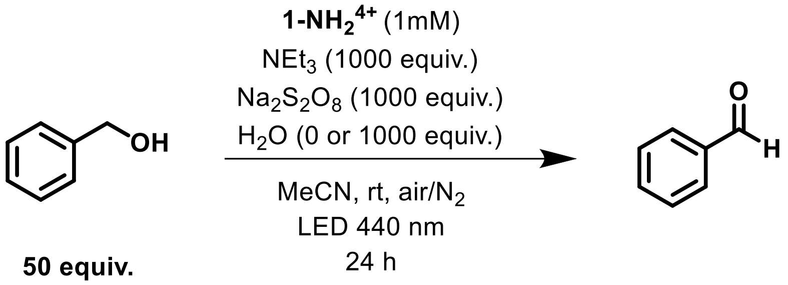

Figure S70. Scheme of the photocatalytic reaction.

Table S1. Yield and turnover number obtained in the photocatalytic reaction studying the effect of H_2O and O_2 with $Na_2S_2O_8$ as SEA.

ACID	ATMOSPHERE	[Benzaldehyde] (mM)	YIELD (%)	TURNOVER
-	N_2	21.3	42.6	21
H_2O	N_2	0.3	0.6	1
-	Air (with O_2)	13.6	27.2	14

S.10.2.2. Effect Solvent.

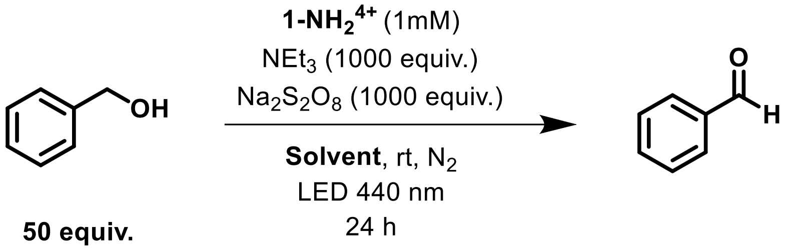

Figure S71. Scheme of photocatalytic reaction mediated by **1-NH_2^{4+}** with different solvents.

Table S2. Yield and turnover number obtained in the photocatalytic reaction studying the effect of the solvent.

SOLVENT	[Benzaldehyde] (mM)	YIELD (%)	TURNOVER
Methanol	11.7	23.4	12
Isopropanol	10.0	20.0	10
Chloroform	3.1	6.2	3.1

S.10.2.3. Effect equivalents of substrate.

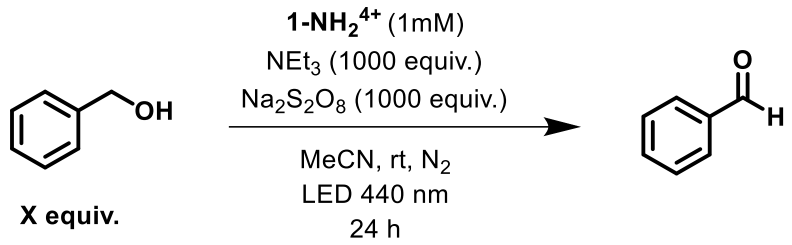

Figure S72. Scheme of photocatalytic reaction with different equivalents of substrate.

Table S3. Yield and turnover number obtained in the photocatalytic reaction studying the effect of the substrate equivalents.

EQUIVALENTS	[Benzaldehyde] (mM)	YIELD (%)	TURNOVER
100	33.5	33.5	34
200	64.6	32.3	65
500	90.3	18.1	90

S.10.3. Other substrates.

S.10.3.1. 4-Methoxybenzyl alcohol.

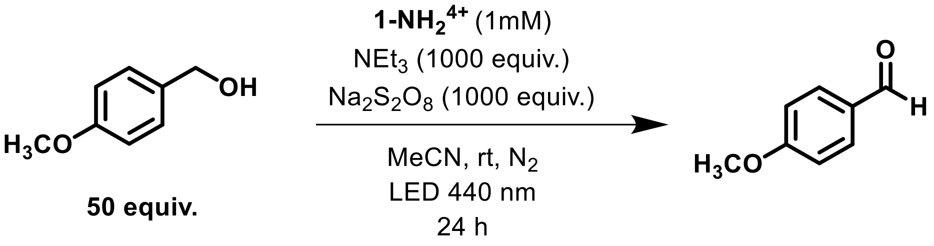

Figure S73. Scheme of photocatalytic reaction.

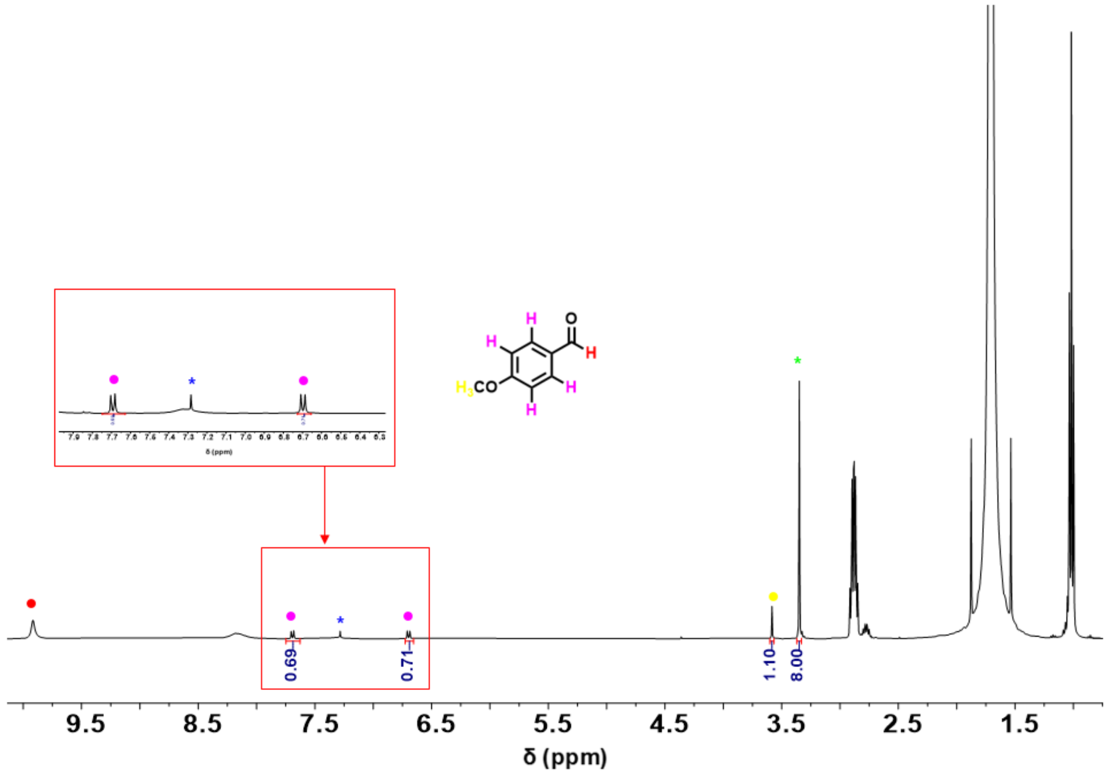

Figure S74. ^{1}H-NMR spectra of the products from the photochemical oxidation of 4-Methoxybenzyl alcohol in acetonitrile, showing the 4-Methoxybenzaldehyde signals (red, pink and yellow). Solvent signals are marked with blue asterisk (chloroform-d) and internal standard as green asterisk (1,4-Dioxane) added after photoreaction. Coincident with reported spectrum.[13,14]

S.10.3.2. 1-Phenylethanol.

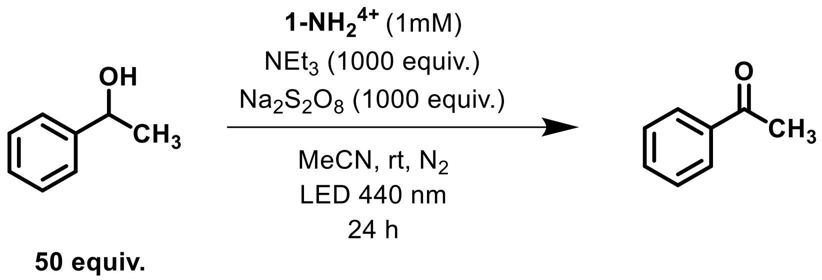

Figure S75. Scheme of photocatalytic reaction.

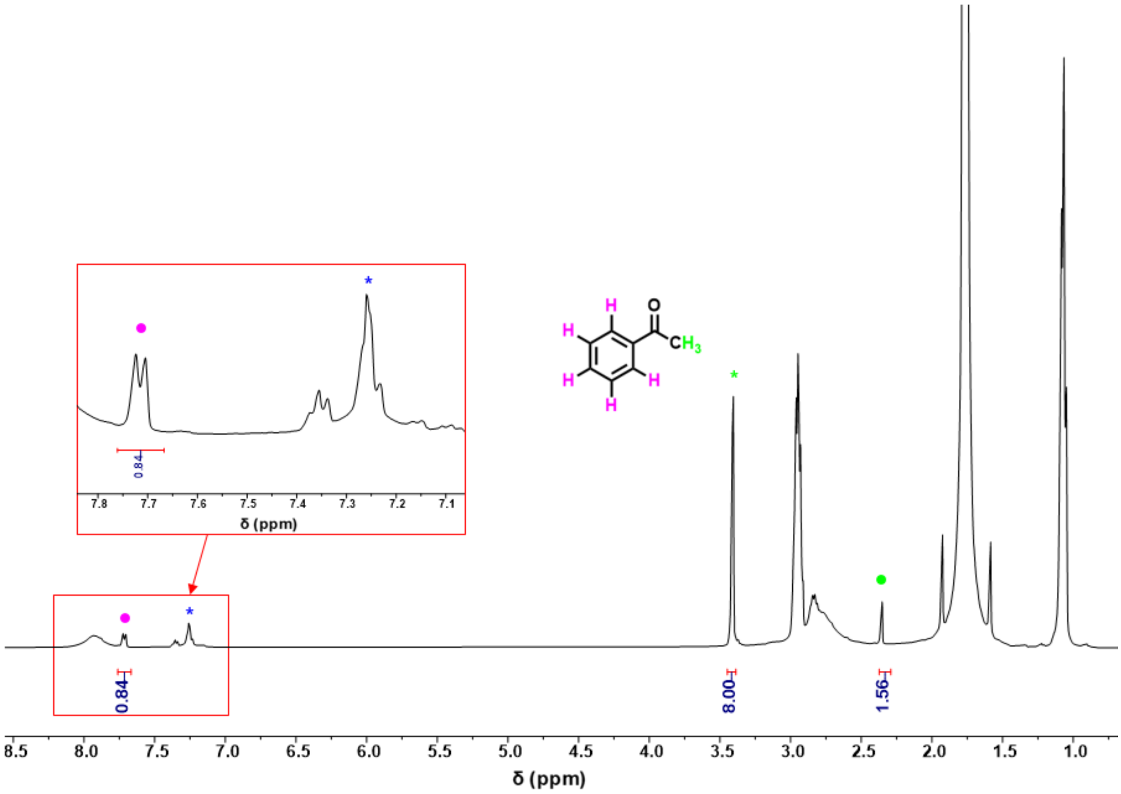

Figure S76. ^{1}H-NMR spectra of the products from the photochemical oxidation of 1-Phenylethanol in acetonitrile, showing the Acetophenone signals (pink and green). Solvent signals are marked with blue asterisk (chloroform-d) and internal standard as green asterisk (1,4-Dioxane) added after photoreaction. Coincident with reported spectrum.[15,16]

S.10.3.3. 1-(4-Methoxyphenyl)ethanol.

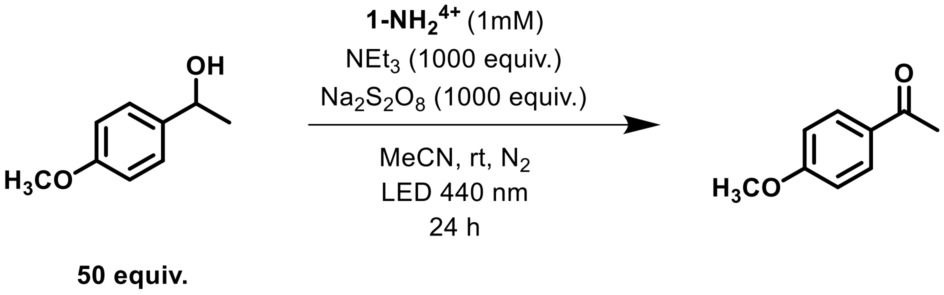

Figure S77. Scheme of photocatalytic reaction.

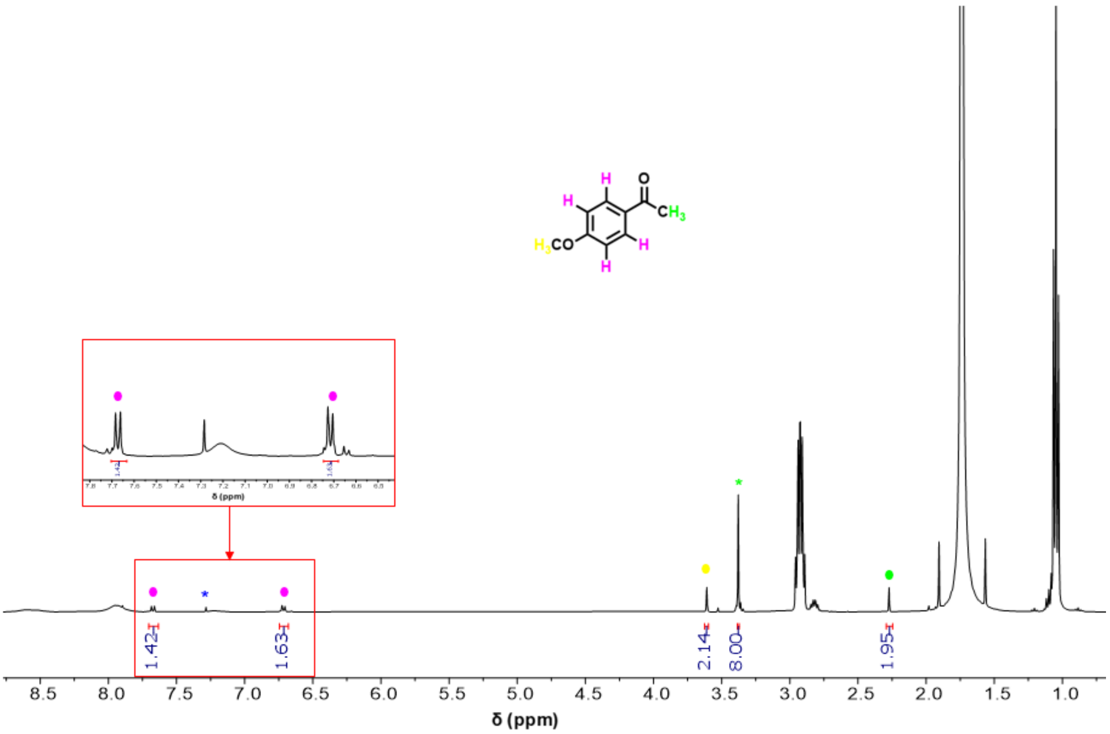

Figure S78. ^{1}H-NMR spectra of the products from the photochemical oxidation of 1-(4-Methoxyphenyl)ethanol in acetonitrile, showing the 1-(4-Methoxyphenyl)ethanone signals (pink, yellow and green). Solvent signals are marked with blue asterisk (chloroform-d) and internal standard as green asterisk (1,4-Dioxane) added after photoreaction. Coincident with reported spectrum.[17]

S.10.3.4. 1-[4-(Trifluoromethyl)phenyl]ethanol.

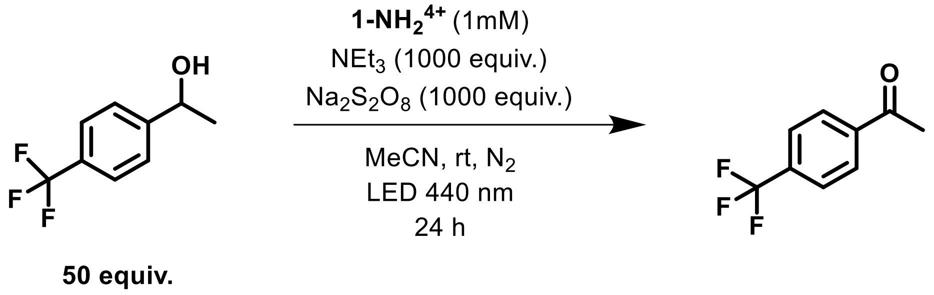

Figure S79. Scheme of photocatalytic reaction.

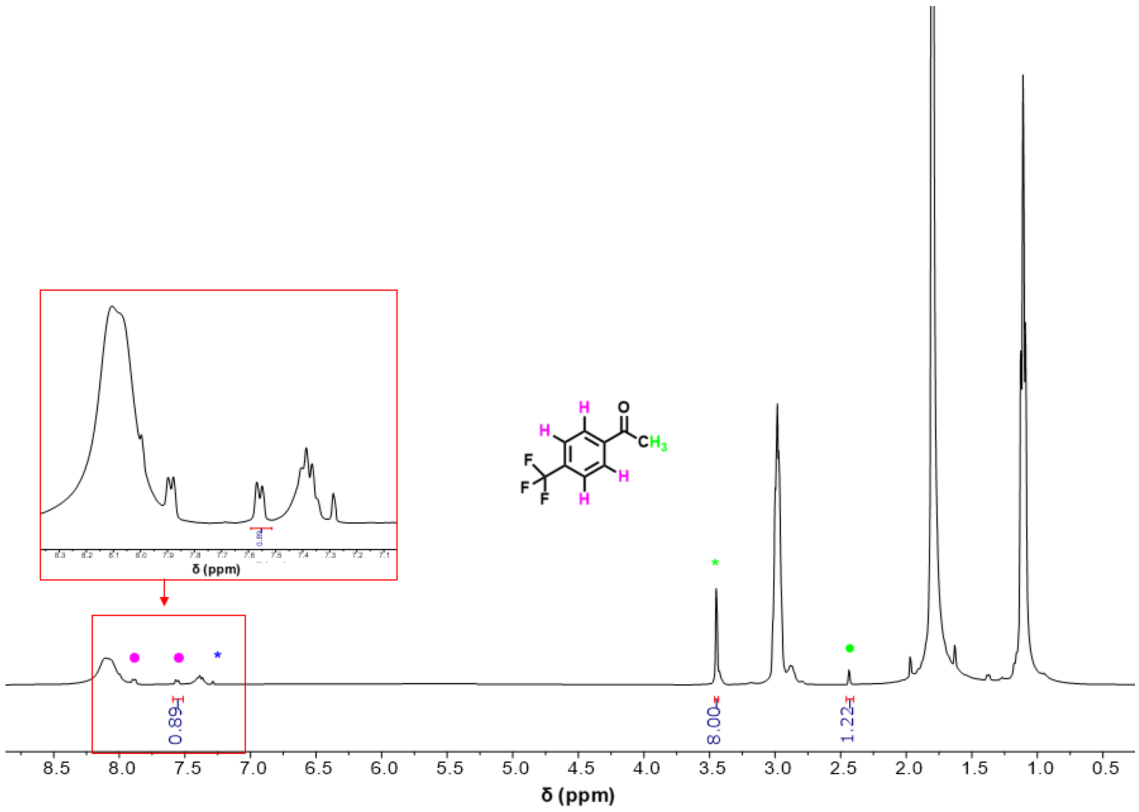

Figure S80. ^{1}H-NMR spectra of the products from the photochemical oxidation of 1-[4-(Trifluoromehtyl)phenyl]ethanol in acetonitrile, showing the 1-(4-(trifluoromethyl) phenyl)ethanone signals (pink and green). Solvent signals are marked with blue asterisk (chloroform-d) and internal standard as green asterisk (1,4-Dioxane) added after photoreaction. Coincident with reported spectrum.[18,19]

S.10.3.5. 1,3-cyclohexadiene.

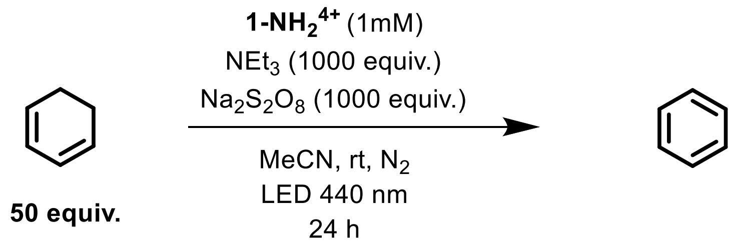

Figure S81. Scheme of photocatalytic reaction.

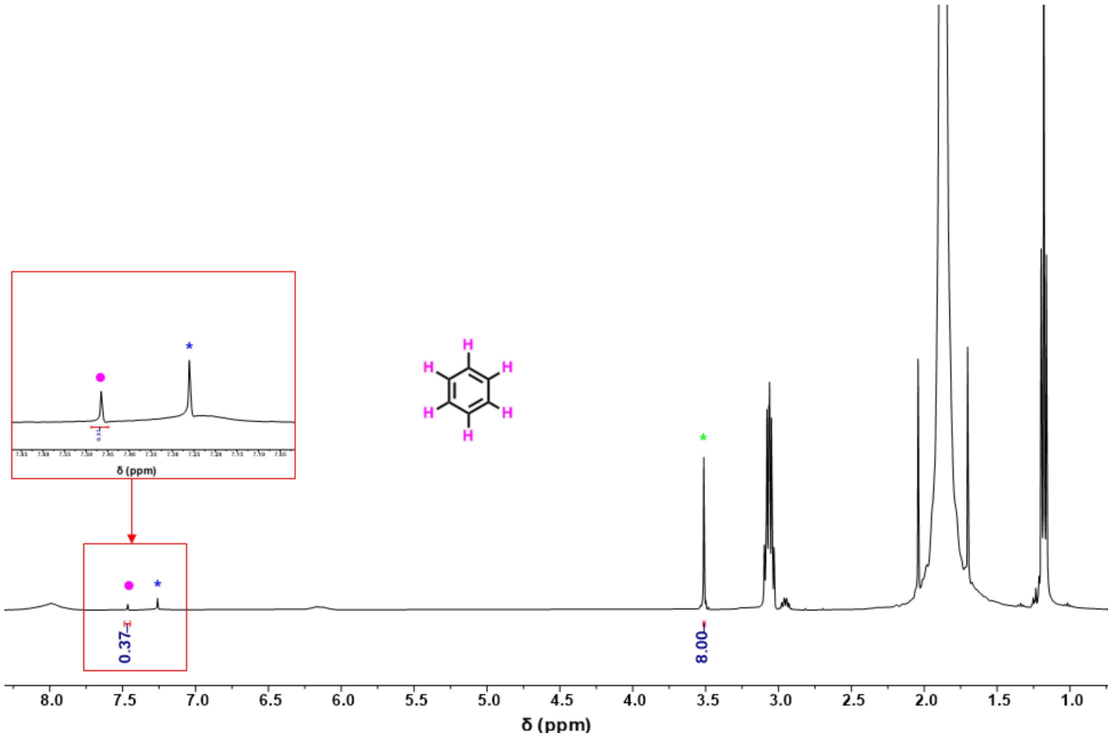

Figure S82. ^{1}H-NMR spectra of the products from the photochemical oxidation of 1,3-cyclohexadiene in acetonitrile, showing the Benzene signals (pink). Solvent signals are marked with blue asterisk (chloroform-d) and internal standard as green asterisk (1,4-Dioxane) added after photoreaction. Coincident with the chemical shift of Benzene. This experiment was also performed in acetonitrile-d3 for direct analysis by ^{1}H NMR to check if the work up method affected the quantification, but we obtained a similar value.

S.10.3.6. 9,10-Dihydroanthracene.

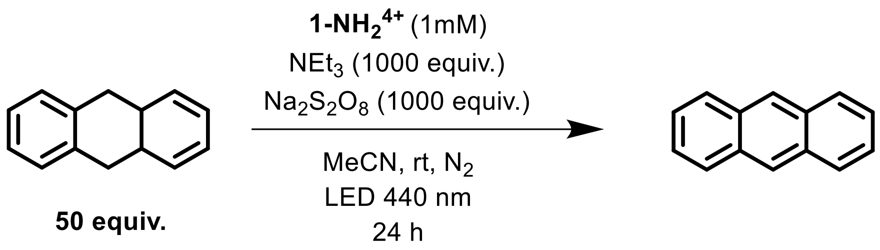

Figure S83. Scheme of photocatalytic reaction.

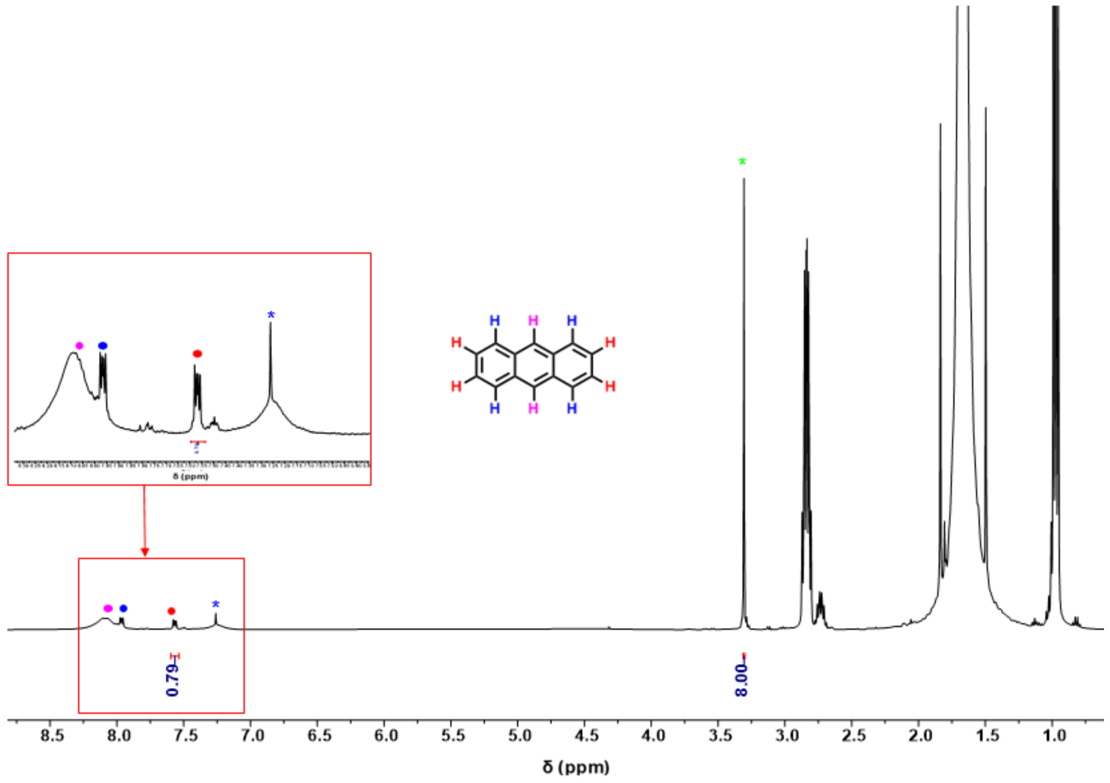

Figure S84. ^{1}H-NMR spectra of the products from the photochemical oxidation of 9,10-Dihydroanthracene in acetonitrile, showing the Anthracene signals (pink, blue and red). Solvent signals are marked with blue asterisk (chloroform-d) and internal standard as green asterisk (1,4-Dioxane) after photoreaction. Coincident with reported spectrum.[20] The protons at 9 and 10 position are below the broad signal at 8.2 ppm, so we confirmed the formation of Anthracene by GC-MS as shown below.

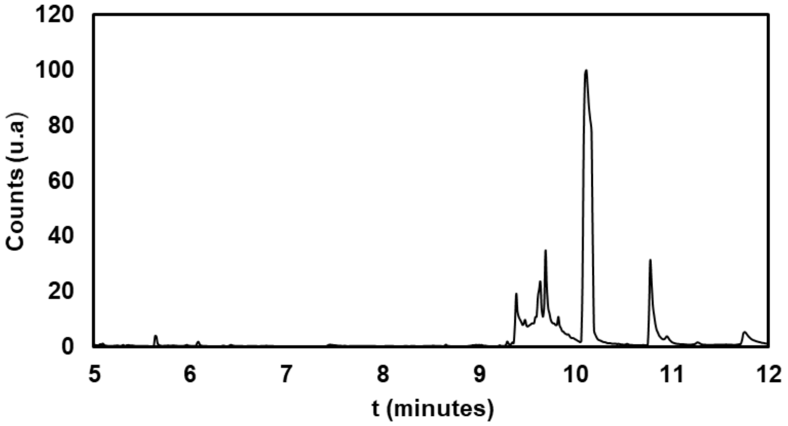

Figure S85. GC spectra of the products from the photochemical oxidation of 9,10-Dihydroanthracene (retention time: 10 min) to Anthracene (retention time: around 11 min) in acetonitrile.

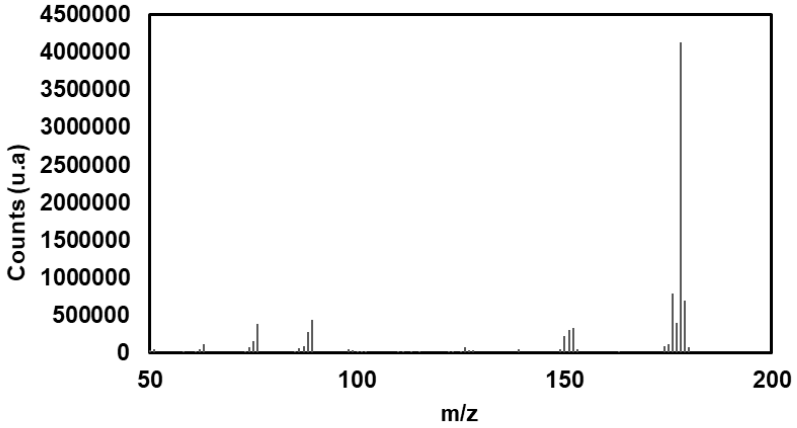

Figure S86. MS spectra of the product from the photochemical oxidation of 9,10-Dihydroanthracene in acetonitrile, showing the Anthracene signals corresponding to a retention time around 11 min.

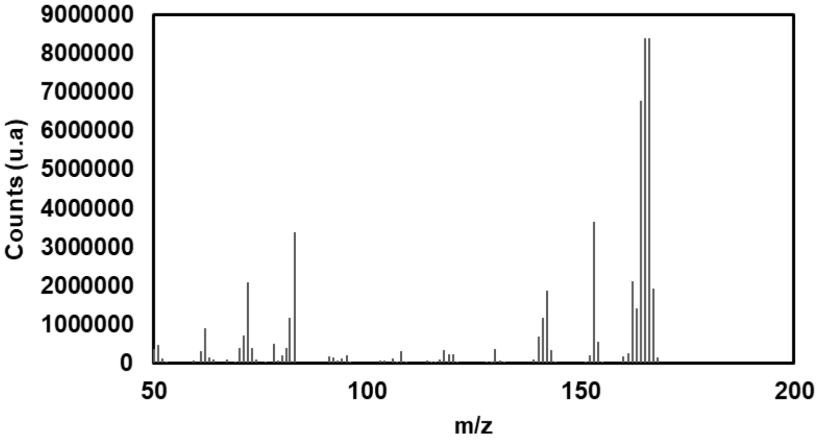

Figure S87. MS spectra of the substrate from the photochemical oxidation of 9,10-Dihydroanthracene in acetonitrile, showing the 9,10-Dihydroanthracene signals corresponding to a retention time around 10 min.

S.10.3.7. Diphenylmethane.

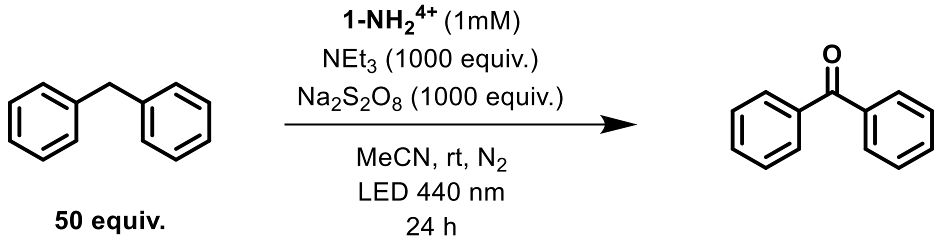

Figure S88. Scheme of photocatalytic reaction.

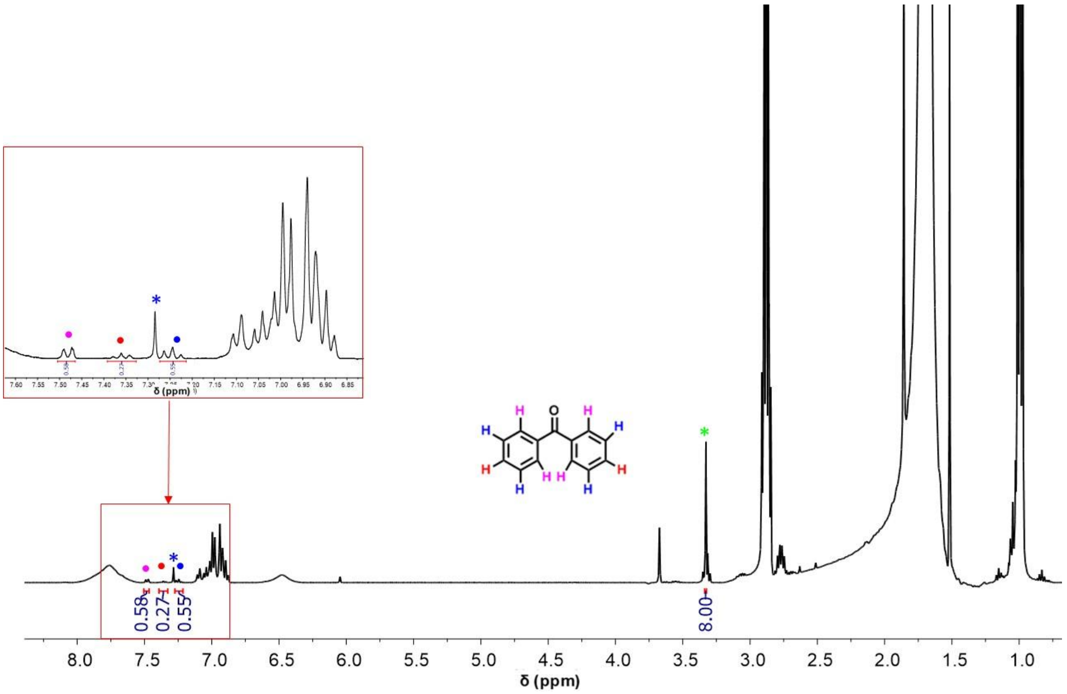

Figure S89. ^{1}H-NMR spectra of the products from the photochemical oxidation of Diphenylmethane in acetonitrile, showing the Benzophenone signals (pink, blue and red). Solvent signals are marked with blue asterisk (chloroform-d) and internal standard as green asterisk (1,4-Dioxane) added after photoreaction. Coincident with reported spectrum.[13, 17, 21]

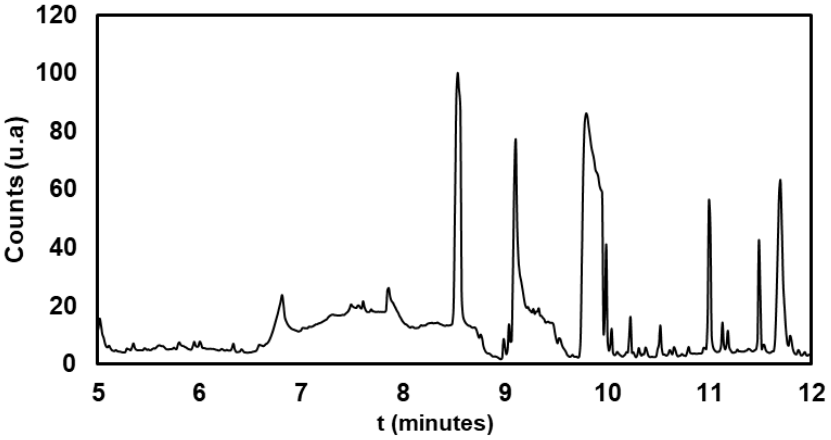

Figure S90. GC spectra of the products from the photochemical oxidation of Diphenylmethane (retention time: 8.5 min) to Benzophenone (retention time: 9.8) in acetonitrile.

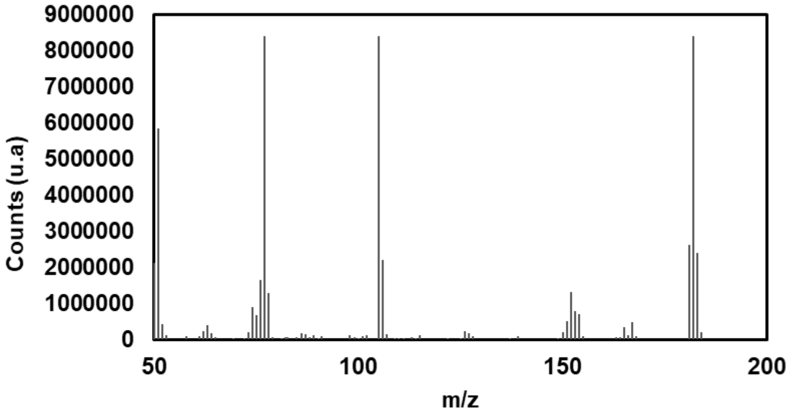

Figure S91. MS spectra of the product from the photochemical oxidation of Diphenylmethane in acetonitrile, showing the Benzophenone signals corresponding to a retention time around 9.8 min.

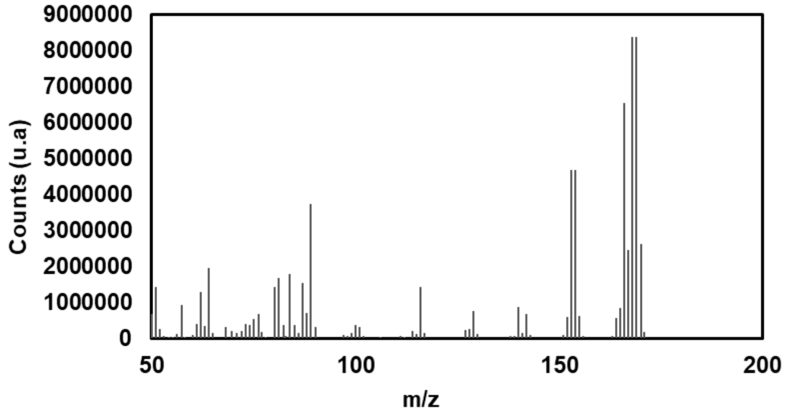

Figure S92. MS spectra of the substrate from the photochemical oxidation of Diphenylmethane in acetonitrile, showing the Diphenylmethane signals corresponding to a retention time around 8.5 min.

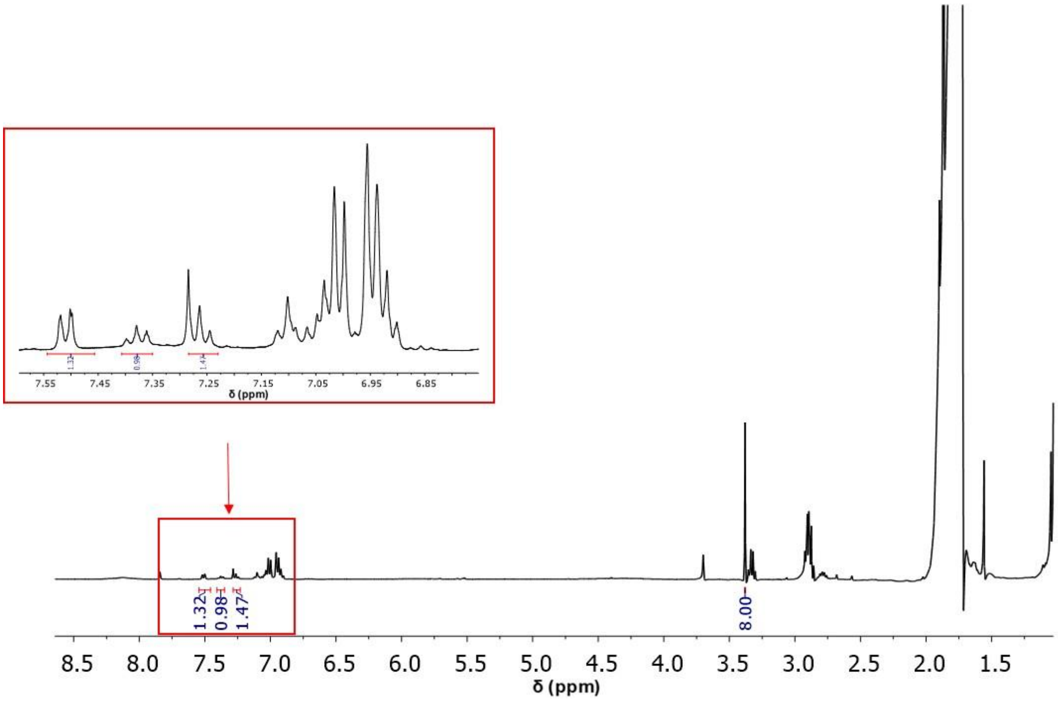

Figure S93.^{1}H-NMR spectra of the products from the photochemical oxidation of Diphenylmethane in acetonitrile with added NaOH 0.1 M in water (200 µL; final NaOH concentration of 0.01 mM), showing the Benzophenone signals. In this experiment, 16.5 equiv. of Benzophenone were produced.

Table S4. Yield and equivalents obtained in the photocatalytic reaction studying the effect of H_2O and O_2 with $Na_2S_2O_8$ as SEA.

ACID	ATMOSPHERE	YIELD (%)	Equiv.
-	N_2	16	8
H_2O	N_2	3	1.5
-	Air (with O_2)	n.d.	n.d.

S.10.3.8. Fluorene.

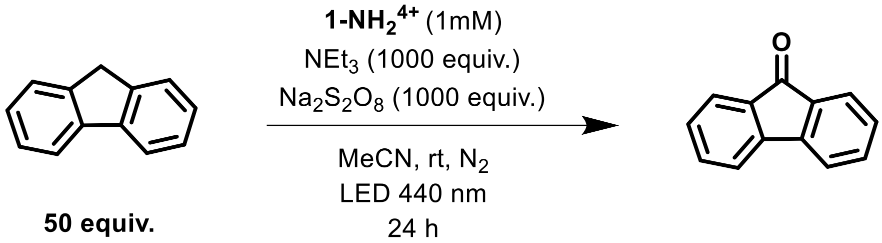

Figure S94. Scheme of photocatalytic reaction.

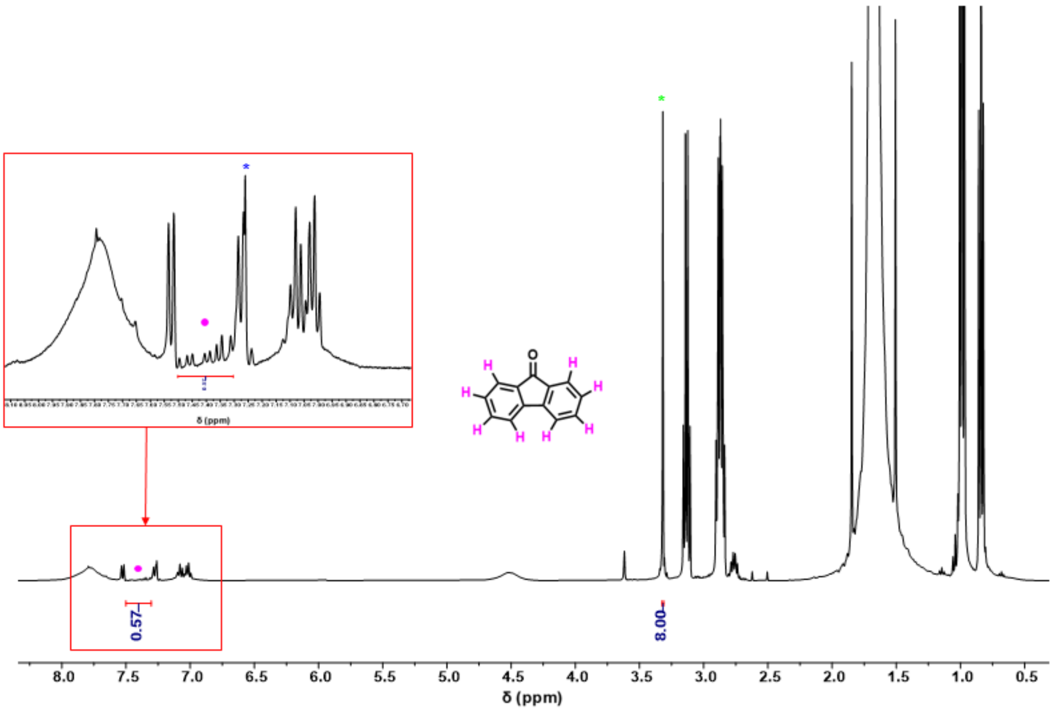

Figure S95.^{1}H-NMR spectra of the products from the photochemical oxidation of Fluorene in acetonitrile, showing the Fluorenone signals (pink). Solvent signals are marked with blue asterisk (chloroform-d) and internal standard as green asterisk (1,4-Dioxane) added after photoreaction. Coincident with reported spectrum. [21]

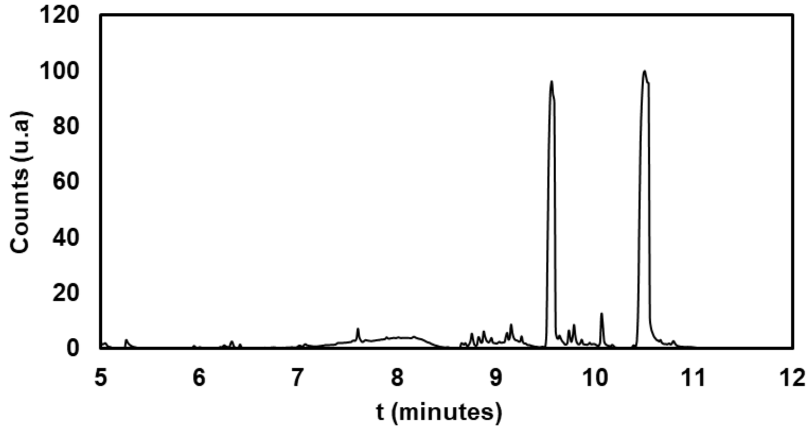

Figure S96. GC spectra of the products from the photochemical oxidation of Fluorene (retention time: 9.5 min) to Fluorenone (retention time: 10.5 min) in acetonitrile.

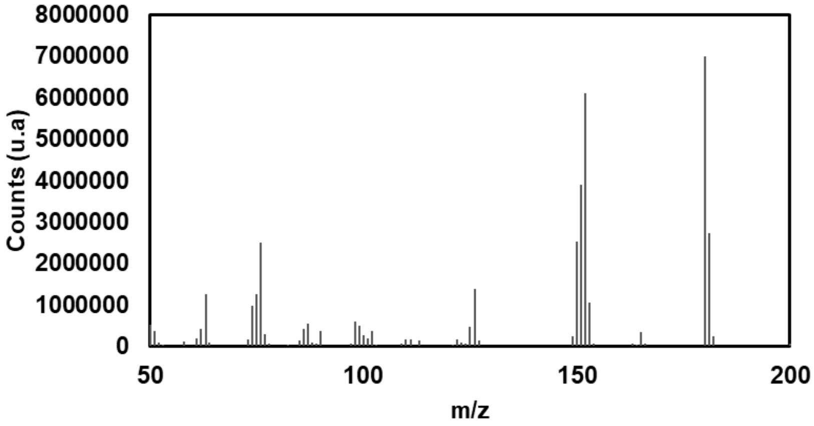

Figure S97. MS spectra of the product from the photochemical oxidation of Fluorene in acetonitrile, showing the Fluorenone signals corresponding to a retention time around 10.5 min.

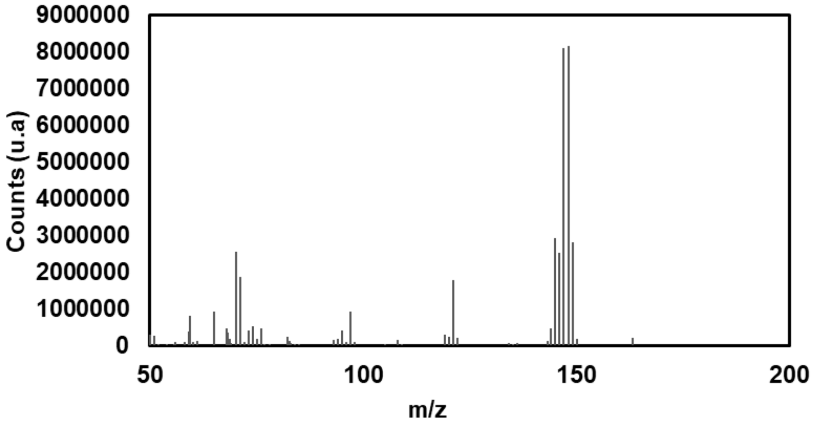

Figure S98. MS spectra of the substrate from the photochemical oxidation of Fluorene in acetonitrile, showing the Fluorene signals corresponding to a retention time around 9.5 min.

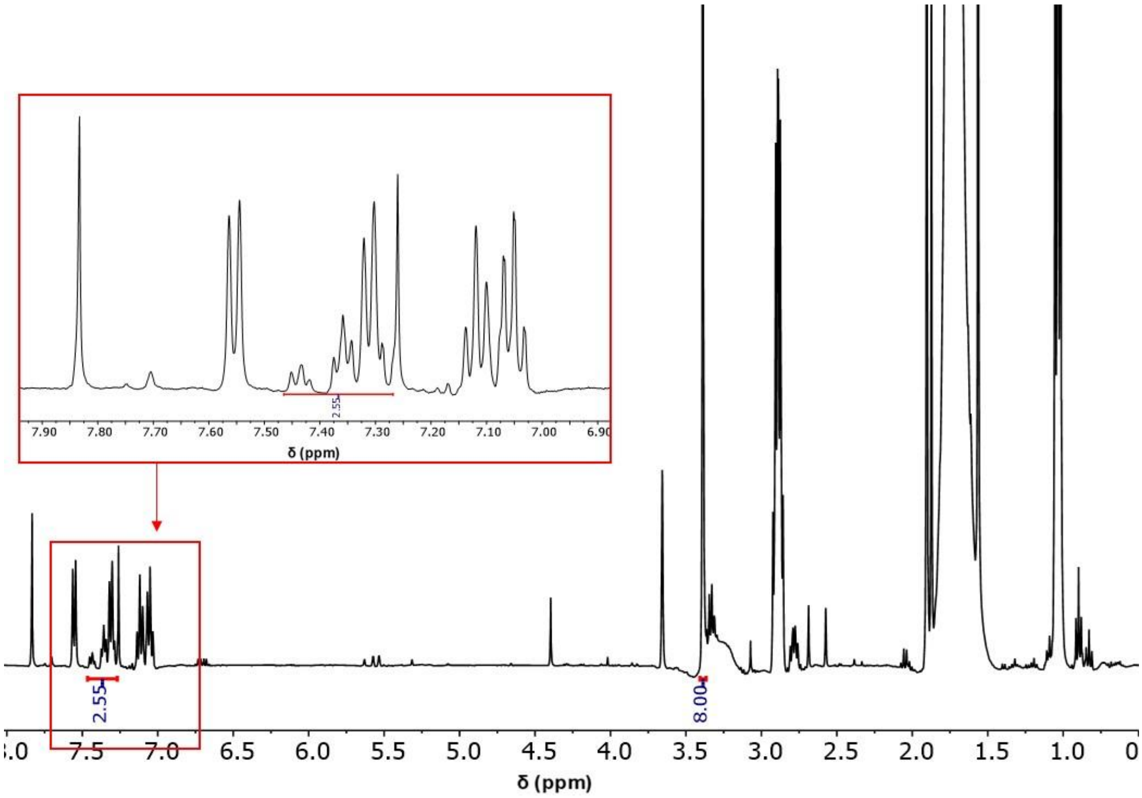

Figure S99.^{1}H-NMR spectra of the products from the photochemical oxidation of Fluorene in acetonitrile with added NaOH 0.1 M in water (200 μL; final NaOH concentration of 0.01 mM), showing the Fluorenone signals. In this experiment, 15.9 equiv. of Fluorenone were produced.

Table S5. Conversion percentage and equivalents obtained in the photocatalytic reaction studying the effect of H_2O and O_2 with $Na_2S_2O_8$ as SEA.

ACID	ATMOSPHERE	YIELD (%)	Equiv.
-	N_2	10	5
H_2O	N_2	2.5	1.3
-	Air (with O_2)	n.d.	n.d.

S.10.4. Representative stoichiometric reactions.

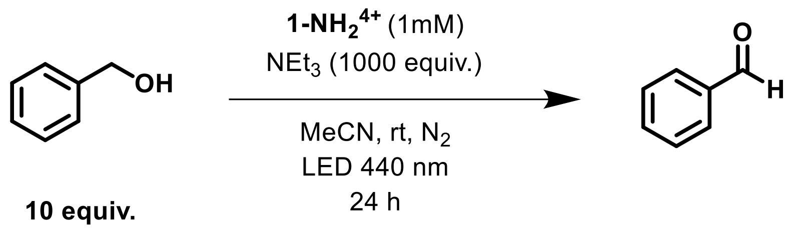

Figure S100. Scheme of photochemical stoichiometric reaction.

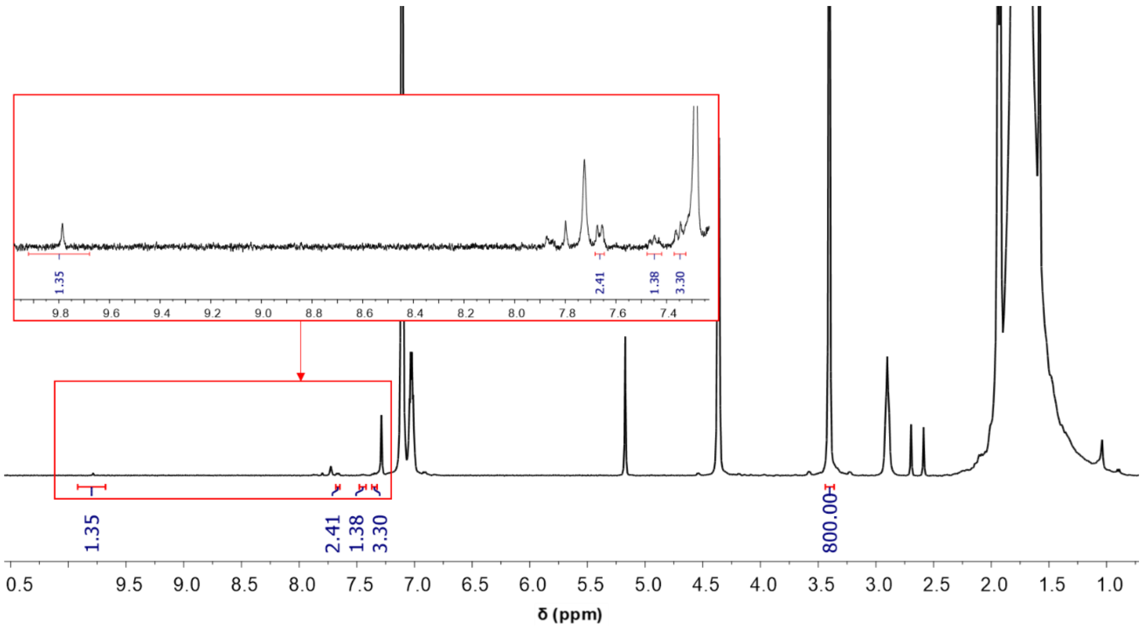

Figure S101. ^{1}H-NMR spectra of the products from the photochemical stoichiometric oxidation of Benzyl alcohol in acetonitrile-d3, showing the Benzaldehyde signals. Internal standard (1,4-Dioxane) added after photoreaction. Yield: 0.67 %.

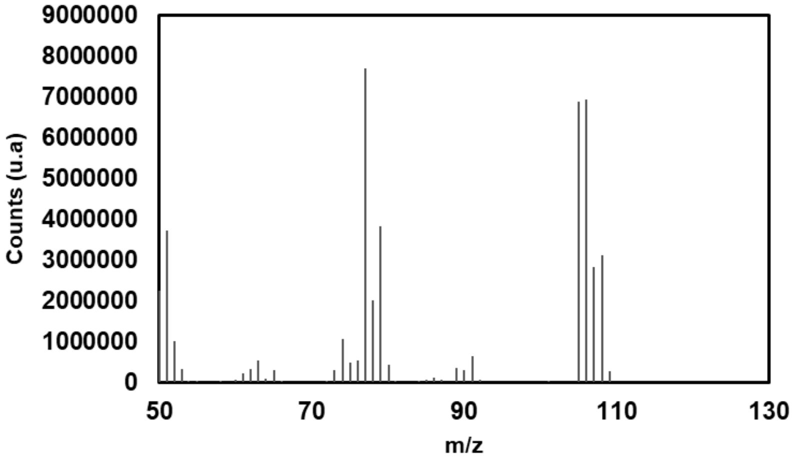

Figure S102. MS spectra of the product from the photochemical oxidation of Benzyl alcohol in acetonitrile, showing the Benzaldehyde signals.

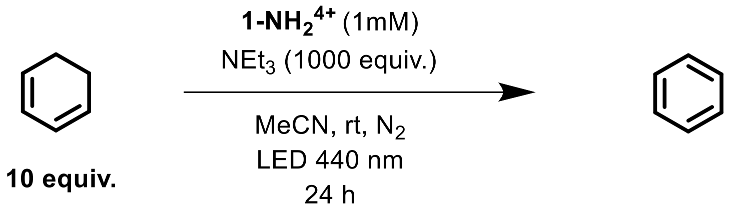

Figure S103. Scheme of photochemical stoichiometric reaction.

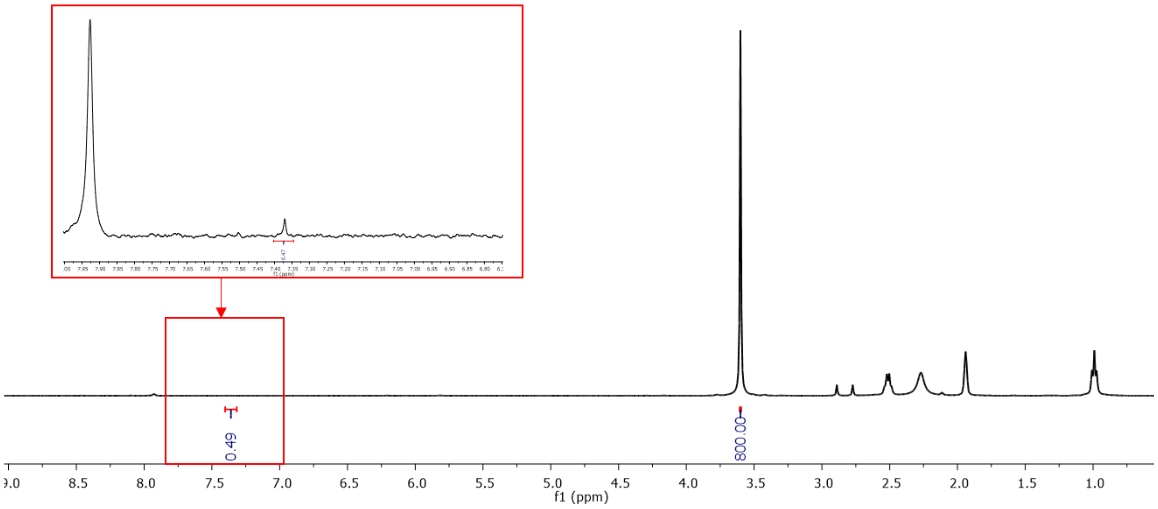

Figure S104. ^{1}H-NMR spectra of the products from the photochemical, stoichiometric oxidation of 1,3-cyclohexadiene in acetonitrile-d3, showing the Benzene signals. Internal standard (1,4-Dioxane) added after photoreaction. Yield: 5 %.

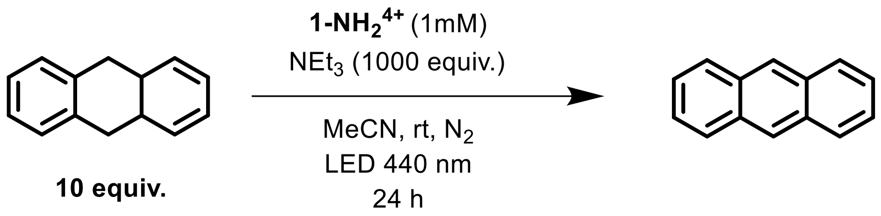

Figure S105. Scheme of photochemical stoichiometric reaction.

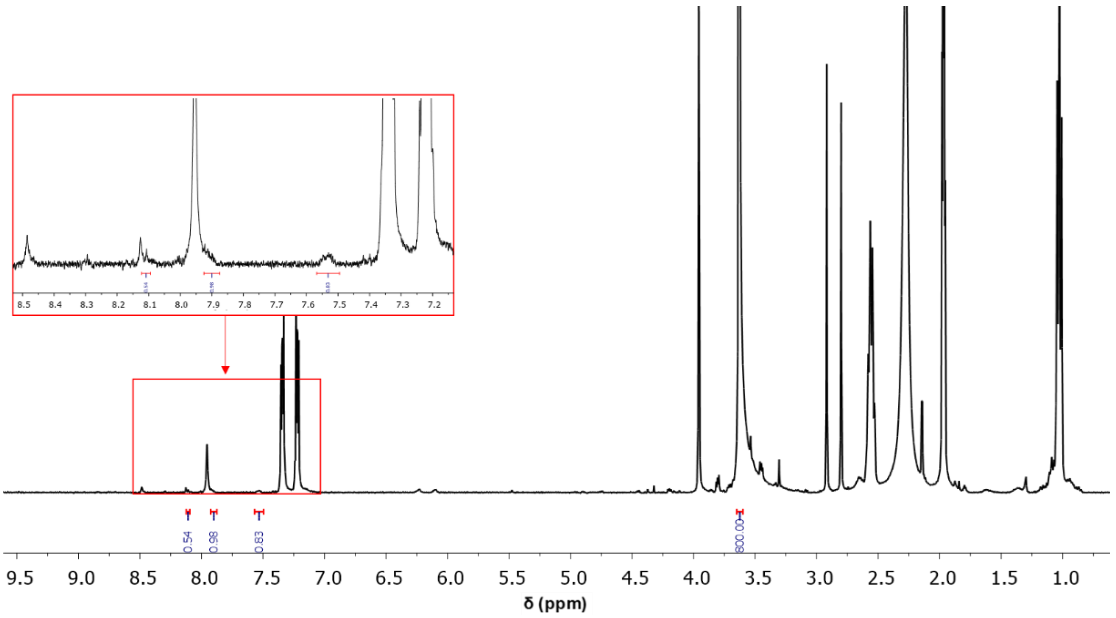

Figure S106. ^{1}H-NMR spectra of the products from the photochemical stoichiometric oxidation of 9,10-Dihydroanthracene in acetonitrile-d3, showing the Anthracene signals. Internal standard (1,4-Dioxane) added after photoreaction. Yield: 13 %.

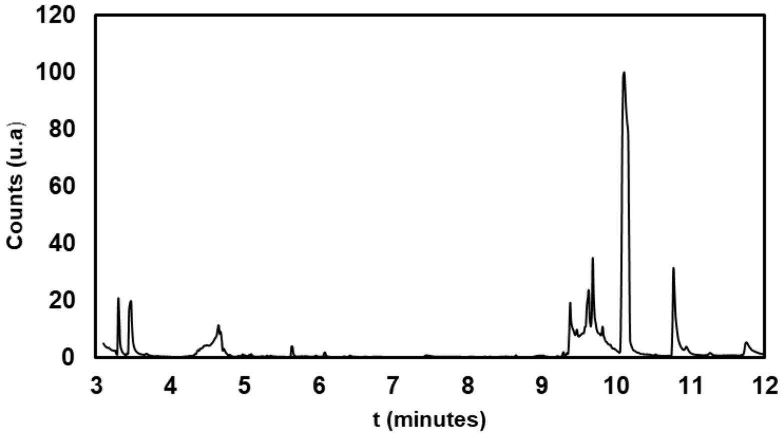

Figure S107. GC spectra of the products from the photochemical oxidation of 9,10-Dihydroanthracene (retention time: 10 min) to Anthracene (retention time: 10.8 min) in acetonitrile.

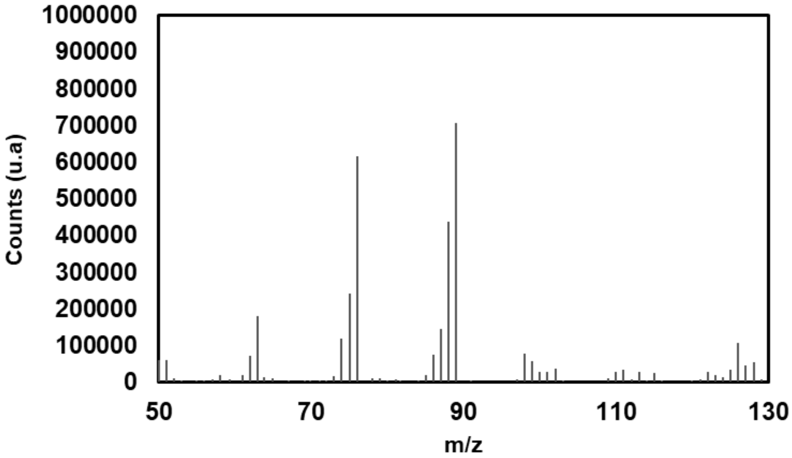

Figure S108. MS spectra of the product from the photochemical oxidation of 9,10-Dihydroanthracene in acetonitrile, showing the Anthracene signals.

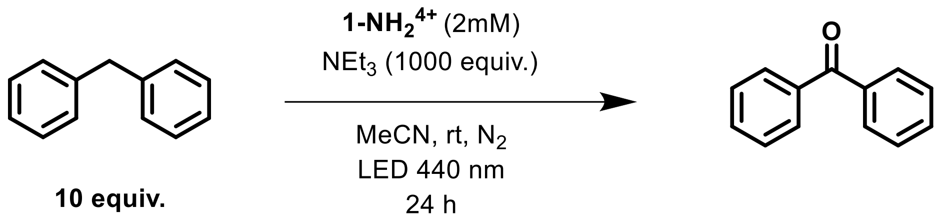

Figure S109. Scheme of photochemical stoichiometric reaction.

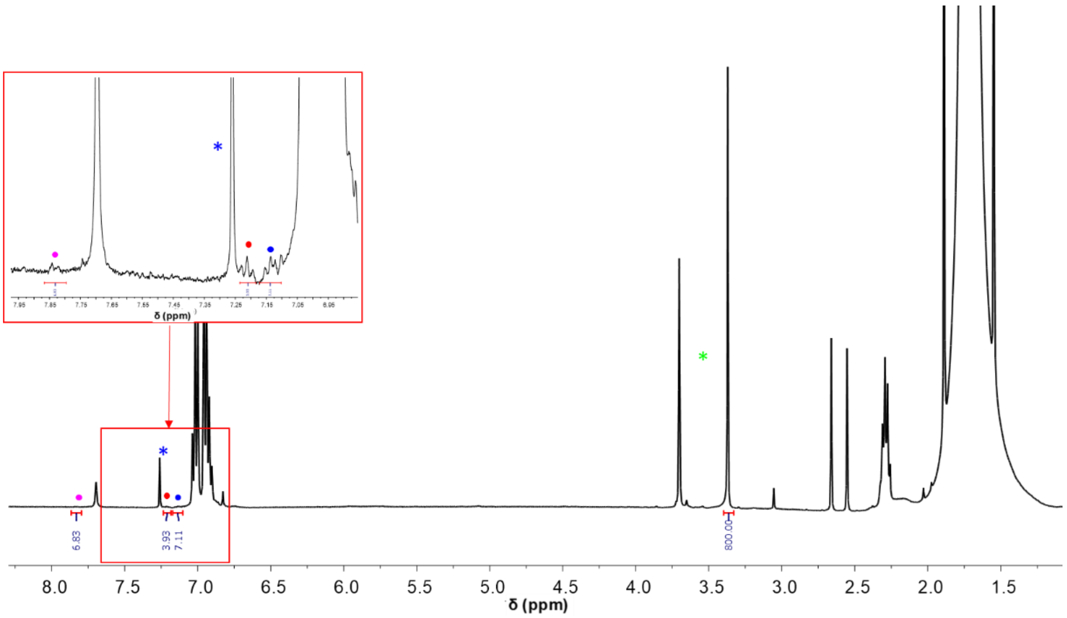

Figure S110.^{1}H-NMR spectra of the products from the photochemical stoichiometric oxidation of Diphenylmethane in acetonitrile, showing the Benzophenone signals. Internal standard (1,4-Dioxane) added after photoreaction. Yield: 40 %. *Note*: here double amount of **1-NH$_2$$^{4+}$** was employed to increase the ^{1}H NMR signals and, upon work up, the ^{1}H NMR was obtained in chloroform-d.

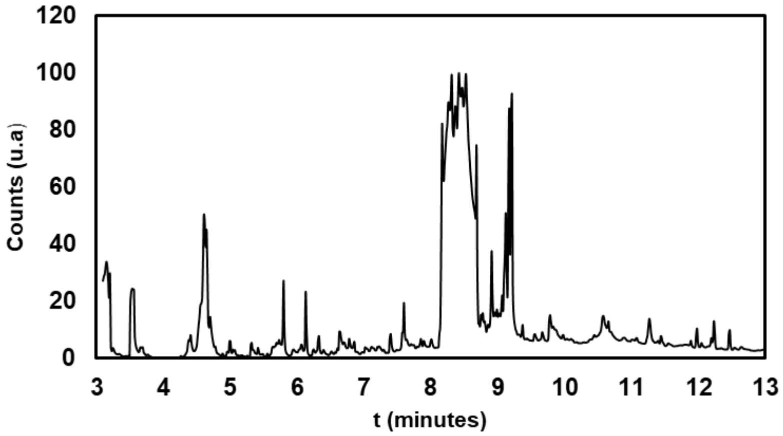

Figure S111. GC spectra of the products from the photochemical oxidation of Diphenylmethane (retention time: 8.5 min) to Benzophenone (retention time: 9.2 min) in acetonitrile.

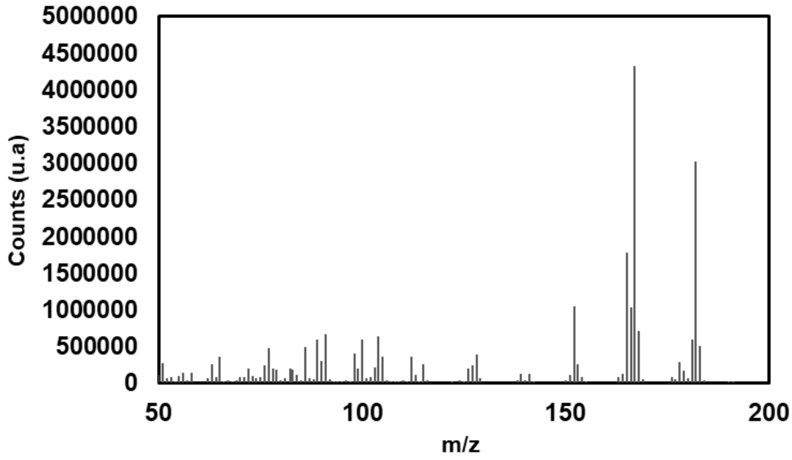

Figure S112. MS spectra of the product from the photochemical oxidation of Diphenylmethane in acetonitrile, showing the Benzophenone signals.

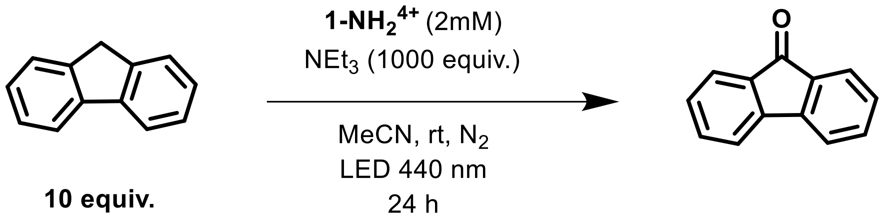

Figure S113. Scheme of photochemical stoichiometric reaction.

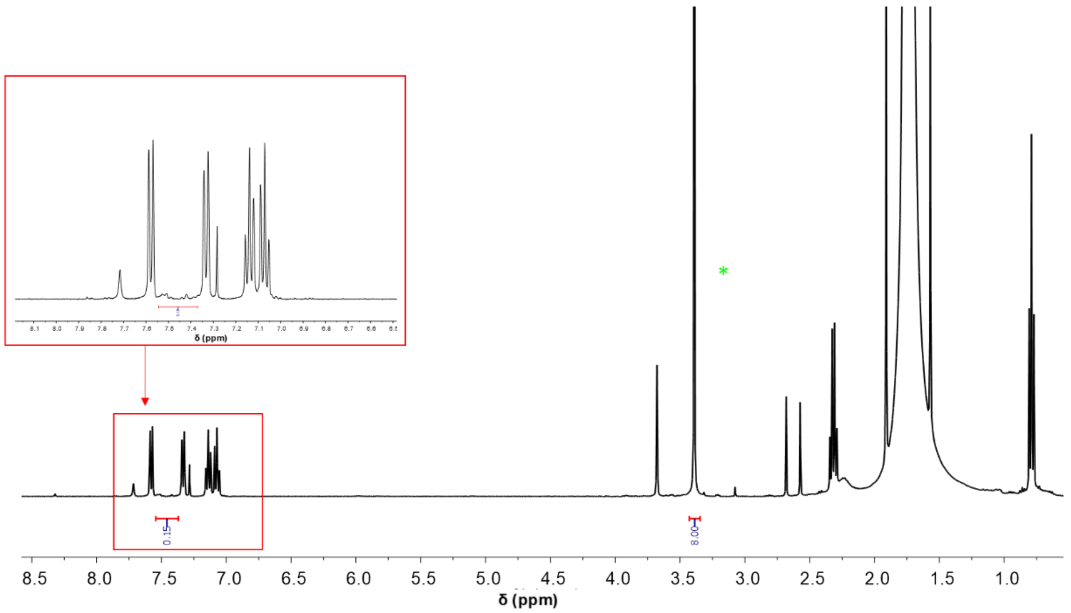

Figure S114. ^{1}H-NMR spectra of the products from the photochemical stoichiometric oxidation of Fluorene in acetonitrile, showing the Fluorenone signals. Internal standard (1,4-Dioxane) added after photoreaction. Yield: 46 %. Note: here double amount of **1-NH$_2$$^{4+}$** was employed to increase the ^{1}H NMR signals and, upon work up, the ^{1}H NMR was obtained in chloroform-d.

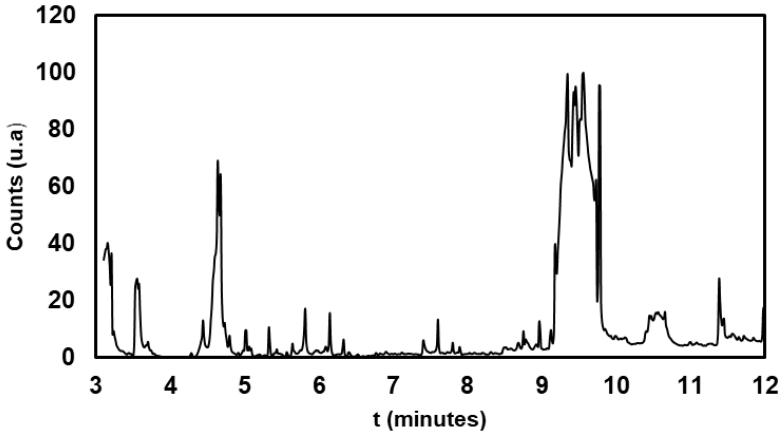

Figure S115. GC spectra of the products from the photochemical oxidation of Fluorene (retention time: 9.5 min) to Fluorenone (retention time: 10.5 min) in acetonitrile.

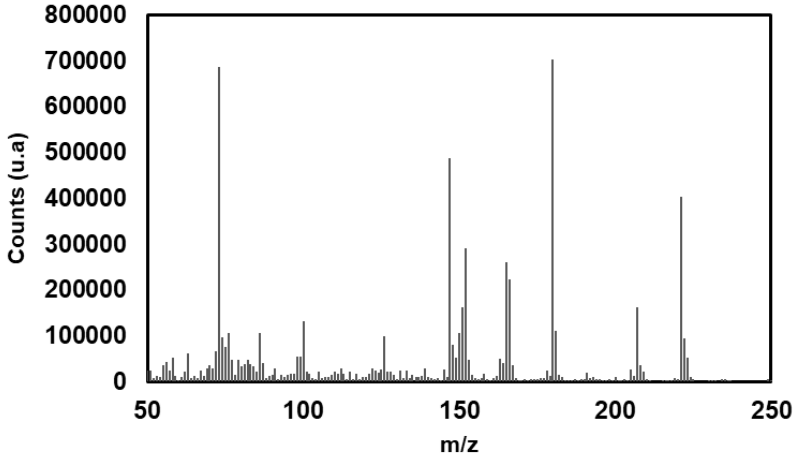

Figure S116. MS spectra of the product from the photochemical oxidation of Fluorene in acetonitrile, showing the Fluorenone signals.

S.10.5. Control reactions.

S.10.5.1. Benzyl alcohol.

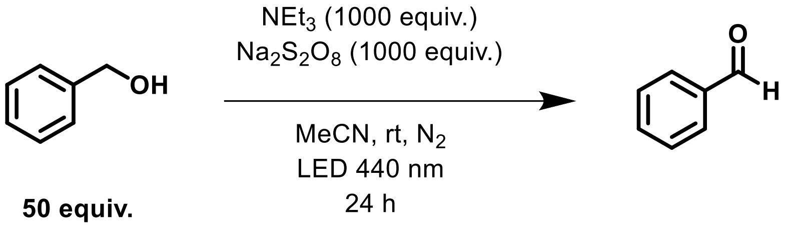

Figure S117. Scheme of photocatalytic reaction.

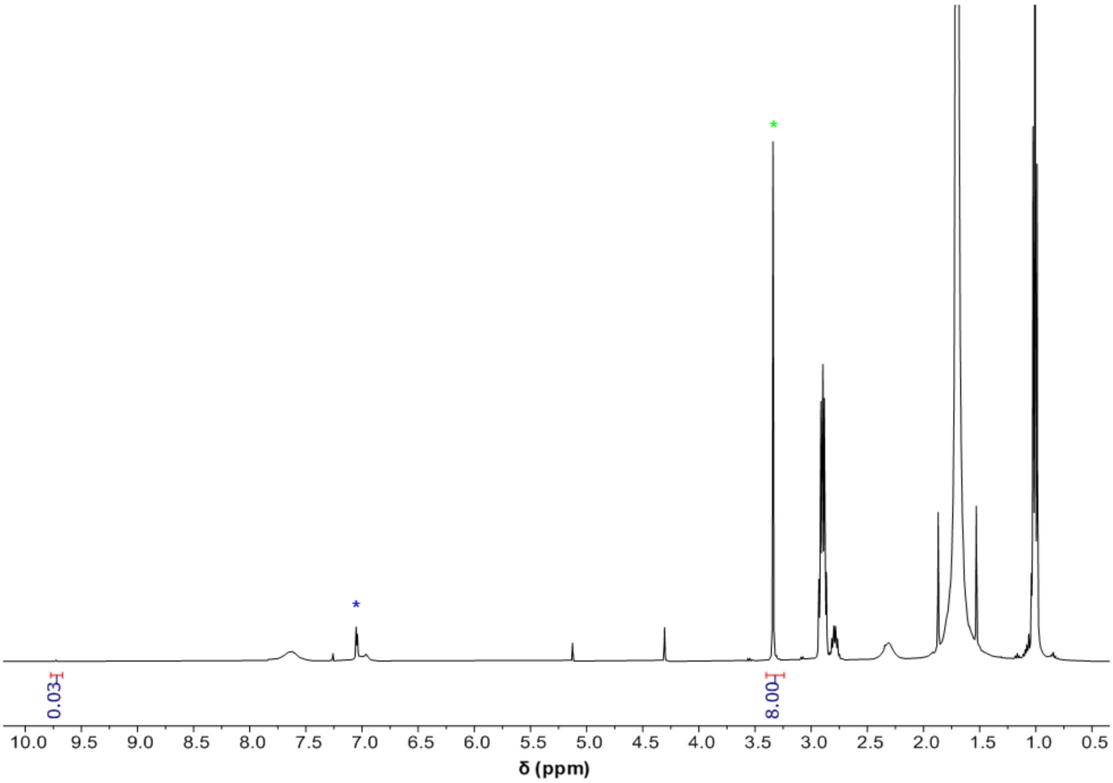

Figure S118. ^{1}H-NMR spectra (400 MHz) for the characterization of Benzaldehyde. Coincident with reported spectrum. The signal marked with blue asterisk correspond to the solvent chloroform-d and internal standard as green asterisk (1,4-Dioxane) added after photoreaction.

S.10.5.2. 1-phenylethanol.

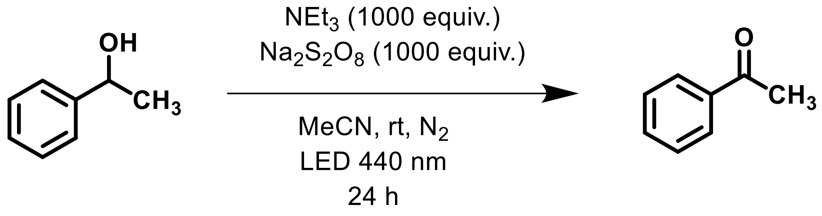

Figure S119. Scheme of photocatalytic reaction.

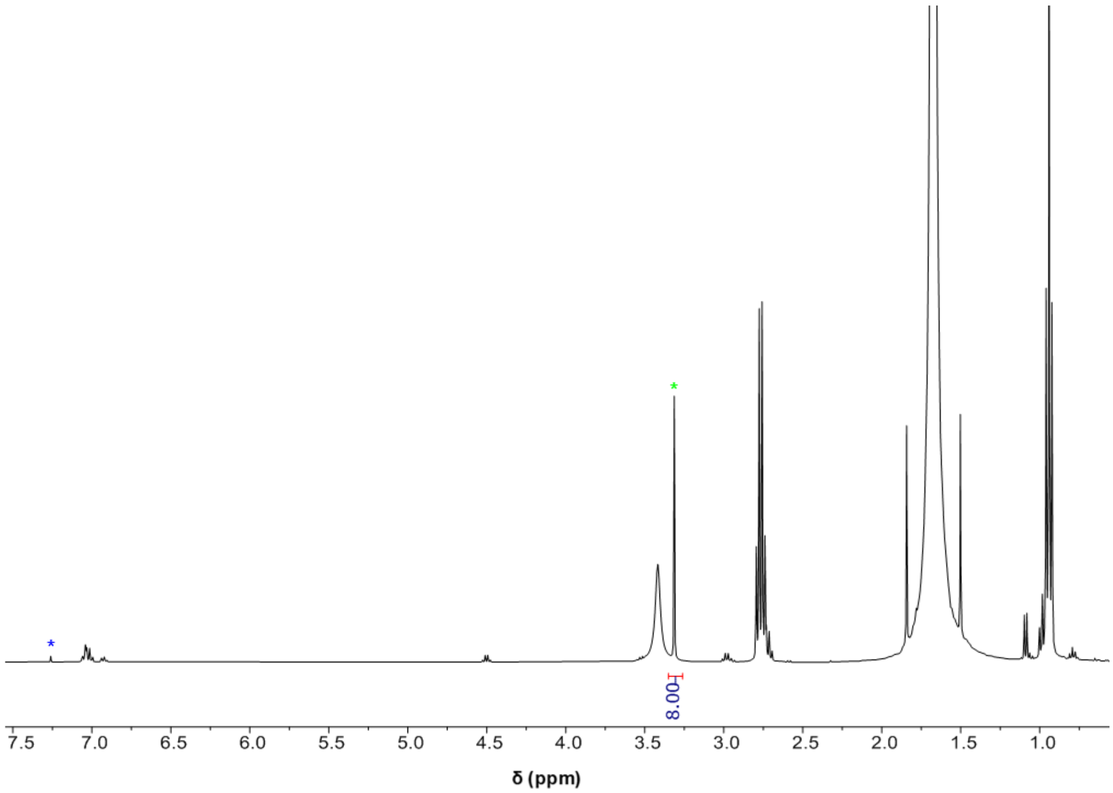

Figure S120. ^{1}H-NMR spectra (400 MHz) for the characterization of Acetophenone. Coincident with reported spectrum. The signal marked with blue asterisk correspond to the solvent chloroform-d and internal standard as green asterisk (1,4-Dioxane) added after photoreaction.

S.10.5.3. 1,3-cyclohexadiene.

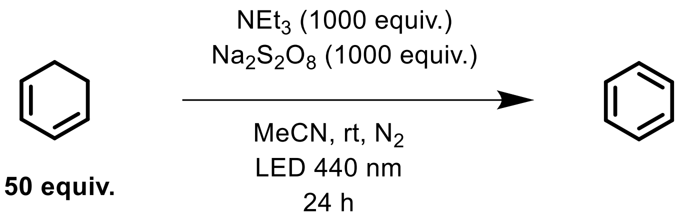

Figure S121. Scheme of photocatalytic reaction.

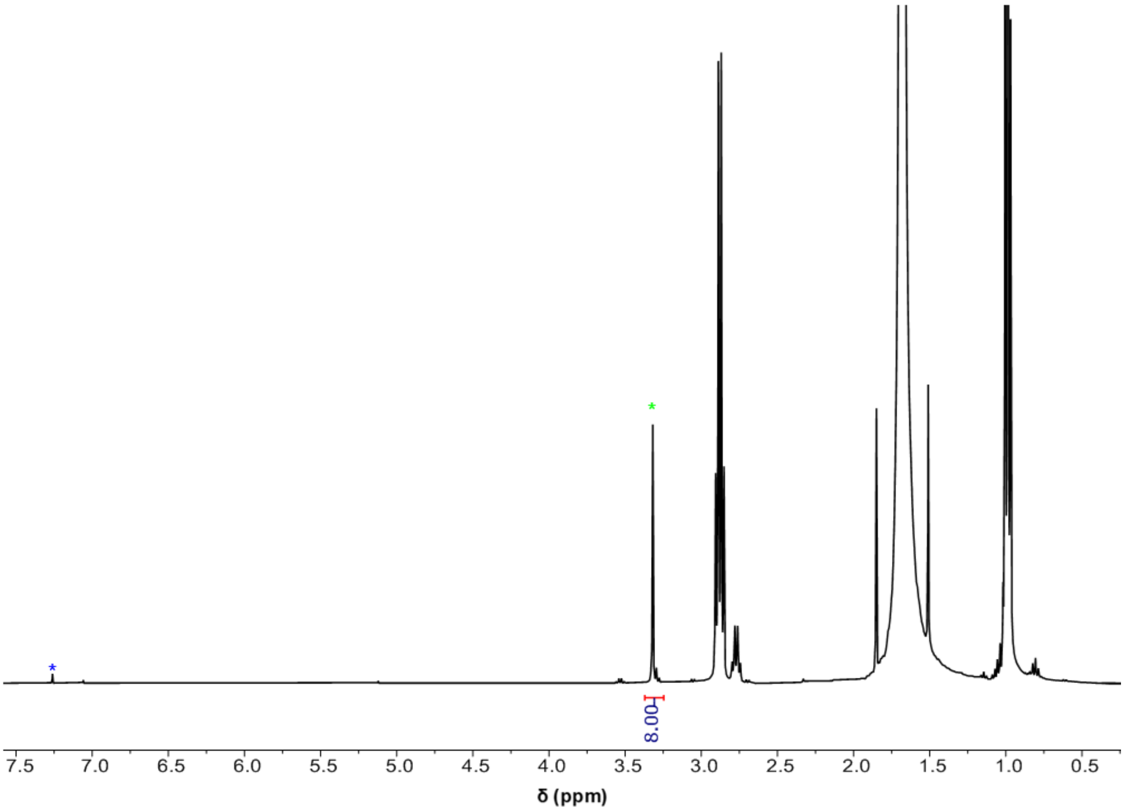

Figure S122. ^{1}H-NMR spectra (400 MHz) for the characterization of Benzene. Coincident with reported spectrum. The signal marked with blue asterisk correspond to the solvent chloroform-d and internal standard as green asterisk (1,4-Dioxane) added after photoreaction.

S.10.5.4. 9,10-Dihydroanthracene.

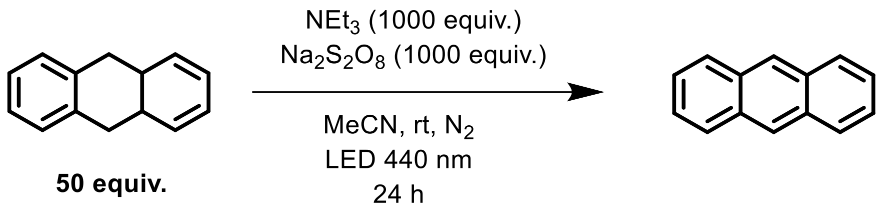

Figure S123. Scheme of photocatalytic reaction.

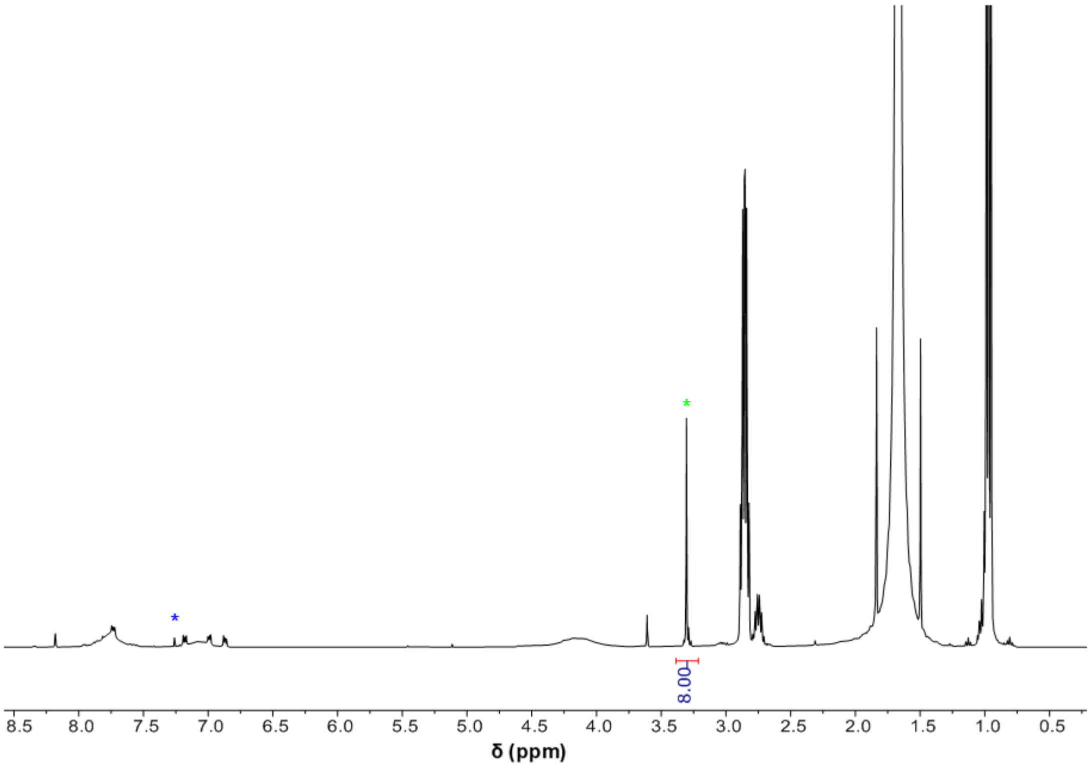

Figure S124. ^{1}H-NMR spectra (400 MHz) for the characterization of Anthracene. Coincident with reported spectrum. The signal marked with blue asterisk correspond to the solvent chloroform-d and internal standard as green asterisk (1,4-Dioxane) added after photoreaction.

S.10.5.5. Fluorene.

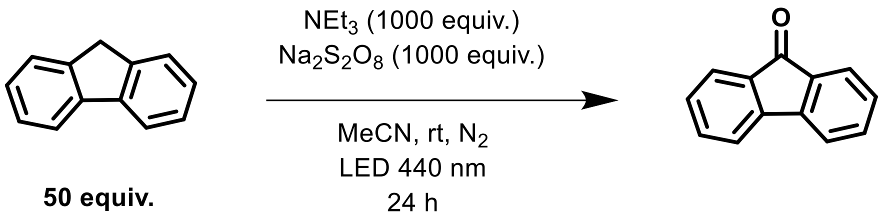

Figure S125. Scheme of photocatalytic reaction.

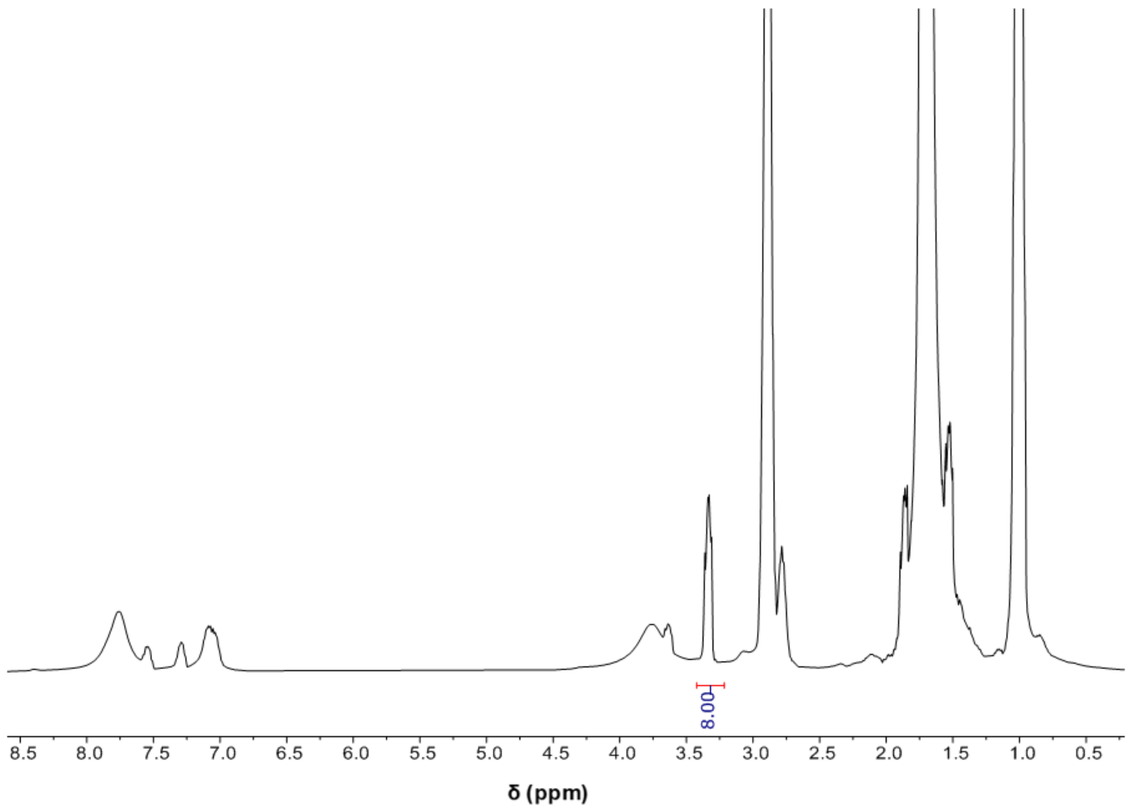

Figure S126. ^{1}H-NMR spectra (400 MHz) for the characterization of Fluorenol. Coincident with reported spectrum. The signal marked with blue asterisk correspond to the solvent chloroform-d and internal standard as green asterisk (1,4-Dioxane) added after photoreaction.

S11. Correlation Rate-Driving Force.

Table S6. Correlation Rate-Driving Force O-H / C-H bond.

Substrate	Ered	pKa	BDFE O-H/C-H	BDFE C-H	f	K_{SV} (M^{-1})
Benzyl alcohol	1.38	47.3	96.2 (O-H)	81.7	0.132	148.3
4-Methoxybenzyl alcohol	0.75	50.1	97.9 (O-H)	78.7	0.108	129.6
Isopropanol	2.06	52.8	98.9 (O-H)	89.2	0.123	122.6
1-Phenylethanol	1.44	48.0	98.3 (O-H)	75.7	0.122	164.0
1-(4-Methoxymethyl) phenyl-ethanol	0.78	48.9	97.8 (O-H)	76.8	0.032	154.3
1-(4-Trifluoromethyl) phenyl-ethanol	1.69	46.8	98.3 (O-H)	75.6	0.047	200.2
1,3-cyclohexadiene	0.56	47.7	69.9 (C-H)	69.9	0.330	93.4
9,10-Dihydroanthracene	1.10	43.1	74.8 (C-H)	74.8	0.081	370.3
Diphenylmethane	1.31	44.1	80.4 (C-H)	80.4	0.086	146.0
Fluorene	0.96	35.9	79.1 (C-H)	79.1	0.100	217.1

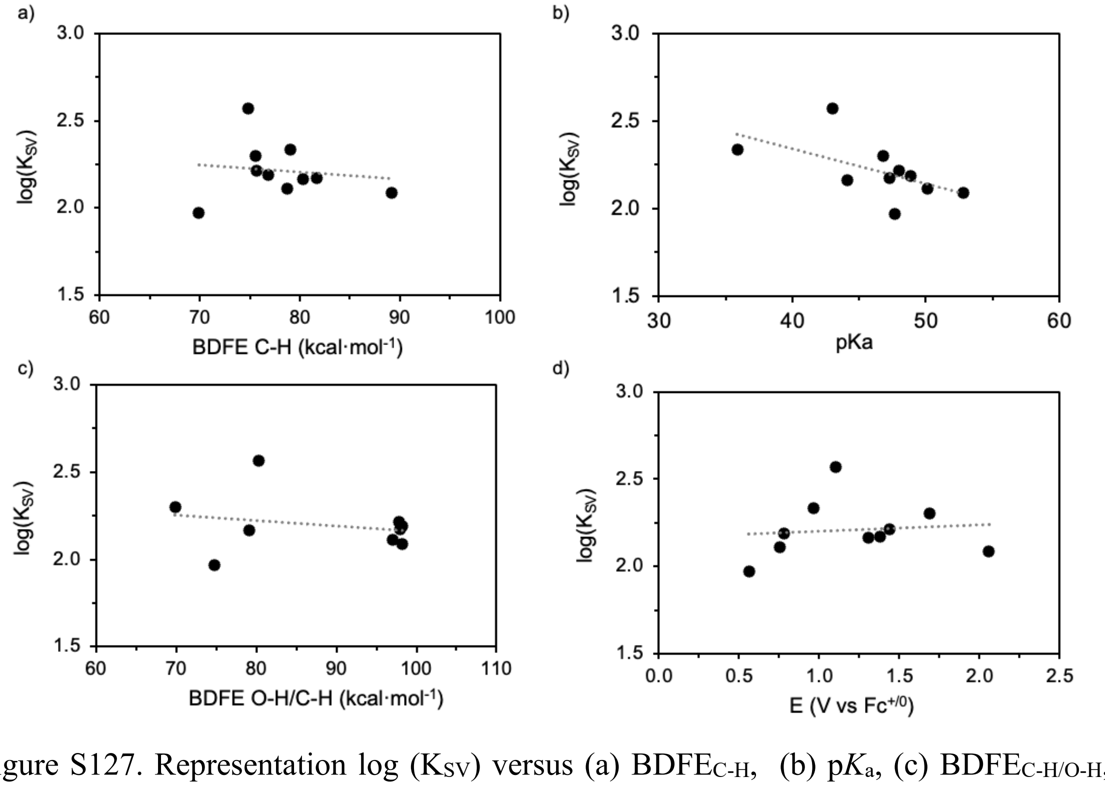

Figure S127. Representation log (K_{SV}) versus (a) $BDFE_{C-H}$, (b) pK_a, (c) $BDFE_{C-H/O-H}$, and (d) E_{red}.

Student's t-distribution values for each correlation were calculated dividing the slope of each linear fit by its standard error, both parameters were extracted using linear estimation function in excel. The critical t value for 8 degree of freedoms (given the 10-values dataset) and a one-tail 95% confidence interval is 1.8585, so only Student's t values higher than that critical value (considering absolute values) can confirm that the slope coefficient is lower than zero and that there is correlation in the dataset.

Table S7. Student's t-distribution analysis of the different proposed correlations.

Correlation	Student's t
**Log(K_{SV})-pK_a**	**1.99371** (> 1.8585)
Log(K_{SV})-$BDFE_{X\text{-}H}$	0.50913
Log(K_{SV})-$BDFE_{C\text{-}H}$	0.36143
Log(K_{SV})-E^0	0.28866

S.12. DFT calculations.

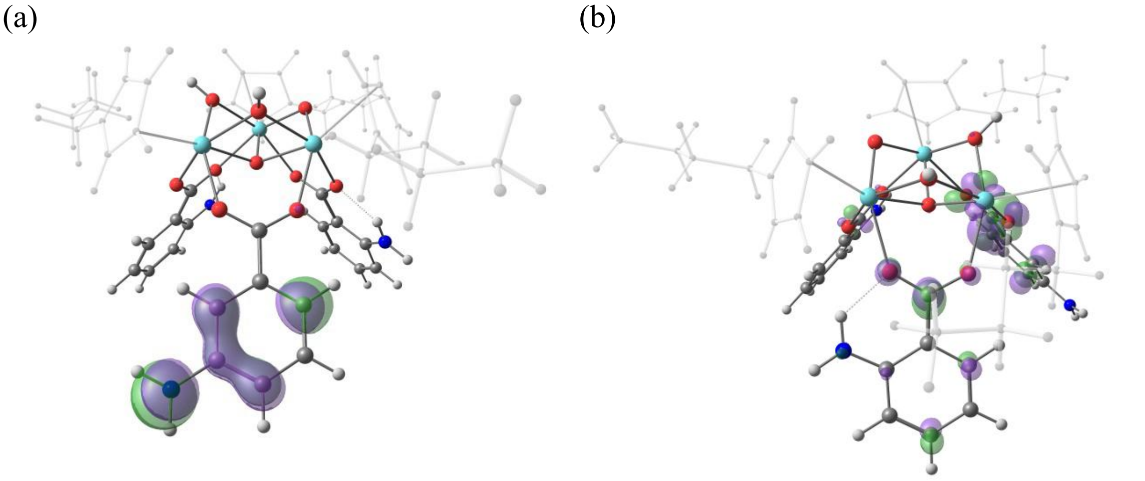

Figure S128. DFT optimized structure of **1-NH$_2$$^{4+}$** and representation of the (a) HOMO and (b) LUMO. The latter orbital is delocalized between the Zr d-orbitals and the ligand π* orbitals.

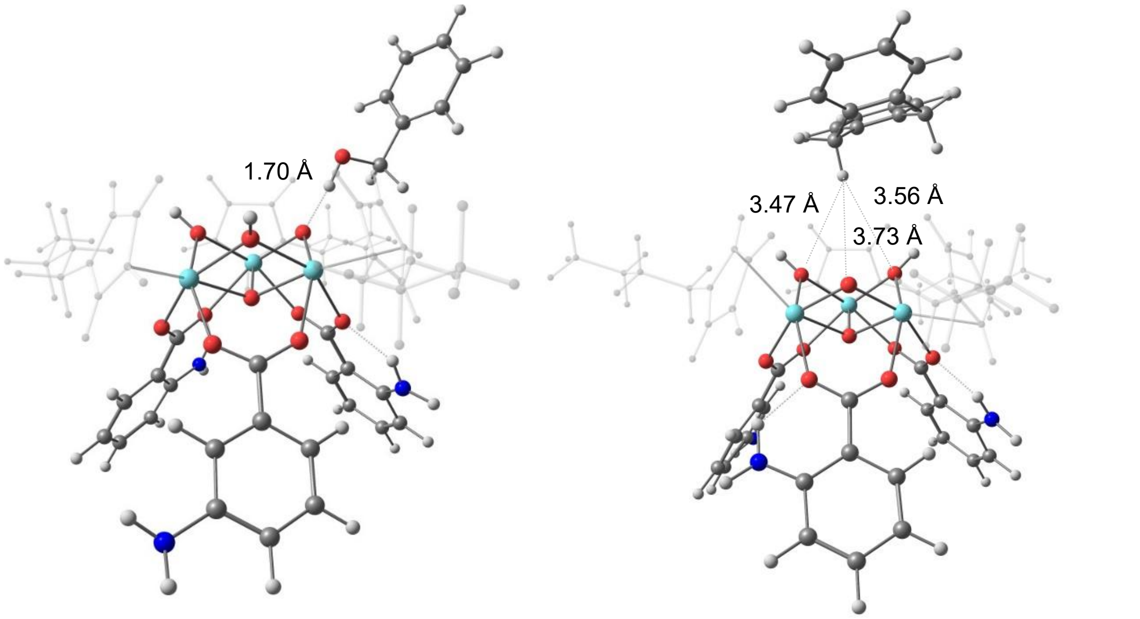

Figure S129. DFT optimized structure of **1-NH$_2$$^{4+}$** reproducing the pre-association equilibrium between Benzyl alcohol and 9,10-Dihydroanthracene as model substrates and a deprotonated node.

XYZ coordinates:

1-NH_2^{4+} deprotonated

Zr	6.284590000	14.470687000	14.466192000
O	4.757752000	13.506212000	15.754872000
O	5.133776000	13.110619000	13.244140000
O	7.509679000	14.710266000	12.545286000
C	7.174112000	14.594742000	11.279744000
C	8.238458000	14.534800000	10.261576000
C	7.864028000	14.443556000	8.897822000
C	6.620766000	15.739155000	16.867663000
C	8.565770000	15.424795000	15.681221000
H	9.512814000	15.022449000	15.341008000
C	7.801676000	14.927230000	16.784363000
C	10.176200000	14.390800000	8.212300000
C	8.804373000	14.372927000	7.877370000
O	5.926487000	14.543429000	10.896444000
C	9.636067000	14.560745000	10.596513000
C	10.582597000	14.480116000	9.534789000
C	6.676425000	16.743162000	15.853264000
C	7.879297000	16.545046000	15.113448000
C	2.466644000	11.587667000	16.714963000
H	2.589481000	12.392394000	17.428453000
C	2.751350000	9.610527000	15.556735000
H	3.157431000	8.664613000	15.218002000
C	3.295386000	10.431210000	16.592866000
C	2.688857000	16.898095000	11.785216000
H	2.826612000	17.608319000	12.590667000
C	2.978490000	15.724734000	9.826786000
H	3.372563000	15.394166000	8.872836000
C	3.494822000	16.814433000	10.607089000
C	1.415356000	11.473226000	15.751939000
H	0.623624000	12.196302000	15.603289000
C	1.576788000	10.244986000	15.036834000
C	1.681398000	15.886599000	11.729295000
H	0.909093000	15.714869000	12.467976000
C	1.853650000	15.169257000	10.505104000

Zr	3.954005000	14.495444000	12.104897000
O	4.942521000	15.684137000	13.482194000
O	3.619172000	12.517219000	10.950038000
C	3.669046000	11.241144000	11.253518000
C	3.779191000	10.237320000	10.180066000
C	3.858890000	8.866243000	10.534528000
C	3.997700000	8.212300000	8.212300000
C	3.966798000	7.860570000	9.581145000
O	3.597838000	10.828472000	12.499843000
C	3.809476000	10.590587000	8.786517000
C	3.921408000	9.542422000	7.826072000
Zr	3.900514000	11.948710000	14.448115000
O	2.618588000	13.552048000	13.621584000
O	5.847392000	10.809602000	14.751271000
C	7.094867000	11.197491000	14.639645000
C	8.162895000	10.158236000	14.549554000
C	9.513801000	10.554866000	14.462879000
C	10.176200000	8.212300000	14.390800000
C	10.505962000	9.569955000	14.385110000
O	7.450487000	12.445134000	14.622912000
C	7.819267000	8.797436000	14.556409000
C	8.820754000	7.800999000	14.476932000
C	4.156386000	18.947992000	9.367410000
H	3.683643000	18.573732000	8.448406000
H	3.385863000	19.505183000	9.918601000
C	5.311594000	19.900977000	8.998431000
H	6.085140000	19.339207000	8.455802000
H	5.780634000	20.273422000	9.920165000
C	4.847567000	21.093741000	8.140628000
H	5.685467000	21.758590000	7.896884000
H	4.406161000	20.750834000	7.196273000
H	4.090705000	21.687428000	8.668751000
C	8.192152000	13.812614000	17.727164000
H	7.290017000	13.333389000	18.130579000
H	8.749961000	13.039182000	17.183521000

C	9.061883000	14.304524000	18.916468000
H	9.975275000	14.772853000	18.523763000
H	8.514780000	15.083373000	19.465982000
C	9.443934000	13.166650000	19.884957000
H	8.527582000	12.701305000	20.275273000
H	9.982922000	12.385266000	19.330619000
C	10.312862000	13.652981000	21.060562000
H	10.565988000	12.826910000	21.736561000
H	11.252263000	14.091021000	20.700370000
H	9.788946000	14.418255000	21.647151000
C	0.657125000	9.671433000	13.978466000
H	1.231159000	8.993704000	13.333010000
H	-0.106877000	9.047143000	14.469783000
C	-0.067650000	10.716029000	13.099183000
H	-0.658760000	11.388231000	13.736147000
H	0.677415000	11.336542000	12.582771000
C	-0.999525000	10.065631000	12.055146000
H	-0.414780000	9.377580000	11.428206000
H	-1.753936000	9.456852000	12.573345000
C	-1.704303000	11.099054000	11.156134000
H	-2.324350000	11.782519000	11.750069000
H	-0.973295000	11.702738000	10.603045000
H	-2.355858000	10.607744000	10.422963000
H	1.249649000	14.338136000	10.163422000
H	5.829869000	15.623432000	17.599158000
H	8.214097000	17.135768000	14.270584000
H	5.932779000	17.507439000	15.671059000
H	4.165495000	10.202287000	17.195172000
H	3.837882000	8.613190000	11.589068000
H	3.945597000	9.811979000	6.773605000
H	4.027513000	6.820319000	9.883730000
H	9.772618000	11.606661000	14.456428000
H	11.550546000	9.860737000	14.318100000
H	6.805101000	14.426752000	8.663624000
H	8.490576000	14.304684000	6.840954000
H	11.639839000	14.493672000	9.785631000
H	4.082126000	7.439510000	7.453333000
H	10.956853000	7.458751000	14.330244000
H	10.926496000	14.333732000	7.428232000
C	4.625497000	17.741815000	10.226274000
H	5.390189000	17.189724000	9.664385000
H	5.109567000	18.125644000	11.133853000
H	1.642205000	13.529242000	13.583334000
H	4.747167000	13.500201000	16.731882000
H	6.776015000	8.505533000	14.622180000
N	3.731575000	11.887538000	8.349391000
H	3.764612000	12.097573000	7.360554000
H	3.673289000	12.633475000	9.035120000
N	10.092093000	14.656978000	11.886322000
H	11.084991000	14.659805000	12.078733000
H	9.416751000	14.709237000	12.641742000
N	8.486342000	6.457400000	14.491851000
H	9.196432000	5.745363000	14.392672000
H	7.519605000	6.163592000	14.505945000

1-NH_2^{4+}···BnOH

Zr	6.326912000	14.395193000	14.415064000
O	4.877014000	13.372614000	15.658710000
O	5.237652000	13.090437000	13.168595000
O	7.543434000	14.575242000	12.562024000
C	7.197358000	14.525231000	11.322727000
C	8.257248000	14.435433000	10.290270000
C	7.869541000	14.399865000	8.926425000
C	6.659411000	15.485000000	16.752324000
C	8.544956000	15.203621000	15.475204000
H	9.470915000	14.792304000	15.064641000
C	7.807405000	14.649837000	16.565069000
C	10.176200000	14.390802000	8.212298000
C	8.799905000	14.375360000	7.890191000
O	5.976959000	14.587727000	10.939543000
C	9.660379000	14.418577000	10.616574000
C	10.594975000	14.407091000	9.538107000

C	6.704787000	16.550540000	15.809714000	C	10.482648000	9.544207000	14.084295000
C	7.867176000	16.372272000	15.010130000	O	7.469764000	12.443516000	14.569783000
C	2.703720000	11.562591000	16.524499000	C	7.855302000	8.839159000	14.748241000
H	2.794794000	12.338476000	17.288257000	C	8.849590000	7.831570000	14.722846000
C	3.070767000	9.684955000	15.247084000	C	4.285915000	18.566487000	9.107194000
H	3.510720000	8.764199000	14.855686000	H	3.691313000	18.163703000	8.263588000
C	3.546356000	10.427058000	16.367749000	H	3.594698000	19.197867000	9.699605000
C	2.878552000	16.760785000	11.682271000	C	5.428945000	19.435050000	8.558851000
H	3.057061000	17.535475000	12.431780000	H	6.120484000	18.797658000	7.973026000
C	3.113236000	15.379083000	9.860469000	H	6.021870000	19.831581000	9.406705000
H	3.488398000	14.940679000	8.932046000	C	4.943176000	20.598674000	7.686544000
C	3.658587000	16.534370000	10.508262000	H	5.789757000	21.203647000	7.313876000
C	1.707847000	11.514439000	15.504281000	H	4.380714000	20.233187000	6.807011000
H	0.905747000	12.243452000	15.370055000	H	4.273830000	21.272583000	8.253326000
C	1.914606000	10.339847000	14.716898000	C	8.201676000	13.469879000	17.418321000
C	1.873298000	15.754920000	11.759876000	H	7.295805000	12.907703000	17.715184000
H	1.123268000	15.662031000	12.549761000	H	8.821791000	12.779345000	16.820578000
C	2.010969000	14.903245000	10.623984000	C	8.975619000	13.876490000	18.692218000
Zr	4.092432000	14.480340000	12.086546000	H	9.877333000	14.450509000	18.401248000
O	4.994797000	15.638969000	13.496672000	H	8.351322000	14.564037000	19.296167000
O	3.805033000	12.544808000	10.888823000	C	9.390612000	12.672673000	19.552350000
C	3.830006000	11.306265000	11.207054000	H	8.486533000	12.092137000	19.823093000
C	3.923870000	10.276710000	10.145541000	H	10.020126000	11.993306000	18.944267000
C	3.919469000	8.909034000	10.522601000	C	10.145009000	13.065926000	20.827712000
C	3.997698000	8.212300000	8.212298000	H	10.430591000	12.175433000	21.416890000
C	3.957507000	7.879452000	9.585649000	H	11.071208000	13.622712000	20.591371000
O	3.733598000	10.899910000	12.427145000	H	9.525048000	13.712266000	21.476934000
C	3.988205000	10.612676000	8.745451000	C	1.047414000	9.814102000	13.597185000
C	4.016830000	9.540808000	7.802616000	H	1.694590000	9.312264000	12.855974000
Zr	3.992369000	11.906180000	14.331740000	H	0.395875000	9.021132000	14.018800000
O	2.763509000	13.479884000	13.506275000	C	0.165643000	10.855561000	12.891765000
O	5.889589000	10.830263000	14.617523000	H	-0.513520000	11.329684000	13.626929000
C	7.107616000	11.227834000	14.541475000	H	0.807439000	11.663765000	12.491161000
C	8.174917000	10.177175000	14.457507000	C	-0.670446000	10.256814000	11.749250000
C	9.496253000	10.540490000	14.118231000	H	0.008317000	9.781620000	11.013808000
C	10.176202000	8.212298000	14.390804000	H	-1.304855000	9.442478000	12.151688000

C	-1.553645000	11.287013000	11.035919000
H	-2.273719000	11.752642000	11.734685000
H	-0.945465000	12.098957000	10.594770000
H	-2.133745000	10.821802000	10.218350000
H	1.401820000	14.026024000	10.395845000
H	5.883416000	15.337388000	17.508991000
H	8.175443000	16.999788000	14.170799000
H	5.938308000	17.317241000	15.677905000
H	4.419834000	10.184088000	16.976384000
H	3.881973000	8.683436000	11.592551000
H	4.054822000	9.791380000	6.736278000
H	3.951700000	6.832778000	9.906352000
H	9.729491000	11.581044000	13.875177000
H	11.513595000	9.806741000	13.820385000
H	6.796770000	14.390622000	8.710459000
H	8.471526000	14.349539000	6.846158000
H	11.663773000	14.410056000	9.781045000
H	4.019003000	7.419313000	7.455578000
H	10.961265000	7.447362000	14.370280000
H	10.927472000	14.384035000	7.413950000
C	4.781615000	17.394156000	9.983055000
H	5.471840000	16.766868000	9.391938000
H	5.366786000	17.799712000	10.828110000
H	1.792039000	13.538435000	13.530878000
H	4.856343000	13.361373000	16.631993000
H	6.824318000	8.579463000	15.009762000
N	4.031162000	11.896380000	8.303237000
H	3.991764000	12.095425000	7.306399000
H	3.940643000	12.646671000	8.989679000
N	10.120532000	14.394257000	11.898340000
H	11.112896000	14.536191000	12.075369000
H	9.444484000	14.533093000	12.651122000
N	8.526509000	6.506746000	14.960772000
H	9.293842000	5.896212000	15.243647000
H	7.668012000	6.335992000	15.485501000
C	4.401278000	22.916866000	15.664283000
C	3.516690000	22.434086000	14.685175000
C	3.786220000	21.223711000	14.028951000
C	4.940545000	20.475694000	14.339845000
C	5.817884000	20.969123000	15.325077000
C	5.554238000	22.181980000	15.981995000
H	4.193507000	23.863897000	16.175261000
H	2.616752000	23.005496000	14.430099000
H	3.095358000	20.854694000	13.261217000
H	6.718707000	20.396819000	15.577787000
H	6.250276000	22.553401000	16.742855000
C	5.200422000	19.147238000	13.657504000
H	4.981061000	19.246178000	12.573340000
H	6.274735000	18.889679000	13.752839000
O	4.372579000	18.149011000	14.254079000
H	4.647023000	17.241642000	13.910003000

1-NH_2^{4+}···DHA

Zr	6.305520000	14.403495000	14.403996000
O	4.857467000	13.387737000	15.669073000
O	5.237653000	13.049158000	13.179784000
O	7.536489000	14.532293000	12.557140000
C	7.188454000	14.489504000	11.317866000
C	8.251774000	14.413937000	10.285566000
C	7.868059000	14.410378000	8.920762000
C	6.498686000	15.780137000	16.592801000
C	8.476789000	15.155075000	15.606277000
H	9.389255000	14.605123000	15.362330000
C	7.561780000	14.817723000	16.647656000
C	10.176200000	14.390802000	8.212298000
C	8.801050000	14.396942000	7.886318000
O	5.969150000	14.546814000	10.934563000
C	9.653752000	14.380381000	10.615203000
C	10.591344000	14.379103000	9.539550000
C	6.776104000	16.708958000	15.551215000
C	7.990514000	16.315720000	14.928636000
C	2.729451000	11.525662000	16.565375000

H	2.833829000	12.287299000	17.341298000
C	3.067040000	9.664624000	15.256787000
H	3.494442000	8.745589000	14.847619000
C	3.559854000	10.385414000	16.383687000
C	2.661225000	16.625349000	11.879435000
H	2.624070000	17.272763000	12.758526000
C	3.336893000	15.639275000	9.917304000
H	3.904051000	15.413012000	9.010804000
C	3.635976000	16.694410000	10.840979000
C	1.722038000	11.501254000	15.555593000
H	0.924325000	12.238594000	15.442769000
C	1.911178000	10.337628000	14.748118000
C	1.775844000	15.542373000	11.602242000
H	0.919497000	15.240676000	12.210913000
C	2.185115000	14.941323000	10.377222000
Zr	4.079365000	14.449991000	12.099556000
O	4.981664000	15.590518000	13.477749000
O	3.765943000	12.518547000	10.914841000
C	3.795413000	11.279717000	11.231647000
C	3.875528000	10.257552000	10.161585000
C	3.929067000	8.888657000	10.528777000
C	3.997698000	8.212300000	8.212298000
C	3.989826000	7.867924000	9.583165000
O	3.722402000	10.870254000	12.451287000
C	3.888562000	10.605635000	8.763155000
C	3.953088000	9.543104000	7.812135000
Zr	3.998307000	11.889774000	14.357771000
O	2.757634000	13.459153000	13.534841000
O	5.897417000	10.829211000	14.666252000
C	7.114640000	11.234044000	14.601706000
C	8.182680000	10.184009000	14.506905000
C	9.502026000	10.547384000	14.160919000
C	10.176202000	8.212298000	14.390804000
C	10.484223000	9.547925000	14.101860000
O	7.470924000	12.450044000	14.645803000
C	7.862363000	8.843056000	14.783640000

C	8.852199000	7.832139000	14.733263000
C	4.312369000	18.974358000	9.917480000
H	3.987560000	18.671390000	8.902484000
H	3.427305000	19.430893000	10.403052000
C	5.430673000	20.022191000	9.805698000
H	6.315169000	19.559564000	9.324576000
H	5.754429000	20.315566000	10.823893000
C	5.018571000	21.273399000	9.021253000
H	5.844843000	22.005796000	8.970225000
H	4.730371000	21.020326000	7.983692000
H	4.154122000	21.777045000	9.493258000
C	7.722667000	13.715922000	17.666066000
H	6.726157000	13.347127000	17.975807000
H	8.247002000	12.862246000	17.200499000
C	8.497182000	14.158997000	18.927609000
H	9.502844000	14.514623000	18.628947000
H	7.983465000	15.027282000	19.385284000
C	8.637819000	13.039295000	19.970391000
H	7.628344000	12.688960000	20.263608000
H	9.140126000	12.169264000	19.503016000
C	9.413535000	13.464900000	21.222541000
H	9.485481000	12.636592000	21.950830000
H	10.443389000	13.779169000	20.969273000
H	8.921492000	14.315332000	21.730807000
C	1.029462000	9.839102000	13.627152000
H	1.664516000	9.330159000	12.880420000
H	0.361090000	9.057134000	14.043115000
C	0.170659000	10.906764000	12.931957000
H	-0.505499000	11.381463000	13.669689000
H	0.830098000	11.709346000	12.549403000
C	-0.666179000	10.343380000	11.772459000
H	0.010022000	9.865543000	11.036397000
H	-1.322526000	9.536774000	12.155040000
C	-1.518348000	11.404315000	11.066371000
H	-2.235538000	11.874900000	11.764769000
H	-0.886854000	12.209161000	10.644916000

H	-2.099521000	10.965422000	10.235157000	N	8.526507000	6.505182000	14.956667000
H	1.727287000	14.072647000	9.899995000	H	9.294612000	5.888312000	15.223241000
H	5.628328000	15.802622000	17.254435000	H	7.673508000	6.330883000	15.489160000
H	8.461116000	16.801156000	14.070742000	C	-0.726668000	14.013371000	18.574538000
H	6.133762000	17.536277000	15.244321000	C	0.490084000	14.671671000	18.336430000
H	4.437797000	10.126388000	16.978935000	C	0.601332000	15.628734000	17.310269000
H	3.924162000	8.654828000	11.597752000	C	-0.532061000	15.938670000	16.522980000
H	3.963629000	9.802641000	6.747302000	C	-1.750762000	15.280701000	16.773166000
H	4.030791000	6.819502000	9.895540000	C	-1.850593000	14.318795000	17.790307000
H	9.736855000	11.590054000	13.928445000	C	1.906917000	16.345900000	17.016858000
H	11.513246000	9.810662000	13.830591000	C	-0.388852000	16.973223000	15.421818000
H	6.795891000	14.421090000	8.701814000	C	0.547562000	18.100120000	15.819089000
H	8.475284000	14.396593000	6.841119000	C	1.682652000	17.789755000	16.604101000
H	11.659454000	14.367695000	9.785357000	C	2.566586000	18.818772000	16.979459000
H	4.044076000	7.426251000	7.449465000	H	3.441637000	18.578543000	17.595602000
H	10.958084000	7.444954000	14.349542000	C	2.337316000	20.143430000	16.575762000
H	10.929745000	14.390980000	7.416026000	C	1.210629000	20.451049000	15.796514000
C	4.735134000	17.718950000	10.712231000	C	0.320533000	19.431730000	15.424515000
H	5.609620000	17.256474000	10.219031000	H	2.585600000	16.289720000	17.884458000
H	5.063641000	18.025245000	11.722341000	H	-0.798249000	13.269383000	19.375967000
H	1.786787000	13.516809000	13.570722000	H	1.366105000	14.442425000	18.955237000
H	4.808935000	13.417482000	16.640965000	H	-2.629871000	15.528091000	16.165972000
H	6.832784000	8.582954000	15.050175000	H	-2.805696000	13.814004000	17.974423000
N	3.822764000	11.891501000	8.326823000	H	0.027072000	16.472624000	14.521087000
H	3.925065000	12.097650000	7.335811000	H	3.033423000	20.934770000	16.876059000
H	3.861668000	12.638796000	9.021678000	H	1.021099000	21.484067000	15.483662000
N	10.109137000	14.330667000	11.898394000	H	-0.564513000	19.670641000	14.822580000
H	11.101723000	14.461922000	12.081890000	H	2.421957000	15.816583000	16.186732000
H	9.429279000	14.462817000	12.649126000	H	-1.374623000	17.369000000	15.125531000

S13. References.

[1] Gaussian 09, Revision A.1, M. J. Frisch, G. W. Trucks, H. B. Schlegel, G. E. Scuseria, M. A. Robb, J. R. Cheeseman, G. Scalmani, V. Barone, B. Mennucci, G. A. Petersson, H. Nakatsuji, M. Caricato, X. Li, H. P. Hratchian, A. F. Izmaylov, J. Bloino, G. Zheng, J. L. Sonnenberg, M. Hada, M. Ehara, K. Toyota, R. Fukuda, J. Hasegawa, M. Ishida, T. Nakajima, Y. Honda, O. Kitao, H. Nikai, T. Vreven, J. A. Montgomery, J. E. Peralta, F. Ogliaro, M. Bearpark, J. J. Heyd, E. Brothers, K. N. Kudin, V. N. Staroverov, T. Keith, R. Kobayashi, J. Normand, K. Raghavachari, A. Rendell, J. C. Burant, S. S. Iyengar, J. Tomasi, M. Cossi, N. Rega, J. M. Millam, M. Klene, J. E. Knox, J. B. Cross, V. Bakken, C. Adamo, J. Jaramillo, R. Gomperts, R. E. Stratmann, O. Yazyev, A. J. Austin, R. Cammi, C. Pomelli, J. W. Ochterski, R. L. Martin, K. Morokuma, V. G. Zakrzewski, G. A. Voth, P. Salvador, J. J. Dannenberg, S. Dapprich, A. D. Daniels, O. Farkas, J. B. Foresman, J. V. Ortiz, J. Cioslowski, D. J. Fox, **2015**.

[2] J. Tao, J. P. Perdew, V. N. Staroverov, G. E. Scuseria, *Phys. Rev. Lett.* **2003**, *91*, 146401–146404

[3] F. Weigend, *Phys. Chem. Chem. Phys.* **2006**, *8*, 1057–1065.

[4] F. Weigend, R. Ahlrichs, *Phys. Chem. Chem. Phys.* **2005**, *7*, 3297–3305.

[5] A. V. Marenich, C. J. Cramer, D. G. Truhlar, *J. Phys. Chem. B* **2009**, *113*, 6378–6396.

[6] M. Álvarez-Moreno, C. de Graaf, N. López, F. Maseras, J. M. Poblet, C. Bo, *J. Chem. Inf. Model.* **2015**, *55*, 95–103.

[7] P. Delgado, J. D. Martin-Romera, C. Perona, R. Vismara, S. Galli, C. R. Maldonado, F. J. Carmona, N. M. Padial, J. A. R. Navarro. *ACS Appl. Mater.* **2022**, *14*, 26501–26506.

[8] Ooi, T.; Takahashi, M.; Yamada, M.; Omoto, K.; Maruoka, K. *J. Am. Chem. Soc.* **2004**, *126*, 1150-1160.

[9] M. Utsunomiya, R. Kondo, T. Oshima, M. Safumi, T. Suzuki, Y. Obora, *Chem. Commun.* **2021**, *57*, 5139–5142.

[10] Costentin, C.; Savéant, J. M. Elements of Molecular and Biomolecular Electrochemistry: An Electrochemical Approach to Electron Transfer Chemistry, 2nd ed.; John Wiley & Sons: Hoboken, NJ, 2006

[11] D. A. Prystupa, A. Anderson, B. H. Torrie, *Journal of Raman Spectroscopy* **1994**, *25*, 175–182

[12] Scott A. Sandford *et al. ApJS* **2013**, *205*, 8

[13] M. Khatua, B. Goswami, S. Hans, Kamal, S. Mazumder, S. Samanta, *Inorg. Chem.* **2022**, *61*, 17777–17789.

[14] P. V. Ramachandran, A. A. Alawaed, H. J. Hamann, *Org. Lett.* **2022**, *24*, 8481–8486.

[15] A. P. Dieskau, J.-M. Begouin, B. Plietker, *European Journal of Organic Chemistry* **2011**, *2011*, 5291–5296

[16] M. E. González-Núñez, R. Mello, A. Olmos, R. Acerete, G. Asensio, *J. Org. Chem.* **2006**, *71*, 1039–1042

[17] Y.-F. Hou, S.-L. Zhang, *European Journal of Organic Chemistry* **2024**, *27*, e202400097

[18] N. Hirbawi, P. C. Lin, E. R. Jarvo, *J. Org. Chem.* **2022**, *87*, 12352–12369

[19] B. White, T. Dudding, *J. Org. Chem.* **2024**, *89*, 4569–4578

[20] S. A. Cook, J. W. Ziller, A. S. Borovik, *Inorg. Chem.* **2014**, *53*, 11029–11035

[21] S. Bishi, B. Sankar Lenka, P. Kreitmeier, O. Reiser, D. Sarkar, *Advanced Synthesis & Catalysis* **2024**, *366*, 3397–3403
